# Selective serotonin reuptake inhibitor use during early pregnancy and congenital malformations: a systematic review and meta-analysis of cohort studies of more than 9 million births

**DOI:** 10.1186/s12916-018-1193-5

**Published:** 2018-11-12

**Authors:** Shan-Yan Gao, Qi-Jun Wu, Ce Sun, Tie-Ning Zhang, Zi-Qi Shen, Cai-Xia Liu, Ting-Ting Gong, Xin Xu, Chao Ji, Dong-Hui Huang, Qing Chang, Yu-Hong Zhao

**Affiliations:** 1 Department of Clinical Epidemiology, Shengjing Hospital of China Medical University, No. 36, San Hao Street, Shenyang, Liaoning China; 2 Department of Pediatrics, Shengjing Hospital of China Medical University, Shenyang, China; 3 Department of Obstetrics and Gynecology, Shengjing Hospital of China Medical University, Shenyang, China

**Keywords:** Antidepressant, Congenital malformations, Cohort studies, Pregnancy, Serotonin uptake inhibitors, Meta-analysis

## Abstract

**Background:**

In 2005, the FDA cautioned that exposure to paroxetine, a selective serotonin reuptake inhibitor (SSRI), during the first trimester of pregnancy may increase the risk of cardiac malformations. Since then, the association between maternal use of SSRIs during pregnancy and congenital malformations in infants has been the subject of much discussion and controversy. The aim of this study is to systematically review the associations between SSRIs use during early pregnancy and the risk of congenital malformations, with particular attention to the potential confounding by indication.

**Methods:**

The study protocol was registered with PROSPERO (CRD42018088358). Cohort studies on congenital malformations in infants born to mothers with first-trimester exposure to SSRIs were identified via PubMed, Embase, Web of Science, and the Cochrane Library databases through 17 January 2018. Random-effects models were used to calculate summary relative risks (RRs).

**Results:**

Twenty-nine cohort studies including 9,085,954 births were identified. Overall, use of SSRIs was associated with an increased risk of overall major congenital anomalies (MCAs, RR 1.11, 95% CI 1.03 to 1.19) and congenital heart defects (CHD, RR 1.24, 95% CI 1.11 to 1.37). No significantly increased risk was observed when restricted to women with a psychiatric diagnosis (MCAs, RR 1.04, 95% CI 0.95 to 1.13; CHD, RR 1.06, 95% CI 0.90 to 1.26). Similar significant associations were observed using maternal citalopram exposure (MCAs, RR 1.20, 95% CI 1.09 to 1.31; CHD, RR 1.24, 95% CI 1.02 to 1.51), fluoxetine (MCAs, RR 1.17, 95% CI 1.07 to 1.28; CHD, 1.30, 95% CI 1.12 to 1.53), and paroxetine (MCAs, RR 1.18, 95% CI 1.05 to 1.32; CHD, RR 1.17, 95% CI 0.97 to 1.41) and analyses restricted to using women with a psychiatric diagnosis were not statistically significant. Sertraline was associated with septal defects (RR 2.69, 95% CI 1.76 to 4.10), atrial septal defects (RR 2.07, 95% CI 1.26 to 3.39), and respiratory system defects (RR 2.65, 95% CI 1.32 to 5.32).

**Conclusions:**

The evidence suggests a generally small risk of congenital malformations and argues against a substantial teratogenic effect of SSRIs. Caution is advisable in making decisions about whether to continue or stop treatment with SSRIs during pregnancy.

**Electronic supplementary material:**

The online version of this article (10.1186/s12916-018-1193-5) contains supplementary material, which is available to authorized users.

## Background

Selective serotonin reuptake inhibitors (SSRIs) have become the first-line pharmaceuticals for the treatment of depression, anxiety, and other psychiatric disorders since they were introduced into the market [1]. About 63% to 85% of pregnant women with exposure to antidepressant are treated with SSRIs [2–4]. SSRIs are thought to be effective for treating psychiatric disorders by increasing the synaptic bioavailability of the neurotransmitter serotonin (5-HT), which readily crosses the placenta and can affect certain kinds of cells and tissues during embryogenesis, which may result in certain congenital malformations, especially cardiac malformations [5–8]. In December 2005, the US Food and Drug Administration (FDA) cautioned that the use of paroxetine, as individual SSRI during the first trimester of pregnancy may increase the risk of cardiac malformations [9]. Since then, the associations between the use of SSRIs during pregnancy and the risk of congenital malformations in offspring have been the subject of much discussion and controversy [10].

The number of published meta-analyses regarding the associations between maternal use of SSRIs and congenital malformations has more than tripled during the last 5 years (20 meta-analyses to date). However, some of these studies produced conflicting results due to varying study designs and exposure times. Most inconsistently reported were congenital heart defects (CHD) (Additional file 1: Table S1). Furthermore, none of these meta-analyses attempted to comprehensively investigate the associations between the use of SSRIs and individual SSRIs and the risks of specific congenital malformations. Some of the previous meta-analyses [11–23] examined the risks of certain congenital malformations [overall major congenital anomalies (MCAs) and cardiac malformations] with maternal use of SSRIs and/or individual SSRIs. Other meta-analyses [24–28] examined the risks of specific (cardiac) malformations with the use of only one or two specific SSRIs. Reefhuis and colleagues [29] examined the risks of 15 congenital malformations categories with the use of individual SSRIs during early pregnancy; however, recall bias derived from case-control studies might have been inherent in those data. A large number of cohort studies were published recently that explore the aforementioned associations from Europe and other regions, but the results are still inconsistent [30–47].

Depression and anxiety have been associated with adverse pregnancy outcomes and health behaviors [48–50]. Thus, some researchers have expressed concerns that the underlying depression or psychiatric illness might increase the risks of congenital malformations in infants [51–53]. To the best of our knowledge, no previous meta-analyses have assessed potential confounding by indication (underlying psychiatric diagnosis) by comparing women using SSRIs vs. those with unmedicated psychiatric illness during the first trimester of pregnancy.

We performed a detailed systematic review and large-scale meta-analysis of current evidence from cohort studies to investigate whether there is any relationship between maternal use of SSRIs during early pregnancy and congenital malformations in infants. Particular attention is given to the potential for confounding by indication.

## Methods

The report of this systemic review and meta-analysis followed the recommendations of the Preferred Reporting Items for Systematic Reviews and Meta-Analyses (PRISMA) group [54]. Before study selection, the protocol for this review was registered with PROSPERO (CRD42018088358).

### Data sources and searches

PubMed, Embase, Web of Science, and The Cochrane Library were searched from database inception to 17 January 2018. The search strategy combined medical subject heading (MeSH) and Embase subject heading (EMTREE) terms with other unindexed or free-text terms. Details of the full search strategy are provided in Additional file 2. Reference lists of retrieved articles and previous systematic and narrative reviews were searched manually to retrieve all relevant documents. No language restrictions were imposed.

### Study selection

Cohort studies or randomized controlled trials that reported original data were eligible for inclusion if they reported any congenital malformations in infants born to mothers with any exposure to SSRIs or individual SSRIs (citalopram, fluoxetine, paroxetine, sertraline, escitalopram, or fluvoxamine) during the first trimester, had a comparison group that included pregnant women who were not exposed to any antidepressants and/or teratogens (folic acid antagonists, angiotensin-converting enzyme inhibitors, anticonvulsants, coumarin derivatives, and retinoids), and, if a risk estimate was not reported, provided necessary distribution of exposure, non-exposure, cases, and non-cases, from which a risk estimate could be calculated.

The titles and abstracts of retrieved articles were evaluated by two independent reviewers (S-YG and CS). The full texts of potentially eligible studies that seemed to meet the inclusion criteria were then obtained and independently reviewed by the two reviewers. Any disagreements were identified and resolved by discussion or by consultation with a third reviewer (Q-JW). If data were duplicated in more than one study, we included the study with the largest number of cases.

### Data extraction

A standardized, pre-designed spreadsheet was used for data extraction from the included studies. The study quality and synthesis of evidence were assessed. The following data were extracted into the spreadsheet: first author, publication year, geographic location, study period, data source, sample size (cases and cohorts), types of birth, definition of outcome, outcome with their risk estimates and 95% confidence intervals (CIs), and adjusted confounders. Congenital malformations were identified and defined according to the European Surveillance of Congenital Anomalies (EUROCAT) Guide 1.3 and ICD-10 and ICD-9 codes (Additional file 3: Table S1). The primary outcomes of interest were overall MCAs and specific CHD. The secondary outcomes of interest were other system-specific malformations (nervous system defects; eye defects; ear, face, and neck defects; respiratory system defects; orofacial cleft; digestive system defects; urogenital system defects; urinary system defects; genital system defects; musculoskeletal system defects; limb; and abdominal wall defects. Two reviewers (T-NZ and Z-QS) extracted data independently; any disagreements were resolved by discussion with a third reviewer (S-YG) where necessary.

For a study [36] that reported a different follow-up duration, the estimate of the follow-up duration during the first 6 years of life was extracted. For studies [31, 40, 44, 45, 47, 55–61] that did not report any adjusted risk estimate, we used the crude risk estimate. If a study lacked required data, they were requested by contacting the study authors by email.

### Risk of bias assessment

We used the Newcastle-Ottawa scale [62] to assess the risk of bias of cohort studies, which included studies based on the selection of study participant groups (four stars), the comparability of study groups (two stars), and the ascertainment of outcome (three stars). Studies were considered to have low risk of bias if they achieved a full rating in at least two categories of selection, comparability, or outcome assessment [63].

### Statistical analysis

For a study [64] that separately reported the risk estimates of SSRIs but did not report combined estimates, the effective count method proposed by Hamling et al. [65] was used to recalculate the effect estimate. Another study [56] reported results separately (but not combined) for CHD (bulbus cordis anomalies and anomalies of cardiac septal closure and other congenital anomalies of heart), nervous system malformations (spina bifida and other congenital anomalies of nervous system), digestive system defects (cleft palate and cleft lip, other congenital anomalies of upper alimentary tract, and other congenital anomalies of digestive system), and musculoskeletal system defects (certain congenital musculoskeletal deformities, other congenital musculoskeletal anomalies, and other congenital anomalies of limbs); here, the results were pooled using a fixed-effect model to obtain an overall combined estimate before combining these estimates with the remaining studies (Additional file 3: Table S1). Similar analyses also were performed for limb defects (limb reduction and clubfoot) [31, 34]. If the selected study did not include a risk estimate, the unadjusted risk estimate and the 95% CI were calculated from the raw data for simplicity [31, 38, 44, 45, 57, 59, 61, 66]. Because the odds ratio is an excellent approximation of the risk ratio in the case of rare outcomes, the results were referred to as relative risks (RRs) [67]; therefore, all results were reported as RR for simplicity. Estimates were pooled using the DerSimonian and Laird random-effects model to calculate summarized RRs and 95% CI [68].

We used the *I*^2^ statistic to assess heterogeneity in effect measures between the studies. *I*^2^ values of 25, 50, and 75% were considered to represent low, moderate, and high heterogeneity, respectively [69]. If ≥ 8 studies were available, potential sources of heterogeneity were explored by conducting subgroup analyses according to the following parameters: study quality (high risk vs. low risk), geographic location (Europe vs. Northern America or other regions), and adjustment for potential confounders (adjusted vs. unadjusted) including maternal age, socioeconomic status, smoking, alcohol drinking, body mass index (BMI) during pregnancy, pregnancy complications, and parity. Heterogeneity between subgroups was evaluated by meta-regression analysis. The potential for publication bias was examined through Begg’s and Egger’s tests [70, 71]. To determine the influence of an individual study in each main analysis of the estimated RR, we conducted a sensitivity analysis that recalculated the pooled effect by omitting one study at a time. Analyses were performed with Stata version 11.0 (StataCorp, College Station, TX). A two-tailed *P* value less than 0.05 was considered as statistically significant.

## Results

### Search results

**Fig. 1:**
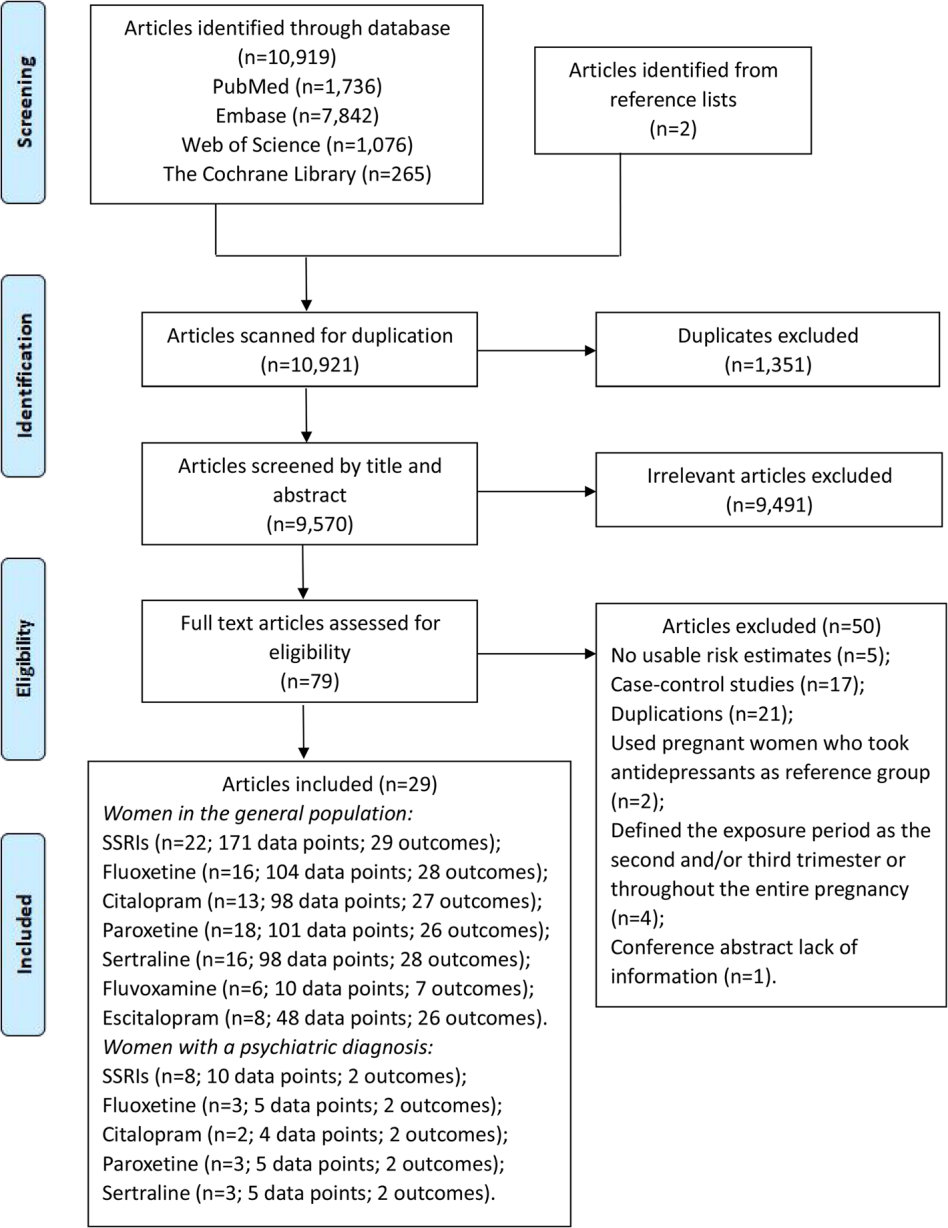
PRISMA of evidence search and selection for SSRIs use in early pregnancy and congenital malformations

We identified 10,919 potentially eligible articles in PubMed, Embase, Web of Science, and The Cochrane Library. Two additional studies were identified in a manual search of the reference lists. The titles and abstracts were screened, and 79 articles qualified for full-text review (Fig. 1). The authors of two studies failed to respond to requests for additional data. Finally, 29 cohort studies (published between 1996 and 2017) providing 649 data points that contributed to the quantitative synthesis met all inclusion and exclusion criteria, which included a total of 9,085,954 individuals for analysis. These included 25 studies focused on women in the general population, 8 studies focused on women with a psychiatric disorder, and 6 studies focused on both; 7,926,215 untreated pregnant women without psychiatric disorders, 1,916,076 SSRI-untreated women with psychiatric disorders, and 59,894 SSRI-treated women with psychiatric disorders; 7,590,399 individuals from Europe (15 studies), 1,206,094 from North America (10 studies), and 289,461 from Japan and Israel (4 studies). The key characteristics of the included studies are presented in Additional file 3: Table S2.

### Bias assessment

Analysis of the included studies using Newcastle-Ottawa criteria indicated that 23 studies were low risk and 6 were high risk for bias. All studies achieved a total score of 4 to 9 (median = 8) (Additional file 3: Table S3).

### Exposure to SSRIs

#### Risk of major congenital anomalies

**Fig. 2:**
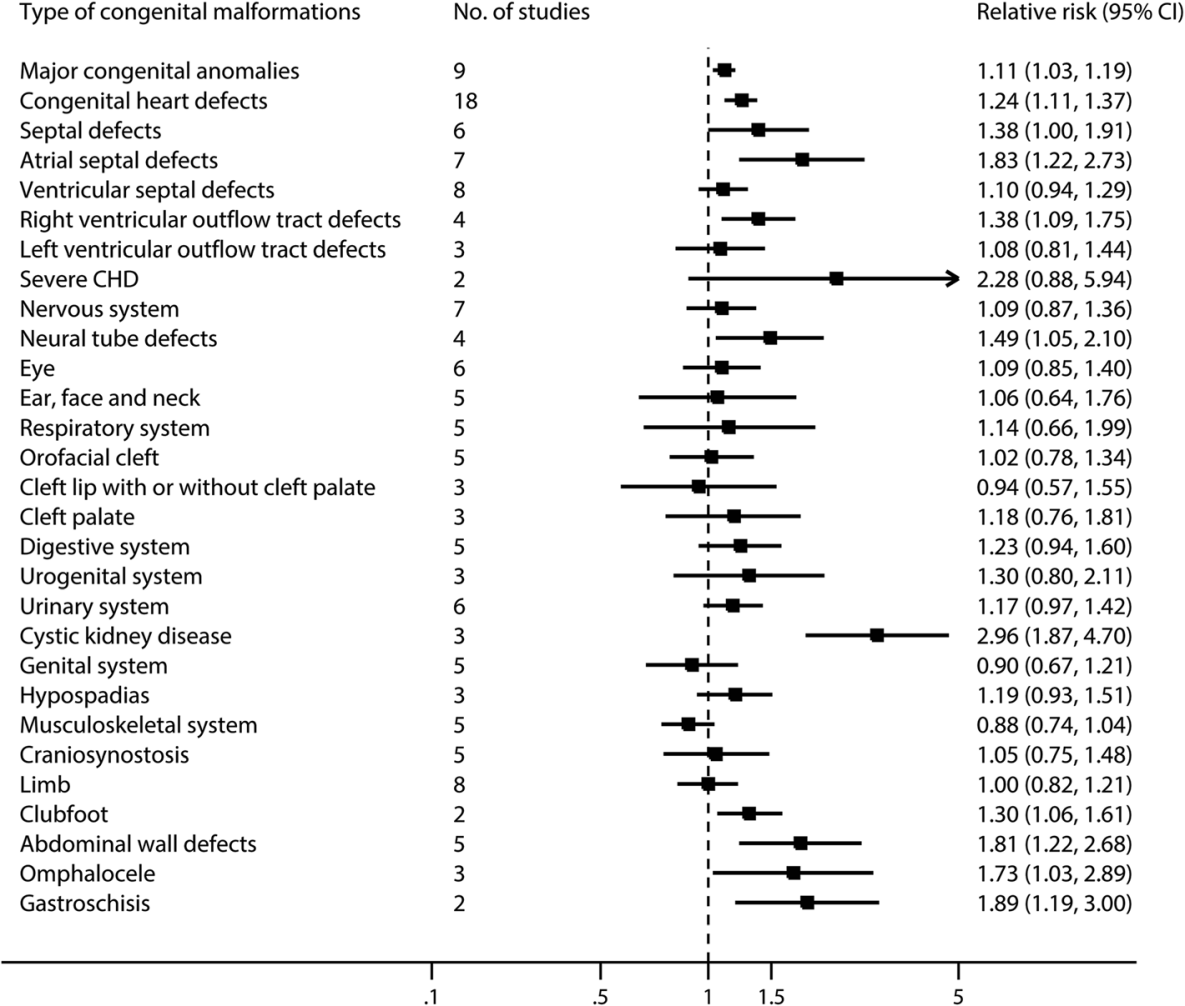
Risk of congenital malformations in infants, according to maternal exposure to SSRIs. Relative risks and 95% confidence intervals are presented to show the risk of congenital malformations among infants born to women with exposure to SSRIs during the first trimester, as compared with the risk among infants born to women in the general population without such exposure. SSRIs, selective serotonin reuptake inhibitors

**Fig. 3:**
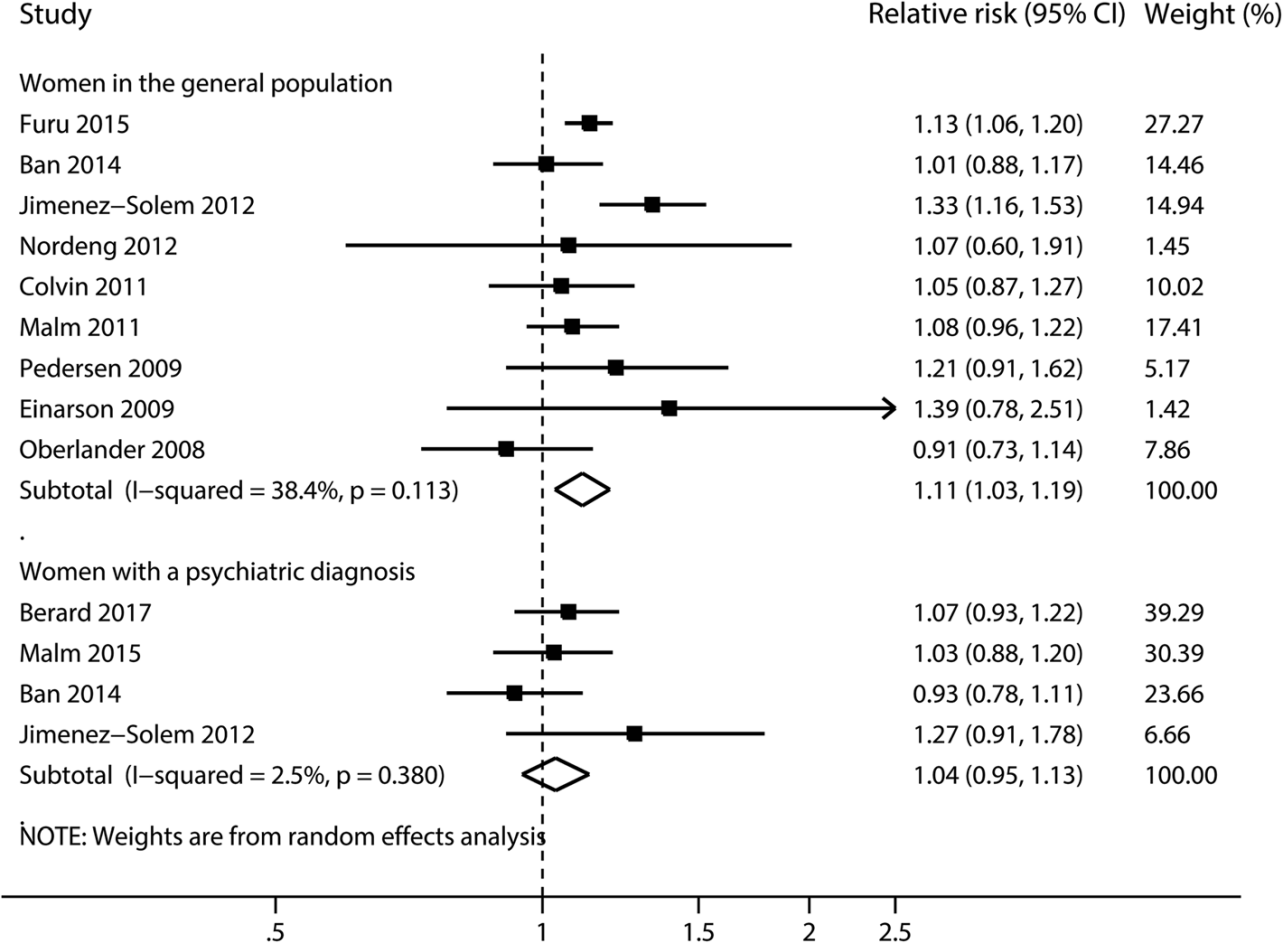
Risk of major congenital anomalies in infants, according to maternal exposure to SSRIs. Relative risks and 95% confidence intervals are presented to show the risk of major congenital anomalies among infants born to women with exposure to SSRIs during the first trimester, as compared with the risk among infants born to women without such exposure. SSRIs, selective serotonin reuptake inhibitors

Nine studies [34, 37–40, 43, 60, 61, 72] for the comparison of women receiving SSRIs versus women in the general population were included for this analysis. The pooled RR was 1.11 (95% CI 1.03 to 1.19, *I*^2^ = 38.4%, *P* = 0.11, Figs. 2 and 3, Additional file 3: Table S4), with no evidence of publication bias (Begg’s *P* = 0.92, Eggers’s *P* = 0.83). No significantly increased risk was observed when restricted to women with a psychiatric diagnosis (RR 1.04, 95% CI 0.95 to 1.13, *I*^2^ = 2.5%, *P* = 0.38, Fig. 3) [2, 33, 37, 72].

#### Risk of specific congenital heart defects

**Fig. 4:**
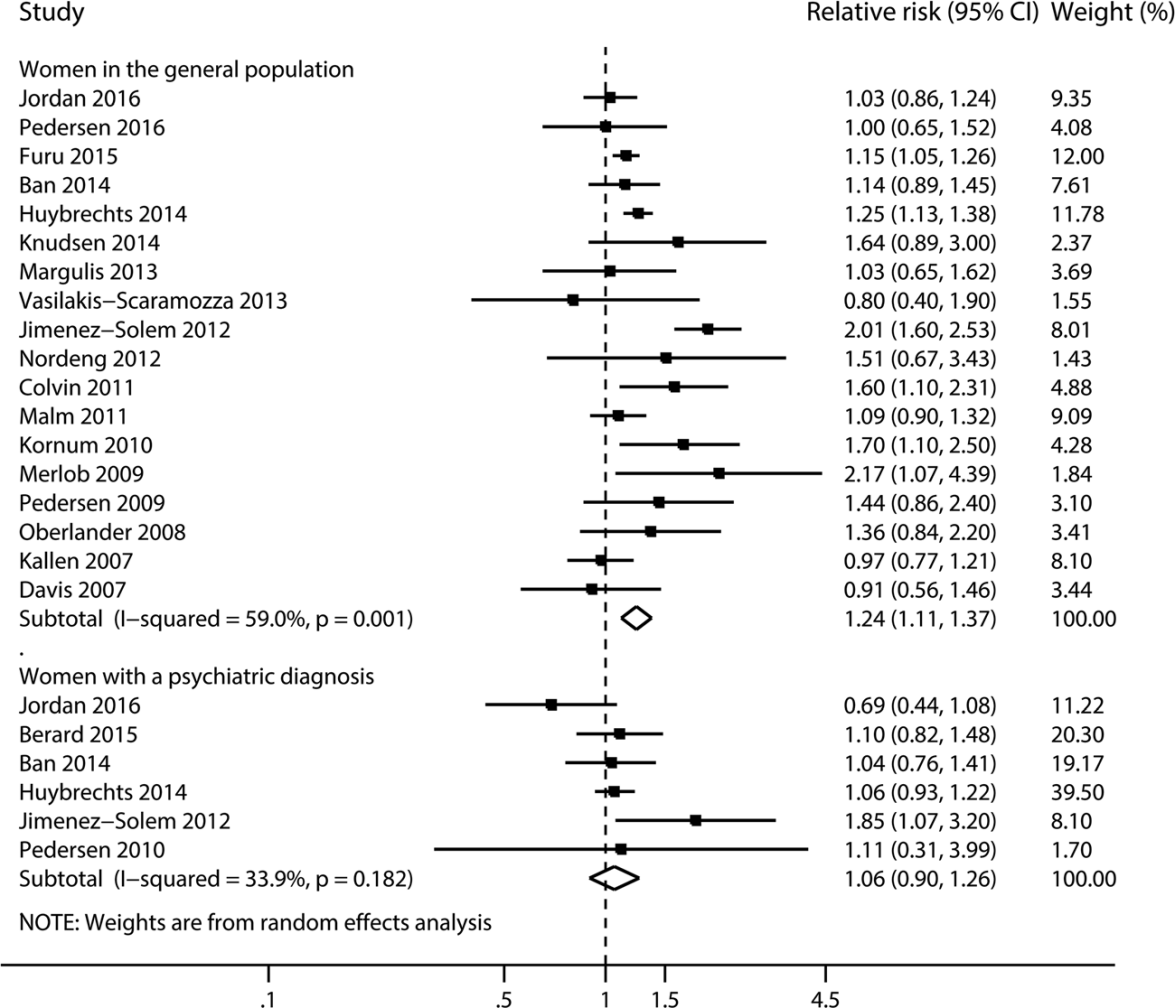
Risk of congenital heart defects in infants, according to maternal exposure to SSRIs. Relative risks and 95% confidence intervals are presented to show the risk of congenital heart defects among infants born to women with exposure to SSRIs during the first trimester, as compared with the risk among infants born to women without such exposure. SSRIs, selective serotonin reuptake inhibitors

Eighteen studies [31, 32, 34–40, 42–44, 46, 55, 56, 58, 61, 72] in the general population were included for this analysis. The pooled RR was 1.24 (95% CI 1.11 to 1.37, *I*^2^ = 59.0%, *P* = 0.001, Figs. 2 and 4, Additional file 3: Table S4), with no evidence of publication bias (Begg’s *P* = 0.23, Eggers’s *P* = 0.45). No significantly increased risk was observed when restricted to women with a psychiatric diagnosis (RR 1.06, 95% CI 0.90 to 1.26, *I*^2^ = 33.9%, *P* = 0.18, Fig. 4) [31, 37, 41, 55, 64, 72].

**Fig. 5:**
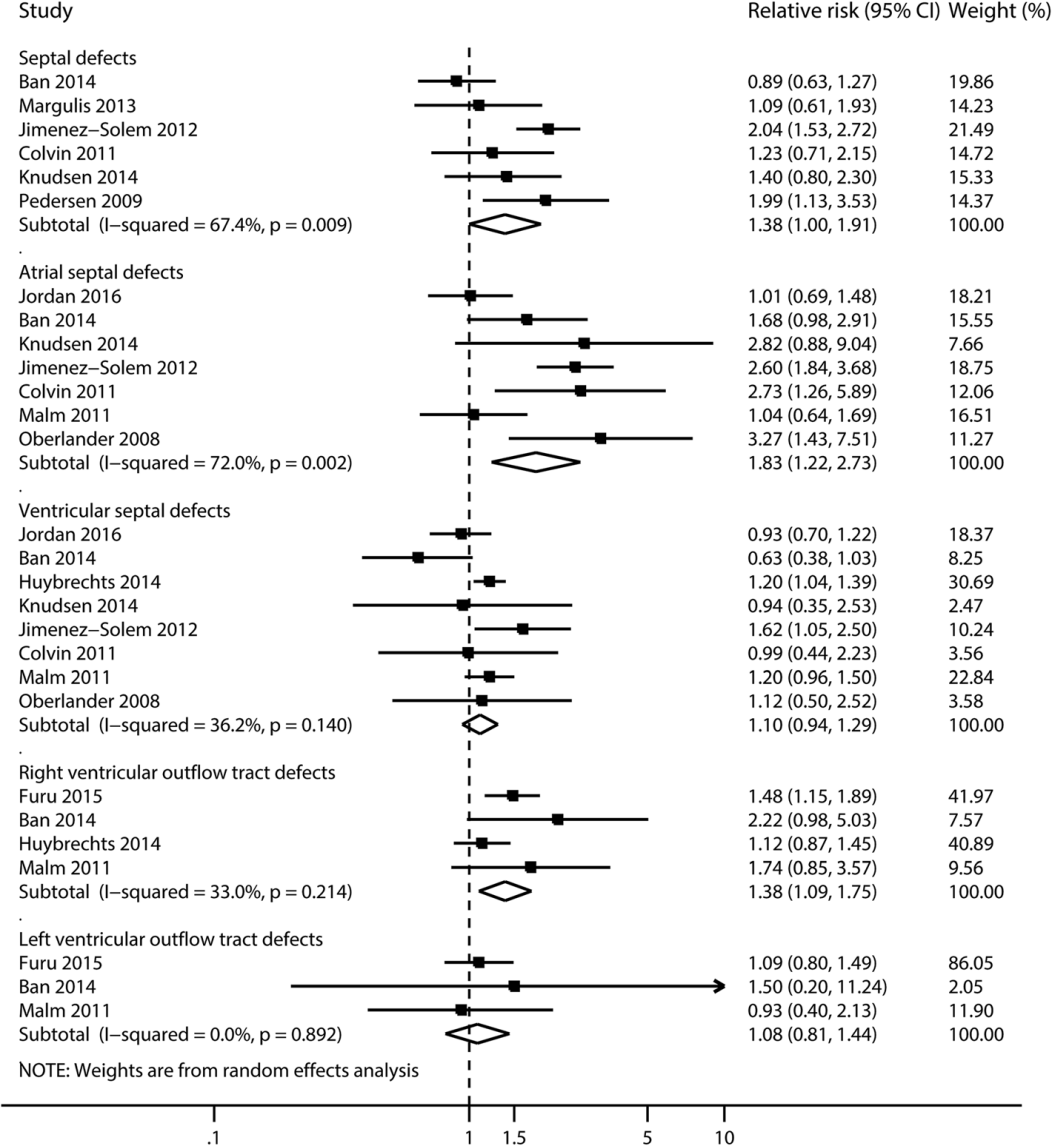
Risk of septal defects in infants, according to maternal exposure to SSRIs. Relative risks and 95% confidence intervals are presented to show the risk of septal defects among infants born to women with exposure to SSRIs during the first trimester, as compared with the risk among infants born to women in the general population without such exposure. SSRIs, selective serotonin reuptake inhibitors

Maternal use of SSRIs during the first trimester was associated with an increased risk in septal defects [36, 37, 40, 42, 43, 72] (RR 1.38, 95% CI 1.00 to 1.91, *I*^2^ = 67.4%, *P* = 0.009), atrial septal defects (ASD) [31, 35, 37, 39, 40, 61, 72] (RR 1.83, 95% CI 1.22 to 2.73, *I*^2^ = 72.0%, *P* = 0.002), and right ventricular outflow tract defects (RVOTD) [34, 39, 55, 72] (RR 1.38, 95% CI 1.09 to 1.75, *I*^2^ = 33.0%, *P* = 0.21) (Figs. 2 and 5; Additional file 3: Table S4). No evidence of publication bias was detected in any of these studies (all *P* > 0.05).

#### Risk of other system-specific malformations

Maternal use of SSRIs during the first trimester was associated with an increased risk of neural tube defects [31, 37, 39, 46] (RR 1.49, 95% CI 1.05 to 2.10, *I*^2^ = 0, *P* = 0.43), cystic kidney disease [34, 40, 46] (RR 2.96, 95% CI 1.87 to 4.70, *I*^2^ = 0, *P* = 0.81), clubfoot [31, 34] (RR 1.30, 95% CI 1.06 to 1.61, *I*^2^ = 0, *P* = 0.65), abdominal wall defects [30, 31, 37, 46, 72] (RR 1.81, 95% CI 1.22 to 2.68, *I*^2^ = 0, *P* = 0.86), omphalocele [31, 34, 39] (RR 1.73, 95% CI 1.03 to 2.89, *I*^2^ = 0, *P* = 0.73), and gastroschisis [31, 34] (RR 1.89, 95% CI 1.19 to 3.00, *I*^2^ = 0, *P* = 0.56) (Fig. 2, Additional file 3: Table S4).

### Exposure to individual SSRIs

#### Citalopram

Eight studies [34, 37, 39, 40, 43, 60, 61, 72] in the general population provided data for MCAs in infants. The pooled RR was 1.20 (95% CI 1.09 to 1.31, *I*^2^ = 13.4%, *P* = 0.33), with no evidence of publication bias (Begg’s *P* = 0.54, Eggers’s *P* = 0.77). No significantly increased risk was observed when restricted to women with a psychiatric diagnosis (RR 1.17, 95% CI 0.84 to 1.62, *I*^2^ = 66.0%, *P* = 0.09) [2, 72] (Additional file 3: Table S5, Additional file 4).

Eleven studies [31, 34, 37, 39, 40, 42–44, 46, 61, 72] provided data for CHD in infants in the general population. The pooled RR was 1.24 (95% CI 1.02 to 1.51, *I*^2^ = 52.5%, *P* = 0.02), with no evidence of publication bias (Begg’s *P* = 0.23, Eggers’s *P* = 0.32). No significantly increased risk was observed when restricted to women with a psychiatric diagnosis (RR 1.08, 95% CI 0.75 to 1.56, *I*^2^ = 0, *P* = 0.75) [2, 72] (Additional file 3: Table S5, Additional file 5).

Citalopram use during the first trimester was associated with an increased risk of septal defects [37, 40, 42, 43] (RR 1.81, 95% CI 1.22 to 2.68, *I*^2^ = 0, *P* = 0.55), RVOTD [34, 39] (RR 1.59, 95% CI 1.08 to 2.35, *I*^2^ = 0, *P* = 0.54), eye defects [31, 37, 40, 72] (RR 2.00, 95% CI 1.13 to 3.54, *I*^2^ = 0, *P* = 0.55), urinary system defects [31, 37, 40, 72] (RR 1.72, 95% CI 1.27 to 2.33, *I*^2^ = 0, *P* = 0.72), and hypospadias [31, 34] (RR 1.87, 95% CI 1.23 to 2.83, *I*^2^ = 0, *P* = 0.43) (Additional file 3: Table S5).

#### Fluoxetine

Eleven studies [34, 37–40, 43, 45, 57, 60, 61, 72] in the general population provided data for MCAs in infants. The pooled RR was 1.17 (95% CI 1.07 to 1.28, *I*^2^ = 0, *P* = 0.50), with no evidence of publication bias (Begg’s *P* = 0.28, Eggers’s *P* = 0.62). No significantly increased risk was observed when restricted to women with a psychiatric diagnosis (RR 0.84, 95% CI 0.67 to 1.05, *I*^2^ = 0, *P* = 0.83) [2, 72] (Additional file 3: Table S6, Additional file 6).

Fourteen studies [31, 34, 37–40, 42–46, 55, 61, 72] provided data for CHD in infants in the general population. The pooled RR was 1.30 (95% CI 1.12 to 1.53, *I*^2^ = 29.3%, *P* = 0.14), with no evidence of publication bias (Begg’s *P* = 0.23, Eggers’s *P* = 0.32). No significantly increased risk was observed when restricted to women with a psychiatric diagnosis (RR 0.94, 95% CI 0.65 to 1.37, *I*^2^ = 41.9%, *P* = 0.18) [2, 55, 72] (Additional file 3: Table S6, Additional file 7).

Fluoxetine use during the first trimester was associated with an increased risk of septal defects [37, 40, 42, 43] (RR 1.65, 95% CI 1.02 to 2.67, *I*^2^ = 0, *P* = 0.99), RVOTD [34, 39, 55] (RR 1.63, 95% CI 1.11 to 2.41, *I*^2^ = 18.0%, *P* = 0.30), neural tube defects [31, 37, 39] (RR 2.28, 95% CI 1.28 to 4.06, *I*^2^ = 0, *P* = 0.76), and ear, face, and neck defects [31, 40] (RR 3.45, 95% CI 1.28 to 9.29, *I*^2^ = 0, *P* = 0.41) (Additional file 3: Table S6).

#### Paroxetine

Eleven studies [34, 37–40, 43, 45, 47, 60, 61, 72] provided data for MCAs in infants in the general population. The pooled RR was 1.18 (95% CI 1.05 to 1.32, *I*^2^ = 0, *P* = 0.64), with no evidence of publication bias (Begg’s *P* = 0.09, Eggers’s *P* = 0.14). No significantly increased risk was observed when restricted to women with a psychiatric diagnosis (RR 1.17, 95% CI 0.97 to 1.41, *I*^2^ = 0, *P* = 0.34) [2, 72] (Additional file 3: Table S7, Additional file 8).

Sixteen studies [31, 34, 37–40, 42–45, 55, 56, 61, 66, 72] in the general population provided data for CHD in infants. The pooled RR was 1.35 (95% CI 1.19 to 1.53, *I*^2^ = 0, *P* = 0.71), with no evidence of publication bias (Begg’s *P* = 0.69, Eggers’s *P* = 0.21). No significantly increased risk was observed when restricted to women with a psychiatric diagnosis (RR 1.27, 95% CI 0.89 to 1.80, *I*^2^ = 72.3%, *P* = 0.03) [2, 55, 72] (Additional file 3: Table S7, Additional file 9).

Paroxetine use during the first trimester was associated with an increased risk of RVOTD [34, 39, 55] (RR 2.15, 95% CI 1.04 to 4.44, *I*^2^ = 67.0%, *P* = 0.049), eye defects [31, 56, 72] (RR 2.26, 95% CI 1.26 to 4.04, *I*^2^ = 0, *P* = 0.53), and cleft palate [31, 39] (RR 2.82, 95% CI 1.26 to 6.32, *I*^2^ = 0, *P* = 0.83) (Additional file 3: Table S7).

#### Sertraline

Nine studies [34, 37–40, 43, 60, 61, 72] provided data for MCAs in infants. The pooled RR was 1.10 (95% CI 0.99 to 1.22, *I*^2^ = 0, *P* = 0.69), with no evidence of publication bias (Begg’s *P* = 0.92, Eggers’s *P* = 0.85). No significantly increased risk was observed when restricted to women with a psychiatric diagnosis (RR 1.12, 95% CI 0.87 to 1.44, *I*^2^ = 0, *P* = 0.79) [2, 72] (Additional file 3: Table S8, Additional file 10).

Thirteen studies [31, 34, 37–40, 42–44, 46, 55, 61, 72] provided data for CHD in infants. The pooled RR was 1.42 (95% CI 1.12 to 1.80, *I*^2^ = 63.9%, *P* = 0.001), with no evidence of publication bias (Begg’s *P* = 0.50, Eggers’s *P* = 0.26). No significantly increased risk was observed when restricted to women with a psychiatric diagnosis (RR 1.12, 95% CI 0.92 to 1.35, *I*^2^ = 0, *P* = 0.80) [2, 55, 72] (Additional file 3: Table S8, Additional file 11).

Sertraline use during the first trimester was associated with an increased risk of septal defects [37, 40, 42, 43] (RR 2.69, 95% CI 1.76 to 4.10, *I*^2^ = 16.8%, *P* = 0.31), ASD [31, 37, 39, 40] (RR 2.07, 95% CI 1.26 to 3.39, *I*^2^ = 0, *P* = 0.54), respiratory system defects [37, 39, 40, 72] (RR 2.65, 95% CI 1.32 to 5.32, *I*^2^ = 0, *P* = 0.45), limb defects [31, 34, 37, 72] (RR 1.42, 95% CI 1.03 to 1.95, *I*^2^ = 0, *P* = 0.54), and clubfoot [31, 34] (RR 1.72, 95% CI 1.11 to 2.65, *I*^2^ = 0, *P* = 0.77) (Additional file 3: Table S8).

#### Escitalopram/fluvoxamine

Maternal use of escitalopram during the first trimester was associated with an increased risk of clubfoot [31] (RR 2.18, 95% CI 1.16 to 4.08), abdominal wall defects [31] (RR 3.52, 95% CI 1.56 to 7.93), and gastroschisis [31] (RR 3.95, 95% CI 1.46 to 10.68) (Additional file 3: Table S9). There was no statistically significant association between first-trimester exposure to fluvoxamine and MCAs [34, 39, 60, 61] (RR 0.77, 95% CI 0.49 to 1.21, *I*^2^ = 0, *P* = 0.79, Additional file 3: Table S10).

### Subgroup and sensitivity analyses

The results of subgroup and meta-regression analyses are presented in Additional file 3: Table S11-S15. Subgroup analyses indicated that the low risk of bias studies and European studies were generally consistent with the main results; however, they were not all statistically significant. No statistically significant source of heterogeneity was identified in meta-regression analyses. The sensitivity analysis omitted one study at a time, which showed the results appeared to be robust to the influence of individual studies. By contrast, the pooled RR of MCAs was 1.06 with SSRIs (95% CI 0.85 to 1.32, *I*^2^ = 0, *P* = 0.67) after excluding the study by Huybrechts et al. [55].

## Discussion

This comprehensive systemic review and meta-analysis of cohort studies including more than nine million births found generally small increased risks in 18 of 29 congenital malformations categories in infants born to mothers with exposure to SSRIs and individual SSRIs during early pregnancy, especially for MCAs and CHD. We found the RRs for the association between use of SSRIs and outcomes were lower in the restricted cohorts. Though the 95% CIs of the comparisons made between studies in general population and studies in mothers with a psychiatric diagnosis contained an overlap, we still cannot exclude the possibility of confounding by indication.

### SSRIs and congenital malformations

We identified a small but significant association between maternal use of SSRIs during the first trimester and MCAs in infants. This observed increase was consistent with the results of previous meta-analyses [15, 19] However, the association was attenuated after controlling for psychiatric diagnosis. Although the effects of SSRIs could never be completely separated from a psychiatric illness itself, such estimates are likely to be influenced by potential confounding by indication. Jimenez-Solem and colleagues [37] first differentiated between the consequences of SSRI use and the underlying disease, and found an increased risk of MCAs among infants born to women who took SSRIs during the first trimester, whereas the correlation was not significant for women who paused their use of SSRIs during the first trimester in a nationwide cohort study. Ban and colleagues [72] compared risks between pregnant women with medicated and unmedicated depression based on a population-based cohort study. Although results for MCAs were not statistically significant, the point estimate slightly decreased in comparison with pregnant women treated with SSRIs.

There was an association between first-trimester exposure to SSRIs and the risk of CHD. Some biological evidence possibly supports the observed increase [8, 73], but the results were inconsistent in three meta-analyses [14, 15, 19]. This difference is probably due to variations in sample size, study design, and time of exposure. The meta-analysis by Wang and colleagues [14] included four population-based cohort studies enrolling 1,996,519 participants and found no significant associations between the use of SSRIs and heart defects (Additional file 1: Table S1). In recent years, fourfold population-based cohort studies including nearly eight million participants have been conducted to examine the aforementioned relationship, and the results were consistent with our primary results (our unreported data). Myles and colleagues [15] synthesized evidence from nine cohort studies combined with case-control studies; however, high heterogeneity might have been inherent in those data. Nikfar and colleagues [19] assessed the risk of cardiovascular malformations with the use of SSRIs during pregnancy, but not during the first trimester. Similarly, the association was markedly attenuated after controlling for psychiatric diagnosis.

### Individual SSRIs and congenital malformations

#### Citalopram

The observed increase in MCAs and CHD with maternal use of citalopram during the first trimester was inconsistent with all previous meta-analyses [11, 12, 14, 15]. A recent meta-analysis by Kang and colleagues [11], including five cohort studies and one case-control study, reported no significant associations between exposure to citalopram and the risk of CHD during pregnancy. However, over twofold cohort studies were eligible for inclusion in our analysis. Data for 2.3 million births from a previous cohort study [34] were re-analyzed by an aggregate meta-analysis by Selmer and colleagues [12]; this cohort study was included in our meta-analysis. Results between the meta-analysis and the included study were similar regarding the association of citalopram use and CHD. Our findings regarding the effect of citalopram use on neural tube defects and hypospadias were inconsistent with the study by Reefhuis and colleagues [29], which used Bayesian analysis to combine summarized results from published literature with data from the US National Birth Defects Prevention Study.

#### Fluoxetine

The main results of this meta-analysis of fluoxetine use during early pregnancy were consistent with our previous study [26] and a recent meta-analysis by Selmer and colleagues [12]. Our data on fluoxetine-associated ventricular septal defects (VSD) and RVOTD were consistent with the study of Reefhuis and colleagues [29]. Our data also showed significant associations between fluoxetine use and the risk of system-specific malformations (neural tube defects and ear, face, and neck defects). However, these results require corroboration due to the limited number of individual studies.

#### Paroxetine

Our data show a significant association between the use of paroxetine and the risks of MCAs and CHD. The results were consistent with a recent meta-analysis by Berard and colleagues [27], but were inconsistent with a previous meta-analysis by Bar-Oz and colleagues [22]. Bar-Oz and colleagues conducted the analysis in 2007, and thus only included three cohort and case-control studies. Two meta-analyses assessed the risk of system-specific malformations from exposure to paroxetine and yielded inconsistent results due to the varying study design. One meta-analysis of cohort and cases studies [27] identified an increased risk of septal defects and ASD with paroxetine use; the other meta-analysis of case-control studies [29] identified an increased risk of ASD, gastroschisis, and omphalocele with paroxetine use (but not cleft palate or hypospadias).

#### Sertraline

Although the findings of this meta-analysis were consistent with our previous study [25], our data regarding the association of sertraline use with septal defects were inconsistent with the results of Reefhuis and colleagues [29]. Our data also showed significant associations between sertraline use and the risk of system-specific malformations (respiratory system defect, limb defect, and clubfoot). These results require corroboration due to the limited number of individual studies.

#### Escitalopram

The main results of this meta-analysis were consistent with previous meta-analyses [12, 29] regarding the risks of CHD and septal defects from exposure to escitalopram during early pregnancy. One of our included studies [31] reported statistically significant associations between escitalopram use and the risk of system-specific malformations (limb defect, clubfoot, abdominal wall defects, and gastroschisis) in three population-based cohorts that included 519,117 fetuses and infants.

### Potential mechanism

Potential biological mechanisms of SSRI use and the increased risk of congenital malformations is based on studies of drug metabolite levels in cord blood in human [74]. In vitro*,* a growing body of evidence has suggested that the neurotransmitter serotonin (5-HT) plays a crucial role as a signaling molecule in cardiogenesis [8, 75]. Consequently, disruption of the 5-HT signaling caused by the use of SSRIs may result in several different types of CHD [73, 76].

### Strengths and limitations of the study

Our study has several strengths. First, this meta-analysis of current evidence from cohort studies includes the largest sample size (more than nine million births) analyzed to date and combines the results with the most comprehensive data related to associations between the use of SSRIs and all individual SSRIs during early pregnancy and the risks of 29 categories of congenital malformations. Second, this meta-analysis pays particular attention to the potential confounding by indication. Third, for ethical reasons, there are no randomized controlled trials; therefore, the quality of evidence from cohort studies could provide clinicians and pregnant women with a reference in clinical practice.

There are limitations in our meta-analysis related to evidence synthesis and quality. First, the definition of outcomes varied among studies, particularly the definition of CHD, which could contribute to the high heterogeneity in our study. CHD has a specific definition and coding in the EUROCAT guide, but not in the ICD codes; however, most of the individual studies defining outcomes were based on ICD codes such as ICD-10 and ICD-9 (Additional file 3: Table S1-S2). We also failed to find any specific coding in either the ICD codes or EUROCAT subgroups related to MCAs, septal defects, RVOTD, and left ventricular outflow tract defects. Furthermore, the definitions of outcomes might depend on the authors of individual studies. For example, Ban and colleagues [72] defined septal defects as ASD, VSD, and atrioventricular septal defects, whereas Pedersen and colleagues [43] defined the defects as ASD and VSD. Additionally, the follow-up duration may also be a potential source of heterogeneity. On the one hand, some types of CHD (e.g., VSDs) may be self-healing [77]. On the other hand, due to the serious malformations are usually symptomatic with early detection, whereas milder malformations are sometimes identified at later age [36].

Second, the majority of individual studies only included live births. Stillbirth, spontaneous abortion, or induced abortion caused by severe malformations [78, 79] were not always recorded or observable and would have been missed, which could introduce selection bias and underestimate the strength of the associations between the use of SSRIs during early pregnancy and congenital malformations in infants [80]. The restriction of results according to different data sources also could also result in potential bias. The pooled effects of this study were dominated by record-linkage studies. However, data collected from prescription registries, dispensation registries, or drug reimbursement registries that rely on dispensed prescription information to determine maternal use of SSRIs would lead to misclassification of exposure [81, 82]. The dispensing of SSRIs may not always precisely reflect the specific time of exposure or verify that SSRIs were actually taken as prescribed. Selection bias presents a potential limitation in teratology information service studies, as women recruited during early pregnancy who feel the need for counseling about the teratogenic potential of SSRIs may be at higher risk than those who have no concerns [83].

Third, the event rate of congenital malformations in infants is very low, and individual studies may not have consistently adjusted for potential confounders. Therefore, we included adjusted or unadjusted risk estimates in our meta-analysis. Unadjusted risk estimates should be interpreted with caution, but the main results were still robust after removing crude risk estimates in the sensitivity analysis. Due to the lack of information on other potential confounders such as folic acid supplementation and familial-related factors, we could not fully rule out the possibility of residual confounding. For example, the study by Furu and colleagues [34] reported results from sibling design in addition to the full cohort. The results of sibling-controlled analyses showed attenuated risk compared with the full cohort. Thus, the small observed increased risk could be explained by familial-related factors or other lifestyle-related factors not adjusted for. In addition, we lacked information about the restricted cohorts regarding severity of disease. Eliminating the potential teratogenicity of SSRIs from a potential effect of the underlying psychiatric diagnoses remains a challenge.

Fourth, as we could not obtain an estimate for the incidence of MCAs and CHD events in the general population or in women with a psychiatric diagnosis, we could not provide an absolute risk increase of MCAs and CHD associated with exposure to SSRIs in the general population and in women with a psychiatric diagnosis. However, we obtained some examples from the published studies to give a suggestion of the increased absolute risk. Ban and colleagues [72] reported that children born to women with diagnosed depression unmedicated in early pregnancy had higher absolute risks of overall MCAs than children of mothers with no depression (absolute risk increase: 15 per 10000 births). Futhermore, Alwans et al. and Huybrechts et al. [55, 84] found a small increase in the absolute risk of CHD with exposure to SSRIs. Although the absolute risk of MCAs and CHD were highly likely to remain small, it is still of concern to pregnant women.

Fifth, it should be recognized that the implicated system-specific malformations and controlled psychiatric diagnosis studies are rare. The small number of included studies limited the statistical power of the study, which limited our ability to perform subgroup analyses to further investigate these issues and interpret the results. There was insufficient evidence to estimate fetal outcomes for the dosage of SSRIs use during pregnancy. Thus, we were unable to conduct a dose-response analysis.

Finally, since the study focused on non-exposure, i.e., pregnant women who were not exposed to any antidepressants and/or teratogens, rather than pregnant women who were exposed to other individual SSRIs, we could not determine if any of the individual SSRIs was preferable over others.

## Conclusions

The results of this meta-analysis highlight the complexity of this topic and the need to better understand the potential effect of the underlying psychiatric diagnosis. Continued evaluation of the association between maternal use of SSRIs and congenital malformations is warranted, and there is a pressing need for new studies on the effects of individual SSRIs (and their dosage) on system-specific malformations, specifically in women with underlying psychiatric diagnosis. The accumulated evidence suggests a generally small risk of congenital malformations and argues against a substantial teratogenic effect of SSRIs. Caution is advisable in making decisions about whether to continue or stopping treatment with SSRIs during pregnancy. Stopping treatment in mothers with major depression could be more harmful for the infant than continuing use of SSRIs. This information could be helpful for pregnant women and their healthcare providers to make more informed decisions about treatment.

## Additional files

Additional file 1: **Table S1.** Characteristics of prior meta-analyses of selective serotonin reuptake inhibitors (SSRIs) use in pregnancy and congenital malformations. (DOC 816 kb)

Additional file 2: Appendix 1. Search strategy. (DOCX 26 kb)

Additional file 3: **Table S1.** EUBOCAT Guide 1.3, ICD-10, and ICD-9 codes used to identify and define congenital malformations. **Table S2.** Characteristics of cohort studies of selective serotonin reuptake inhibitors (SSRIs) use in first-trimester and congenital malformations. **Table S3.** Risk of bias of included reports from cohort studies as assessed with the Newcastle-Ottawa scale. Table S4. Exposure to selective serotonin reuptake inhibitors (SSRIs) during the first trimester of pregnancy and risk of congenital malformations in infants: results of meta-analyses. **Table S5.** Exposure to citalopram during the first trimester of pregnancy and risk of congenital malformations in infants: results of meta-analyses. **Table S6.** Exposure to fluoxetine during the first trimester of pregnancy and risk of congenital malformations in infants: results of meta-analyses. **Table S7.** Exposure to paroxetine during the first trimester of pregnancy and risk of congenital malformations in infants: results of meta-analyses. **Table S8.** Exposure to sertraline during the first trimester of pregnancy and risk of congenital malformations in infants: results of meta-analyses. **Table S9.** Exposure to escitalopram during the first trimester of pregnancy and risk of congenital malformations in infants: results of meta-analyses. **Table S10.** Exposure to fluvoxamine during the first trimester of pregnancy and risk of congenital malformations in infants: results of meta-analyses. **Table S11.** Subgroup analysis of selective serotonin reuptake inhibitors (SSRIs) and risk of congenital malformations in infants: results of meta-analyses. **Table S12.** Subgroup analysis of citalopram and risk of congenital malformations in infants: results of meta-analyses. **Table S13.** Subgroup analysis of fluoxetine and risk of congenital malformations in infants: results of meta-analyses. **Table S14.** Subgroup analysis of paroxetine and risk of congenital malformations in infants: results of meta-analyses. **Table S15.** Subgroup analysis of sertraline and risk of congenital malformations in infants: results of meta-analyses. (DOC 1162 kb)

Additional file 4: **Figure S1.** Risk of major congenital anomalies in infants, according to maternal exposure to citalopram. (TIF 1083 kb)

Additional file 5: **Figure S2.** Risk of congenital heart defects in infants, according to maternal exposure to citalopram. (TIF 1123 kb)

Additional file 6: **Figure S3.** Risk of major congenital anomalies in infants, according to maternal exposure to fluoxetine. (TIF 1141 kb)

Additional file 7: **Figure S4.** Risk of congenital heart defects in infants, according to maternal exposure to fluoxetine. (TIF 1177 kb)

Additional file 8: **Figure S5.** Risk of major congenital anomalies in infants, according to maternal exposure to paroxetine. (TIF 1135 kb)

Additional file 9: **Figure S6.** Risk of congenital heart defects in infants, according to maternal exposure to paroxetine. (TIF 1244 kb)

Additional file 10: **Figure S7.** Risk of major congenital anomalies in infants, according to maternal exposure to sertraline. (TIF 1112 kb)

Additional file 11: **Figure S8.** Risk of congenital heart defects in infants, according to maternal exposure to sertraline. (TIF 1185 kb)

## Abbreviations

ASD: Atrial septal defect
CHD: Congenital heart defect
CIs: Confidence intervals
EUROCAT: European Surveillance of Congenital Anomalies
MCAs: Major congenital anomalies
RR: Relative risk
RVOTD: Right ventricular outflow tract defect
SSRIs: Selective serotonin reuptake inhibitors
VSD: Ventricular septal defect
