# Supplementary material for: Selective serotonin reuptake inhibitor use during early pregnancy and congenital malformations: a systematic review and meta-analysis of cohort studies of more than 9 million births
Source: BMC Med. 2018 Nov 12;16:205. doi: 10.1186/s12916-018-1193-5 (PMC6231277; doi:10.1186/s12916-018-1193-5)
Supplement: Supplementary file 1 — Table S1. Characteristics of prior meta-analyses of selective serotonin reuptake inhibitors (SSRIs) use in pregnancy and congenital malformations. (DOC 816 kb) [file 12916_2018_1193_MOESM1_ESM.doc]

**Additional file 1**

| **Table S1. Characteristics of prior meta-analyses of SSRIs use in pregnancy and congenital malformations.** | | | | | | | | |
| --- | --- | --- | --- | --- | --- | --- | --- | --- |
| **First author, year** | **Study period** | **Study design** | **Time of exposure** | **Exposure** | **No. of studies** | **Outcomes** | **Risk estimates  (95% CI)** | **Outcome definition** |
| Kang 2017 [25] | From July 1998 to July 2015 | Cohort or case-control | During pregnancy | Citalopram |  |  | Odds ratio | EUBOCAT |
|  |  |  |  |  | 8 | Major malformations | 1.07 (0.98 to 1.17) |  |
|  |  |  |  |  | 6 | Cardiac malformations | 1.31 (0.88 to 1.93) |  |
| Zhang 2017 [11] | From the database index date through December 31, 2015 | Cohort | During the first trimester | SSRIs |  |  | Relative risk | ICD-10,  ICD-9 codes |
|  |  |  |  |  | 18 | Cardiovascular-related malformations | 1.26 (1.13 to 1.39) |  |
|  |  |  |  |  | 6 | ASD | 2.06 (1.40 to 3.03) |  |
|  |  |  |  |  | 7 | VSD | 1.15 (0.97 to 1.36) |  |
|  |  |  |  |  | 14 | ASD and/or VSD | 1.27 (1.14 to 1.42) |  |
| Shen 2017 [20] | From the database index date through December 31, 2015 | Cohort | During the first trimester | Sertraline |  |  | Odds ratio | ICD-10,  ICD-9 codes |
|  |  |  |  |  | 12 | Cardiovascular-related malformations | 1.36 (1.06 to 1.74) |  |
|  |  |  |  |  | 8 | ASD and/or VSD | 1.36 (1.06 to 1.76) |  |
|  |  |  |  |  | 5 | Nervous system | 1.39 (0.83 to 2.32) |  |
|  |  |  |  |  | 5 | Digestive system | 1.23 (0.76 to 1.98) |  |
|  |  |  |  |  | 3 | Eye, ear, face and neck | 1.08 (0.33 to 3.55) |  |
|  |  |  |  |  | 5 | Urogenital system | 1.03 (0.73 to 1.46) |  |
|  |  |  |  |  | 5 | Musculoskeletal system | 0.97 (0.69 to 1.36) |  |
| Gao 2017 [21] | From inception to 21 March 2016 | Cohort | During the first trimester | Fluoxetine |  |  | Relative risk | ICD-10,  ICD-9 codes |
|  |  |  |  |  | 12 | Major malformations | 1.18 (1.08 to 1.29) |  |
|  |  |  |  |  | 12 | Cardiovascular malformations | 1.36 (1.17 to 1.59) |  |
|  |  |  |  |  | 7 | Septal defects | 1.38 (1.19 to 1.61) |  |
|  |  |  |  |  | 3 | Nervous system | 1.37 (0.83 to 2.25) |  |
|  |  |  |  |  | 3 | Eye | 1.30 (0.53 to 3.17) |  |
|  |  |  |  |  | 4 | Urogenital system | 1.02 (0.65 to 1.59) |  |
|  |  |  |  |  | 3 | Digestive system | 1.08 (0.60 to 1.96) |  |
|  |  |  |  |  | 3 | Respiratory system | 1.38 (0.69 to 2.78) |  |
|  |  |  |  |  | 4 | Musculoskeletal system | 0.82 (0.54 to 1.22) |  |
| Kowalik 2016* [13] | Through July 2015 | Population-based cohort studies | During pregnancy | SSRIs |  |  | Odds ratio | N/R |
|  |  |  |  |  | 8 | Congenital heart defects | 1.34 (1.11 to 1.61) |  |
| Selmer 2016† [12] | 1996-2010 | a Nordic register based cohort | During pregnancy |  |  |  | Odds ratio | ICD codes |
|  |  |  |  | SSRIs | N/R | Cardiovascular birth defect | 1.16 (1.06 to 1.27) |  |
|  |  |  |  |  |  | RVOTD | 1.53 (1.19 to 1.96) |  |
|  |  |  |  | Fluoxetine |  | Cardiovascular birth defect | 1.40 (1.15 to 1.70) |  |
|  |  |  |  | Citalopram |  | Cardiovascular birth defect | 1.15 (0.98 to 1.35) |  |
|  |  |  |  | Paroxetine |  | Cardiovascular birth defect | 1.33 (0.99 to 1.77) |  |
|  |  |  |  | Sertraline |  | Cardiovascular birth defect | 1.15 (0.94 to 1.40) |  |
|  |  |  |  | Escitalopram |  | Cardiovascular birth defect | 0.94 (0.68 to 1.30) |  |
| Berard 2016 [16] | Between 1966 to the 10 November 2015 | Cohort or case-control | During the first trimester | Paroxetine |  |  | Odds ratio | N/R |
|  |  |  |  |  | 15 | Major congenital malformations | 1.23 (1.10 to 1.38) |  |
|  |  |  |  |  | 18 | Cardiac malformations | 1.28 (1.11 to 1.47) |  |
|  |  |  |  |  | 8 | Bulbus cordis anomalies and anomalies of cardiac septal closure | 1.42 (1.07 to 1.89) |  |
|  |  |  |  |  | 4 | ASD | 2.38 (1.14 to 4.97) |  |
|  |  |  |  |  | 5 | VSD | 1.26 (0.69 to 2.32) |  |
|  |  |  |  |  | 4 | RVOTD | 2.29 (1.06 to 4.93) |  |
|  |  |  |  |  | 2 | LVOTD | 1.00 (0.38 to 2.61) |  |
|  |  |  |  |  | 4 | Other cardiac defects/other congenital anomalies of heart | 1.28 (0.96 to 1.69) |  |
| Reefhuis 2016‡ [29] | Between 1 October 1997 and 31 December 2009 | The National Birth  Defects Prevention Study based case-control | During early pregnancy |  |  |  | Odds ratio | ICD-9 codes |
|  |  |  |  | Citalopram | N/R | Neural tube defects | 1.8 (1.0 to 3.0) |  |
|  |  |  |  |  |  | VSD | 1.3 (0.9 to 1.8) |  |
|  |  |  |  |  |  | Cleft lip with or without cleft palate | 1.4 (0.7 to 2.7) |  |
|  |  |  |  |  |  | Hypospadias | 1.2 (0.7 to 2.0) |  |
|  |  |  |  | Escitalopram |  | Septal defects | 1.3 (0.7 to 2.1) |  |
|  |  |  |  | Fluoxetine |  | VSD | 1.4 (1.0 to 1.9) |  |
|  |  |  |  |  |  | RVOTD | 2.0 (1.4 to 3.1) |  |
|  |  |  |  |  |  | Esophageal atresia | 1.8 (0.8 to 3.4) |  |
|  |  |  |  |  |  | Craniosynostosis | 1.9 (1.1 to 3.0) |  |
|  |  |  |  | Paroxetine |  | Anencephaly | 3.2 (1.6 to 6.2) |  |
|  |  |  |  |  |  | ASD | 1.8 (1.1 to 3.0) |  |
|  |  |  |  |  |  | RVOTD | 2.4 (1.4 to 3.9) |  |
|  |  |  |  |  |  | Clef palate | 1.3 (0.7 to 2.3) |  |
|  |  |  |  |  |  | Hypospadias | 1.1 (0.6 to 1.9) |  |
|  |  |  |  |  |  | Gastroschisis | 2.5 (1.2 to 4.8) |  |
|  |  |  |  |  |  | Omphalocele | 3.5 (1.3 to 8.0) |  |
|  |  |  |  | Sertraline |  | Anencephaly | 1.2 (0.5 to 2.5) |  |
|  |  |  |  |  |  | Septal defects | 1.0 (0.8 to 1.4) |  |
|  |  |  |  |  |  | Anal atresia | 1.4 (0.8 to 2.3) |  |
|  |  |  |  |  |  | Any limb reduction | 1.2 (0.7 to 2.0) |  |
|  |  |  |  |  |  | Omphalocele | 1.4 (0.7 to 2.8) |  |
| Wang 2015 [14] | Through July 2014 | Population-based cohort studies | During the first trimester |  |  |  | Odds ratio | ICD-10,  ICD-9 codes |
|  |  |  |  | SSRIs | 4 | Heart defects | 1.06 (0.94 to 1.18) |  |
|  |  |  |  | Paroxetine | 3 | Heart defects | 0.97 (0.75 to 1.19) |  |
|  |  |  |  | Sertraline | 3 | Heart defects | 1.00 (0.81 to 1.20) |  |
|  |  |  |  | Fluoxetine | 3 | Heart defects | 1.11 (0.87 to 1.35) |  |
|  |  |  |  | Citalopram | 3 | Heart defects | 0.86 (0.56 to 1.16) |  |
| Myles 2013 [15] | Through June 2011 | Cohort or case-control | During the first trimester |  |  |  | Odds ratio | ICD codes |
|  |  |  |  | SSRIs | 16 | Major malformation | 1.10 (1.03 to 1.16) |  |
|  |  |  |  |  | 9 | Cardiac malformation | 1.15 (0.999 to 1.32) |  |
|  |  |  |  | Fluoxetine | 9 | Major malformation | 1.14 (1.01 to 1.30) |  |
|  |  |  |  |  | 6 | Cardiac malformation | 1.25 (0.98 to 1.60) |  |
|  |  |  |  | Paroxetine | 8 | Major malformation | 1.29 (1.11 to 1.49) |  |
|  |  |  |  |  | 8 | Cardiac malformation | 1.44 (1.12 to 1.86) |  |
|  |  |  |  | Sertraline | 6 | Major malformation | 1.01 (0.88 to 1.17) |  |
|  |  |  |  |  | 5 | Cardiac malformation | 0.93 (0.70 to 1.24) |  |
|  |  |  |  | Citalopram | 7 | Major malformation | 1.04 (0.92 to 1.17) |  |
|  |  |  |  |  | 6 | Cardiac malformation | 1.03 (0.80 to 1.32) |  |
| Ip 2013*‡ [84](1) | From 1974 to March 21, 2013 | Prospective | During the first trimester |  |  |  | Odds ratio | N/R |
|  |  |  |  | SSRIs | 8 | Fetal malformation | 1.72 (1.43 to 2.07) |  |
|  |  |  |  | Fluoxetine | N/R | Fetal malformation | 1.77 (1.01 to 2.93) |  |
|  |  |  |  | Paroxetine | N/R | Fetal malformation | 1.75 (1.06 to 2.95) |  |
| Yan 2013*[22] | Through March 1, 2011 | Cohort or case-control | During the first trimester |  |  |  | Odds ratio | N/R |
|  |  |  |  | Fluoxetine | 15 | Major malformations | 1.24 (1.08 to 1.44) |  |
|  |  |  |  |  | 15 | Cardiovascular defects | 1.60 (1.27 to 2.01) |  |
| Painuly 2013 [17] | Through August 2012 | Cohort or case-control | During the first trimester | Paroxetine |  |  | Relative risk | Conotruncal heart defects, septal heart defects, and ventricular outflow tract obstruction. |
|  |  |  |  |  | 11 | Cardiovascular malformations | 1.25 (1.01 to 1.54) |  |
| Riggin 2013 [23] | From inception to August 31, 2012 | Cohort | During the first trimester | Fluoxetine |  |  | Odds ratio | N/R |
|  |  |  |  |  | 18 | Major malformations | 1.12 (0.98 to 1.28) |  |
|  |  |  |  |  | 14 | Cardiac malformations | 1.60 (1.31 to 1.95) |  |
|  |  | Case–control |  |  | 2 | Major malformations | 3.72 (0.74 to 18.79) |  |
|  |  |  |  |  | 2 | Cardiac malformations | 0.63 (0.39 to 1.03) |  |
| Grigoriadis 2013 [18] | From inception to June 2010 | Cohort or case-control | During pregnancy | Paroxetine |  |  | Relative risk | Defined by the authors of the original publication. |
|  |  |  |  |  | 3 | Major malformations | 1.21 (0.94 to 1.56) |  |
|  |  |  |  |  | 5 | Cardiovascular malformations | 1.45 (1.06 to 1.99) |  |
|  |  |  |  |  | 2 | Septal heart defects | 0.78 (0.32 to 1.88) |  |
|  |  |  |  | Fluoxetine | 3 | Major malformations | 1.29 (1.03 to 1.61) |  |
|  |  |  |  |  | 3 | Cardiovascular malformations | 1.19 (0.83 to 1.72) |  |
|  |  |  |  |  | 2 | Septal heart defects | 1.18 (0.65 to 2.14) |  |
| Nikfar 2012§ [26] | From 1990 to March 2012 | Cohort | During pregnancy | SSRIs |  |  | Odds ratio | N/R |
|  |  |  |  |  | 21 | Major malformations | 1.27 (1.10 to 1.47) |  |
|  |  |  |  |  | 19 | Cardiovascular malformations | 1.19 (0.39 to 3.64) |  |
| Wurst 2010¶ [19] | From January 1, 1992, through September 30, 2008 | Cohort or case-control | During the first trimester | Paroxetine |  |  | Odds ratio | Any cardiac or congenital heart defects (defined by the original study authors). |
|  |  |  |  |  | 14 | Cardiac defects | 1.46 (1.17 to 1.82) |  |
| O'Brien 2008‖ [27] | Between January 1985 and November 2007 | Case-control | During the first trimester | Paroxetine |  |  | Odds ratio | N/R |
|  |  |  |  |  | 3 | Congential malformations | 1.18 (0.88 to 1.59) |  |
|  |  | Cohort |  |  |  |  | Risk difference |  |
|  |  |  |  |  | 6 | Cardiac malformation | 0.3% (-0.1–0.7%) |  |
| Bar-Oz 2007 [24] | From 1985 to 2006 | Cohort or case-control | During the first trimester | Paroxetine |  |  | Odds ratio | N/R |
|  |  |  |  |  | 3 | All major malformations | 1.54 (0.99 to 2.41) |  |
|  |  |  |  |  | 3 | Cardiac malformation | 3.47 (0.90 to 12.21) |  |
| Addis 2000 [28] | Through August 1996 | Prospective | During the first trimester | Fluoxetine |  |  | Odds ratio | N/R |
|  |  |  |  |  | 2 | Major malformations | 1.33 (0.49 to 3.58) |  |
| Abbreviations: ASD, Atrial septal defects; CI, Confidence intervals; LVOTD, Left ventricular outflow tract defects; N/R, Not reported; RVOTD, Right ventricular outflow tract defects; SSRIs, Selective serotonin reuptake inhibitors; VSD, Ventricular septal defects. *Conference abstract. † Reanalysed data from a previous study by Furu et al. [34], 2015 (a cohort study included in this meta-analysis).  ‡ Bayesian analysis.  § Nikfar et al.,2012* and Rahimi et al.,2006 not shown because of duplication.  ¶ Wurst et al.,2008* not shown because of duplication.  ‖ O'Brien et al.,2008* not shown because of duplication. | | | | | | | | |

References:

1. Ornoy A, Koren G. Selective serotonin reuptake inhibitors in human pregnancy: On the way to resolving the controversy. Semin Fetal Neonatal Med. 2014;19(3):188-94.

2. Berard A, Zhao J, Sheehy O. Antidepressant use during pregnancy and the risk of major congenital malformations in a cohort of depressed pregnant women: an updated analysis of the Quebec Pregnancy Cohort. BMJ Open. 2017;7(1):e013372.

3. Jimenez-Solem E, Andersen JT, Petersen M, Broedbaek K, Andersen NL, Torp-Pedersen C, et al. Prevalence of antidepressant use during pregnancy in Denmark, a nation-wide cohort study. PLoS One. 2013;8(4):e63034.

4. Taouk LH, Matteson KA, Stark LM, Schulkin J. Prenatal depression screening and antidepressant prescription: obstetrician-gynecologists' practices, opinions, and interpretation of evidence. Arch Womens Ment Health. 2018;21(1):85-91.

5. Liu Y, Zhou X, Zhu D, Chen J, Qin B, Zhang Y, et al. Is pindolol augmentation effective in depressed patients resistant to selective serotonin reuptake inhibitors? A systematic review and meta-analysis. Hum Psychopharmacol. 2015;30(3):132-42.

6. Hendrick V, Stowe ZN, Altshuler LL, Hwang S, Lee E, Haynes D. Placental passage of antidepressant medications. Am J Psychiatry. 2003;160(5):993-6.

7. Laine K, Heikkinen T, Ekblad U, Kero P. Effects of exposure to selective serotonin reuptake inhibitors during pregnancy on serotonergic symptoms in newborns and cord blood monoamine and prolactin concentrations. Arch Gen Psychiatry. 2003;60(7):720-6.

8. Sadler TW. Selective serotonin reuptake inhibitors (SSRIs) and heart defects: potential mechanisms for the observed associations. Reprod Toxicol. 2011;32(4):484-9.

9. U.S Food and Drug Administration (FDA). Public Health Advisory: Paroxetine. 2005. https://wayback.archiveit.org/7993/20170112033310/http://www.fda.gov/Drugs/DrugSafety/PostmarketDrugSafetyInformationforPatientsandProviders/ucm051731.htm. Accessed 27 Aug 2018.

10. Nembhard WN, Tang X, Hu Z, MacLeod S, Stowe Z, Webber D. Maternal and infant genetic variants, maternal periconceptional use of selective serotonin reuptake inhibitors, and risk of congenital heart defects in offspring: population based study. BMJ. 2017;356:j832.

11. Zhang TN, Gao SY, Shen ZQ, Li D, Liu CX, Lv HC, et al. Use of selective serotonin-reuptake inhibitors in the first trimester and risk of cardiovascular-related malformations: a meta-analysis of cohort studies. Sci Rep. 2017;7:43085.

12. Selmer R, Haglund B, Furu K, Andersen M, Nørgaard M, Zoëga H, et al. Individual-based versus aggregate meta-analysis in multi-database studies of pregnancy outcomes: the Nordic example of selective serotonin reuptake inhibitors and venlafaxine in pregnancy. Pharmacoepidemiol Drug Saf. 2016;25(10):1160-9.

13. Kowalik E, Ward K, Ye Y. SSRI use in pregnancy and congenital heart defects: A meta-analysis of population-based cohort studies. Pharmacotherapy. 2016;36(12):e302.

14. Wang S, Yang L, Wang L, Gao L, Xu B, Xiong Y. Selective Serotonin Reuptake Inhibitors (SSRIs) and the Risk of Congenital Heart Defects: A Meta-Analysis of Prospective Cohort Studies. J Am Heart Assoc. 2015;4(5):e001681.

15. Myles N, Newall H, Ward H, Large M. Systematic meta-analysis of individual selective serotonin reuptake inhibitor medications and congenital malformations. Aust N Z J Psychiatry. 2013;47(11):1002-12.

16. Bérard A, Iessa N, Chaabane S, Muanda FT, Boukhris T, Zhao JP. The risk of major cardiac malformations associated with paroxetine use during the first trimester of pregnancy: A systematic review and meta-analysis. Br J Clin Pharmacol. 2016;81(4):589-604.

17. Painuly N, Painuly R, Heun R, Sharan P. Risk of cardiovascular malformations after exposure to paroxetine in pregnancy: Meta-analysis. Psychiatrist. 2013;37(6):198-203.

18. Grigoriadis S, VonderPorten EH, Mamisashvili L, Roerecke M, Rehm J, Dennis CL, et al. Antidepressant exposure during pregnancy and congenital malformations: Is there an association? A systematic review and meta-analysis of the best evidence. J Clin Psychiatry. 2013;74(4):e293-308.

19. Wurst KE, Poole C, Ephross SA, Olshan AF. First trimester paroxetine use and the prevalence of congenital, specifically cardiac, defects: A meta-analysis of epidemiological studies. Birth Defects Res A Clin Mol Teratol. 2010;88(3):159-70.

20. Shen ZQ, Gao SY, Li SX, Zhang TN, Liu CX, Lv HC, et al. Sertraline use in the first trimester and risk of congenital anomalies: a systemic review and meta-analysis of cohort studies. Br J Clin Pharmacol. 2017;83(4):909-22.

21. Gao SY, Wu QJ, Zhang TN, Shen ZQ, Liu CX, Xu X, et al. Fluoxetine and congenital malformations: a systematic review and meta-analysis of cohort studies. Br J Clin Pharmacol. 2017;83(10):2134-47.

22. Yan Y, Cheng Y, Crowe B, Chhabra-Khanna R, Camporeale A, Marangell L. First trimester fluoxetine use and major malformations: A meta-analysis of epidemiological studies. Pharmacoepidemiol Drug Saf. 2013;22:168-9.

23. Riggin L, Frankel Z, Moretti M, Pupco A, Koren G. The fetal safety of fluoxetine: a systematic review and meta-analysis. Journal of obstetrics and gynaecology Canada. J Obstet Gynaecol Can. 2013;35(4):362-9.

24. Bar-Oz B, Einarson T, Einarson A, Boskovic R, O'Brien L, Malm H, et al. Paroxetine and congenital malformations: meta-Analysis and consideration of potential confounding factors. Clin Ther. 2007;29(5):918-26.

25. Kang HH, Ahn KH, Hong SC, Kwon BY, Lee EH, Lee JS, et al. Association of citalopram with congenital anomalies: A meta-analysis. Obstet Gynecol Sci. 2017;60(2):145-53.

26. Nikfar S, Rahimi R, Hendoiee N, Abdollahi M. Increasing the risk of spontaneous abortion and major malformations in newborns following use of serotonin reuptake inhibitors during pregnancy: A systematic review and updated meta-analysis. Daru. 2012;20(75).

27. O'Brien L, Einarson TR, Sarkar M, Einarson A, Koren G. Does Paroxetine Cause Cardiac Malformations? J Obstet Gynaecol Can. 2008;30(8):696-701.

28. Addis A, Koren G. Safety of fluoxetine during the first trimester of pregnancy: a meta-analytical review of epidemiological studies. Psychol Med. 2000;30(1):89-94.

29. Reefhuis J, Devine O, Friedman JM, Louik C, Honein MA. Specific SSRIs and birth defects: bayesian analysis to interpret new data in the context of previous reports. BMJ. 2015;351(h3190).

30. Nishigori H, Obara T, Nishigori T, Mizuno S, Metoki H, Hoshiai T, et al. Selective serotonin reuptake inhibitors and risk of major congenital anomalies for pregnancies in Japan: A nationwide birth cohort study of the Japan Environment and Children's Study. Congenit Anom. 2017;57(3):72-8.

31. Jordan S, Morris JK, Davies GI, Tucker D, Thayer DS, Luteijn JM, et al. Selective Serotonin Reuptake Inhibitor (SSRI) antidepressants in pregnancy and congenital anomalies: Analysis of linked databases in Wales, Norway and Funen, Denmark. PLoS One. 2016;11(12).

32. Petersen I, Evans SJ, Gilbert R, Marston L, Nazareth I. Selective serotonin reuptake inhibitors and congenital heart anomalies: Comparative cohort studies of women treated before and during pregnancy and their children. J Clin Psychiatry. 2016;77(1):e36-42.

33. Malm H, Sourander A, Gissler M, Gyllenberg D, Hinkka-Yli-Salomäki S, McKeague IW, et al. Pregnancy complications following prenatal exposure to SSRIs or maternal psychiatric disorders: Results from population-based national register data. Am J Psychiatry. 2015;172(12):1224-32.

34. Furu K, Kieler H, Haglund B, Engeland A, Selmer R, Stephansson O, et al. Selective serotonin reuptake inhibitors and venlafaxine in early pregnancy and risk of birth defects: population based cohort study and sibling design. BMJ. 2015;350:h1798.

35. Knudsen TM, Hansen AV, Garne E, Andersen AMN. Increased risk of severe congenital heart defects in offspring exposed to selective serotonin-reuptake inhibitors in early pregnancy - an epidemiological study using validated EUROCAT data. BMC Pregnancy Childbirth. 2014;14(1).

36. Margulis AV, Abou-Ali A, Strazzeri MM, Ding Y, Kuyateh F, Frimpong EY, et al. Use of selective serotonin reuptake inhibitors in pregnancy and cardiac malformations: A propensity-score matched cohort in CPRD. Pharmacoepidemiol Drug Saf. 2013;22(9):942-51.

37. Jimenez-Solem E, Andersen JT, Petersen M, Broedbaek K, Jensen JK, Afzal S, et al. Exposure to selective serotonin reuptake inhibitors and the risk of congenital malformations: A nationwide cohort study. BMJ open. 2012;2(3).

38. Nordeng H, Van Gelder MMHJ, Spigset O, Koren G, Einarson A, Eberhard-Gran M. Pregnancy outcome after exposure to antidepressants and the role of maternal depression: Results from the Norwegian mother and child cohort study. J Clin Psychopharmacol. 2012;32(2):186-94.

39. Malm H, Artama M, Gissler M, Ritvanen A. Selective serotonin reuptake inhibitors and risk for major congenital anomalies. Obstet Gynecol. 2011;118(1):111-20.

40. Colvin L, Slack-Smith L, Stanley FJ, Bower C. Dispensing patterns and pregnancy outcomes for women dispensed selective serotonin reuptake inhibitors in pregnancy. Birth Defects Res A Clin Mol Teratol. 2011;91(4):268.

41. Petersen I, Gilbert R, Evans S, Marston L, Nazareth I. SSRI and risk of congenital cardiac abnormalities. Pharmacoepidemiol Drug Saf. 2010;19:S211.

42. Kornum JB, Nielsen RB, Pedersen L, Mortensen PB, Norgaard M. Use of selective serotonin-reuptake inhibitors during early pregnancy and risk of congenital malformations: updated analysis. Clin Epidemiol. 2010;2:29-36.

43. Pedersen LH, Henriksen TB, Vestergaard M, Olsen J, Bech BH. Selective serotonin reuptake inhibitors in pregnancy and congenital malformations: population based cohort study. BMJ. 2009;339:b3569.

44. Merlob P, Birk E, Sirota L, Linder N, Berant M, Stahl B, et al. Are selective serotonin reuptake inhibitors cardiac teratogens? Echocardiographic screening of newborns with persistent heart murmur. Birth Defects Res A Clin Mol Teratol. 2009;85(10):837-41.

45. Diav-Citrin O, Shechtman S, Weinbaum D, Wajnberg R, Avgil M, Di Gianantonio E, et al. Paroxetine and fluoxetine in pregnancy: A prospective, multicentre, controlled, observational study. Br J Clin Pharmacol. 2008;66(5):695-705.

46. Kallen BA, Otterblad OP. Maternal use of selective serotonin re-uptake inhibitors in early pregnancy and infant congenital malformations. Birth Defects Res A Clin Mol Teratol.2007;79(4):301-8.

47. Vial T, Cournot MP, Bernard N, Carlier P, Jonville-Bero AP, Jean-Pastor MJ, et al. Paroxetine and congenital malformations: a prospective comparative study. Drug Safety. 2006;29(10):970.

48. Grote NK, Bridge JA, Gavin AR, Melville JL, Iyengar S, Katon WJ. A meta-analysis of depression during pregnancy and the risk of preterm birth, low birth weight, and intrauterine growth restriction. Arch Gen Psychiatry. 2010;67(10):1012-24.

49. Szegda K, Markenson G, Bertone-Johnson ER, Chasan-Taber L. Depression during pregnancy: a risk factor for adverse neonatal outcomes? A critical review of the literature. J Matern Fetal Neonatal Med. 2014;27(9):960-7.

50. Ogunyemi D, Jovanovski A, Liu J, Friedman P, Sugiyama N, Creps J, et al. The Contribution of Untreated and Treated Anxiety and Depression to Prenatal, Intrapartum, and Neonatal Outcomes. AJP Rep. 2018;8(3):e146-57.

51. Pedersen LH. The risks associated with prenatal antidepressant exposure: time for a precision medicine approach. Expert Opin Drug Saf. 2017;16(8):915-21.

52. Susser LC, Sansone SA, Hermann AD. Selective serotonin reuptake inhibitors for depression in pregnancy. Am J Obstet Gynecol. 2016;215(6):722-30.

53. Koren G, Nordeng H. Antidepressant use during pregnancy: The benefit-risk ratio. Am J Obstet Gynecol. 2012;207(3):157-63.

54. Moher D, Liberati A, Tetzlaff J, Altman DG. Preferred reporting items for systematic reviews and meta-analyses: the PRISMA statement. BMJ. 2009;339:b2535.

55. Huybrechts KF, Palmsten K, Avorn J, Cohen LS, Holmes LB, Franklin JM, et al. Antidepressant use in pregnancy and the risk of cardiac defects. N Engl J Med. 2014;370(25):2397-407.

56. Davis RL, Rubanowice D, McPhillips H, Raebel MA, Andrade SE, Smith D, et al. Risks of congenital malformations and perinatal events among infants exposed to antidepressant medications during pregnancy. Pharmacoepidemiol Drug Saf. 2007;16(10):1086-94.

57. Chambers CD, Johnson KA, Dick LM, Felix RJ, Jones KL. Birth outcomes in pregnant women taking fluoxetine. N Engl J Med. 1996;335(14):1010-5.

58. Vasilakis-Scaramozza C, Aschengrau A, Cabral H, Jick SS. Antidepressant use during early pregnancy and the risk of congenital anomalies. Pharmacotherapy. 2013;33(7):693-700.

59. Klieger-Grossmann C, Weitzner B, Panchaud A, Pistelli A, Einarson T, Koren G, et al. Pregnancy outcomes following use of escitalopram: A prospective comparative cohort study. J Clin Pharmacol. 2012;52(5):766-70.

60. Einarson A, Choi J, Einarson TR, Koren G. Incidence of major malformations in infants following antidepressant exposure in pregnancy: Results of a large prospective cohort study. Can J Psychiatry. 2009;54(4):242-6.

61. Oberlander TF, Warburton W, Misri S, Riggs W, Aghajanian J, Hertzman C. Major congenital malformations following prenatal exposure to serotonin reuptake inhibitors and benzodiazepines using population-based health data. Birth Defects Res B Dev Reprod Toxicol. 2008;83(1):68-76.

62. Wells GA, Shea BJ, O'Connell D, Peterson J, Welch V, Losos M, et al. The Newcastle-Ottawa Scale (NOS) for Assessing the Quality of Non-Randomized Studies in Meta-Analysis. Appl Eng Agric. 2014;18(6):727-34.

63. Odutayo A, Wong CX, Hsiao AJ, Hopewell S, Altman DG, Emdin CA. Atrial fibrillation and risks of cardiovascular disease, renal disease, and death: systematic review and meta-analysis. BMJ. 2016;354:i4482.

64. Bérard A, Zhao JP, Sheehy O. Sertraline use during pregnancy and the risk of major malformations. Am J Obstet Gynecol 2015;212(6):791-5.

65. Hamling J, Lee P, Weitkunat R, Ambuhl M. Facilitating meta-analyses by deriving relative effect and precision estimates for alternative comparisons from a set of estimates presented by exposure level or disease category. Stat Med. 2008;27(7):954-70.

66. Einarson A, Pistelli A, DeSantis M, Malm H, Paulus WD, Panchaud A, et al. Evaluation of the risk of congenital cardiovascular defects associated with use of paroxetine during pregnancy. Am J Psychiatry. 2008;165(6):749-52.

67. Rothman KJ, Development DD. Modern Epidemiology. 3rd Edition. Wolters Kluwer: Lippincott Williams & Wilkins; 2014.

68. Dersimonian R, Laird N. Meta-analysis in clinical trials. Controlled Clinical Trials. 1986;7(3):177.

69. Higgins JP, Thompson SG, Deeks JJ, Altman DG. Measuring inconsistency in meta-analyses. BMJ. 2003;327(7414):557-60.

70. Begg CB, Mazumdar M. Operating characteristics of a rank correlation test for publication bias. BIOMETRICS. 1994;50(4):1088-101.

71. Egger M, Davey SG, Schneider M, Minder C. Bias in meta-analysis detected by a simple, graphical test. BMJ. 1997;315(7109):629-34.

72. Ban L, Gibson JE, West J, Fiaschi L, Sokal R, Smeeth L, et al. Maternal depression, antidepressant prescriptions, and congenital anomaly risk in offspring: a population-based cohort study. BJOG. 2014;121(12):1471-81.

73. Sari Y, Zhou FC. Serotonin and its transporter on proliferation of fetal heart cells. Int J Dev Neurosci. 2003;21(8):417-24.

74. Sit DK, Perel JM, Helsel JC, Wisner KL. Changes in antidepressant metabolism and dosing across pregnancy and early postpartum. J Clin Psychiatry. 2008;69(4):652-8.

75. Yavarone MS, Shuey DL, Tamir H, Sadler TW, Lauder JM. Serotonin and cardiac morphogenesis in the mouse embryo. Teratology. 1993;47(6):573-84.

76. Choi DS, Kellermann O, Richard S, Colas JF, Bolanos-Jimenez F, Tournois C, et al. Mouse 5-HT2B receptor-mediated serotonin trophic functions. Ann N Y Acad Sci. 1998;861:67-73.

77. Gentile S. Early pregnancy exposure to selective serotonin reuptake inhibitors, risks of major structural malformations, and hypothesized teratogenic mechanisms. Expert Opin Drug Metab Toxicol. 2015;11(10):1585-97.

78. Carmi R, Gohar J, Meizner I, Katz M. Spontaneous abortion--high risk factor for neural tube defects in subsequent pregnancy. Am J Med Genet. 1994;51(2):93-7.

79. Bukowski R, Carpenter M, Conway D, Coustan D, Dudley DJ, Goldenberg RL, et al. Causes of Death Among Stillbirths. JAMA. 2011;306(22):2459-68.

80. Ehrenstein V, Sorensen HT, Bakketeig LS, Pedersen L. Medical databases in studies of drug teratogenicity: methodological issues. Clin Epidemiol. 2010;2:37-43.

81. Tuccori M, Montagnani S, Testi A, Ruggiero E, Mantarro S, Scollo C, et al. Use of selective serotonin reuptake inhibitors during pregnancy and risk of major and cardiovascular malformations: An update. Postgrad Med. 2010;122(4):49-65.

82. Alwan S, Friedman JM. Safety of selective serotonin reuptake inhibitors in pregnancy. CNS Drugs. 2009;23(6):493-509.

83. Alwan S, Friedman JM, Chambers C. Safety of Selective Serotonin Reuptake Inhibitors in Pregnancy: A Review of Current Evidence. CNS Drugs. 2016;30(6):499-515.

84. Ip Q, Smith KW, Malone DC: Bayesian analysis of malformation outcome in selective serotonin reuptake inhibitor (SSRI) use during pregnancy: An indirect comparison of citalopram, fluoxetine, paroxetine, and sertraline. Value in Health 2013; 16:A544.
