# Supplementary material for: Selective serotonin reuptake inhibitor use during early pregnancy and congenital malformations: a systematic review and meta-analysis of cohort studies of more than 9 million births
Source: BMC Med. 2018 Nov 12;16:205. doi: 10.1186/s12916-018-1193-5 (PMC6231277; doi:10.1186/s12916-018-1193-5)
Supplement: Supplementary file 2 — Appendix 1. Search strategy. (DOCX 26 kb) [file 12916_2018_1193_MOESM2_ESM.docx]

**Additional file 2**

**Appendix 1. Search strategy**

**ⅰ）Search strategy for PubMed**

The following searches were conducted in PubMed:

1."Serotonin Uptake Inhibitors"[Mesh] (39601)

2.5-Hydroxytryptamine Uptake Inhibitors (41715)

3.5 Hydroxytryptamine Uptake Inhibitors (41715)

4.Inhibitors, 5-HT Uptake (41715)

5.Inhibitors, 5 HT Uptake (41715)

6.Inhibitors, 5-Hydroxytryptamine Uptake (41715)

7.Inhibitors, 5 Hydroxytryptamine Uptake (41715)

8.Inhibitors, Serotonin Reuptake (45752)

9.Reuptake Inhibitors, Serotonin (45720)

10.Serotonin Reuptake Inhibitors (45720)

11.Uptake Inhibitors, 5-HT (41715)

12.Uptake Inhibitors, 5 HT (41715)

13.Uptake Inhibitors, 5-Hydroxytryptamine (41715)

14.Uptake Inhibitors, 5 Hydroxytryptamine (41715)

15.Uptake Inhibitors, Serotonin (41715)

16.5-HT Uptake Inhibitors (41721)

17.5 HT Uptake Inhibitors (41721)

18.Inhibitors, Serotonin Uptake (41715)

19.Selective Serotonin Reuptake Inhibitors (44875)

20.#1 OR #2 OR #3 OR #4 OR #5 OR #6 OR #7 OR #8 OR #9 OR #10 OR #11 OR #12 OR #13 OR #14 OR #15 OR #16OR #17 OR #18 OR #19 (45726)

21.Fluoxetine [Mesh] (8448)

22.Fluoxetin (12836)

23.N-Methyl-gamma-(4-(trifluoromethyl)phenoxy)benzenepropanamine (2)

24.Lilly-110140 (12839)

25.Lilly 110140 (12839)

26.Lilly110140 (12839)

27.Sarafem (12818)

28.Fluoxetine Hydrochloride (12818)

29.Prozac (12818)

30.#21 OR #22 OR #23 OR #24 OR #25 OR #26 OR #27 OR #28 OR #29 (12894)

31.Paroxetine [Mesh] (3778)

32.BRL-29060 (5931)

33.BRL 29060 (5931)

34.BRL29060 (5931)

35.FG-7051 (5932)

36.FG 7051 (5932)

37.FG7051 (5932)

38.Paroxetine Acetate (5931)

39.Paroxetine Hydrochloride (5931)

40.Paroxetine Hydrochloride Hemihydrate (5931)

41.Hemihydrate, Paroxetine Hydrochloride (5931)

42.Hydrochloride Hemihydrate, Paroxetine (5931)

43.Paroxetine Hydrochloride, Hemihydrate (5931)

44.Hemihydrate Paroxetine Hydrochloride (5931)

45.Hydrochloride, Hemihydrate Paroxetine (5931)

46.Seroxat (5934)

47.Paroxetine Maleate (5931)

48.Paroxetine, cis-(+)-Isomer (0)

49.Paroxetine, cis-(-)-Isomer (0)

50.Paroxetine, trans-(+)-Isomer (0)

51.Paxil (5941)

52.Aropax (5931)

53.Paroxetine Hydrochloride Anhydrous (5931)

54.#31 OR #32 OR #33 OR #34 OR #35 OR #36 OR #37 OR #38 OR #39 OR #40 OR #41 OR #42 OR #43 OR #44 OR #45 OR #46 OR #47 OR #48 OR #49 OR #50 OR #51 OR #52 OR #53 (5941)

55.Citalopram [Mesh] (4337)

56.Cytalopram (6254)

57.Lu-10-171 (6257)

58.Lu10171 (6252)

59.Escitalopram (6790)

60.Lexapro (6257)

61.#55 OR #56 OR #57 OR #58 OR #59 OR #60 (6798)

62.Escitalopram [Mesh] (4337)

63.Cytalopram (6254)

64.Lu-10-171 (6257)

65.Lu10171 (6252)

66.Escitalopram (6790)

67.Lexapro (6257)

68.#62 OR #63 OR #64 OR #65 OR #66 OR #67 (6798)

69.Sertraline [Mesh] (2754)

70.Zoloft (4579)

71.Altruline (4570)

72.Lustral (4571)

73.Apo-Sertraline (4570)

74.Apo Sertraline (4570)

75.Aremis (4575)

76.Besitran (4571)

77.Sealdin (4570)

78.Gladem (4571)

79.Novo-Sertraline (4570)

80.Novo Sertraline (4570)

81.ratio-Sertraline (4570)

82.ratio Sertraline (4570)

83.Rhoxal-sertraline (4570)

84.Rhoxal sertraline (4570)

85.Sertraline Hydrochloride (4570)

86.Hydrochloride, Sertraline (4570)

87.Sertraline Hydrochloride (1S-cis)-Isomer (0)

88.Gen-Sertraline (4570)

89.Gen Sertraline (4570)

90.#69 OR #70 OR #71 OR #72 OR #73 OR #74 OR #75 OR #76 OR #77 OR #78 OR #79 OR #80 OR #81 OR #82 OR #83 OR #84 OR #85 OR #86 OR #87 OR #88 OR # 89 (4570)

91.Fluvoxamine [Mesh] (1790)

92.Fluvoxadura (2813)

93.Fluvoxamin AL (2813)

94.Fluvoxamin beta (2813)

95.Fluvoxamin Stada (2813)

96.Fluvoxamin-neuraxpharm (2813)

97.Fluvoxamin neuraxpharm (2813)

98.Fluvoxamin-ratiopharm (2813)

99.Fluvoxamin ratiopharm (2813)

100.ratio-Fluvoxamine (2813)

101.ratio Fluvoxamine (2813)

102.Fluvoxamina Geminis (2813)

103.Geminis, Fluvoxamina (2813)

104.Fluvoxamine Maleate (2813)

105.Fluvoxamine Maleate, (E)-Isomer (0)

106.Fluvoxamine, (Z)-Isomer (2)

107.Luvox (2814)

108.Floxyfral (2813)

109.Fevarin (2814)

110.Dumirox (2813)

111.Averin (145)

112.Novo-Fluvoxamine (2813)

113.Novo Fluvoxamine (2813)

114.Nu-Fluvoxamine (2813)

115.Nu Fluvoxamine (2813)

116.PMS-Fluvoxamine (2813)

117.PMS Fluvoxamine (2813)

118.Desiflu (2813)

119.DU-23000 (2813)

120.DU 23000 (2813)

121.DU23000 (2813)

122.#91 OR #92 OR #93 OR #94 OR #95 OR #96 OR #97 OR #98 OR #99 OR #100 OR #101 OR #102 OR #103 OR #104 OR #105 OR #106 OR #107 OR #108 OR #109 OR #110 OR #111 OR #112 OR #113 OR #114 OR #115 OR #116 OR #117 OR #118 OR #119 OR #120 OR #121 (2820)

123.#20 OR #30 OR #53 OR #61 OR #68 OR #90 OR #122 (51420)

124.congenital disorder [Mesh] (1115922)

125.Abnormality, Congenital (576880)

126.Congenital Abnormality (576880)

127.Deformities (914428)

128.Deformity (605729)

129.Congenital Defects (901282)

130.Congenital Defect (580282)

131.Defect, Congenital (580282)

132.Defects, Congenital (582983)

133.Abnormalities, Congenital (574594)

134.Birth Defects (902424)

135.Birth Defect (577152)

136.Defect, Birth (577152)

137.Defects, Birth (585119)

138.#124 OR #125 OR #126 OR #127 OR #128 OR #129 OR #130 OR #131 OR #132 OR #133 OR #134 OR #135 OR #136 OR #137 (960841)

139.pregnant woman [Mesh] (6443)

140.Women, Pregnant (97814)

141.Pregnant Woman (104671)

142.Woman, Pregnant (104671)

143.#139 OR #140 OR #141 OR #142 (104671)

144.birth outcome (50139)

145.obstetrical outcome (56497)

146.#138 OR #143 OR #144 OR #145 (1128642)

147.#123 AND #146 (1736)

**ⅱ）Search strategy for Embase**

The following searches were conducted in Embase:

1.'serotonin uptake inhibitor'/exp (233963)

2.'5-hydroxytryptamine uptake inhibitors' (23)

3.'5 hydroxytryptamine uptake inhibitors' (23)

4.'inhibitors, 5-ht uptake' (0)

5.'inhibitors, 5 ht uptake' (0)

6.'inhibitors, 5-hydroxytryptamine uptake' (0)

7.'inhibitors, 5 hydroxytryptamine uptake' (0)

8.'reuptake inhibitors, serotonin' (93)

9.'serotonin reuptake inhibitors' (11614)

10.'uptake inhibitors, 5-ht' (1)

11.'uptake inhibitors, 5 ht' (1)

12.'uptake inhibitors, 5-hydroxytryptamine' (1)

13.'uptake inhibitors, 5 hydroxytryptamine' (0)

14.'uptake inhibitors, serotonin' (7)

15.'5-ht uptake inhibitors' (201)

16.'5 ht uptake inhibitors' (201)

17.'inhibitors, serotonin uptake' (2)

18.'selective serotonin reuptake inhibitors' (9947)

19.‘Inhibitors, Serotonin Reuptake’ (14)

20.#1 OR #2 OR #3 OR #4 OR #5 OR #6 OR #7 OR #8 OR #9 OR #10 OR #11 OR #12 OR #13 OR #14 OR #15 OR #16OR #17 OR #18 OR #19 (234768)

21.' Fluoxetine '/exp (42672)

22.‘Fluoxetin’ (162)

23.‘N-Methyl-gamma-(4-(trifluoromethyl)phenoxy)benzenepropanamine’ (1)

24.‘Lilly-110140’ (42679)

25.‘Lilly 110140’ (155)

26.‘Lilly110140’ (42672)

27.‘Sarafem’ (42674)

28.‘Fluoxetine Hydrochloride’ (42696)

29.‘Prozac’ (42729)

30.#21 OR #22 OR #23 OR #24 OR #25 OR #26 OR #27 OR #28 OR #29 (42781)

31.' Paroxetine '/exp (25772)

32.‘BRL-29060’ (25772)

33.‘BRL 29060’ (25772)

34.‘BRL29060’ (25772)

35.‘FG-7051’ (25772)

36.‘FG 7051’ (25772)

37.‘FG7051’ (25772)

38.‘Paroxetine Acetate’ (0)

39.‘Paroxetine Hydrochloride’ (25777)

40.‘Paroxetine Hydrochloride Hemihydrate’ (10)

41.‘Hemihydrate, Paroxetine Hydrochloride’ (0)

42.‘Hydrochloride Hemihydrate, Paroxetine’ (0)

43.‘Paroxetine Hydrochloride, Hemihydrate’ (10)

44.‘Hemihydrate Paroxetine Hydrochloride’ (0)

45.‘Hydrochloride, Hemihydrate Paroxetine’ (0)

46.‘Seroxat’ (25772)

47.‘Paroxetine Maleate’ (0)

48.‘Paroxetine, cis-(+)-Isomer’ (0)

49.‘Paroxetine, cis-(-)-Isomer’ (0)

50.‘Paroxetine, trans-(+)-Isomer’ (0)

51.‘Paxil’ (25788)

52.‘Aropax’ (25772)

53.‘Paroxetine Hydrochloride Anhydrous’ (0)

54.#31 OR #32 OR #33 OR #34 OR #35 OR #36 OR #37 OR #38 OR #39 OR #40 OR #41 OR #42 OR #43 OR #44 OR #45 OR #46 OR #47 OR #48 OR #49 OR #50 OR #51 OR #52 OR #53 (25795)

55.' Citalopram '/exp (20627)

56.Cytalopram (20629)

57.‘Lu-10-171’ (20629)

58.Lu10171 (20627)

59.Escitalopram (10059)

60.Lexapro (9818)

61.#55 OR #56 OR #57 OR #58 OR #59 OR #60 (26165)

62.' Escitalopram '/exp (9810)

63.Cytalopram (20629)

64.Lu-10-171 (20629)

65.Lu10171 (20627)

66.Escitalopram (10059)

67.Lexapro (9818)

68.#62 OR #63 OR #64 OR #65 OR #66 OR #67 (26165)

69.' Sertraline '/exp (23296)

70.Zoloft (23310)

71.Altruline (23296)

72.Lustral (23297)

73.Apo-Sertraline (0)

74.Apo Sertraline (0)

75.Aremis (53)

76.Besitran (13)

77.Sealdin (0)

78.Gladem (23298)

79.‘Novo-Sertraline’ (0)

80.‘Novo Sertraline’ (0)

81.‘ratio-Sertraline’ (0)

82.‘ratio Sertraline’ (0)

83.‘Rhoxal-sertraline’ (0)

84.‘Rhoxal sertraline’ (0)

85.‘Sertraline Hydrochloride’ (164)

86.‘Hydrochloride, Sertraline’ (0)

87.‘Sertraline Hydrochloride (1S-cis)-Isomer’ (0)

88.‘Gen-Sertraline’ (0)

89.‘Gen Sertraline’ (0)

90.#69 OR #70 OR #71 OR #72 OR #73 OR #74 OR #75 OR #76 OR #77 OR #78 OR #79 OR #80 OR #81 OR #82 OR #83 OR #84 OR #85 OR #86 OR #87 OR #88 OR #89 (23350)

91.' Fluvoxamine '/exp (12337)

92.‘Fluvoxadura’ (0)

93.‘Fluvoxamin AL’ (0)

94.‘Fluvoxamin beta’ (0)

95.‘Fluvoxamin Stada’ (0)

96.‘Fluvoxamin-neuraxpharm’ (0)

97.‘Fluvoxamin neuraxpharm’ (0)

98.‘Fluvoxamin-ratiopharm’ (0)

99.‘Fluvoxamin ratiopharm’ (0)

100. ‘ratio-Fluvoxamine’ (2)

101. ‘ratio Fluvoxamine’ (2)

102. ‘Fluvoxamina Geminis’ (0)

103. ‘Geminis, Fluvoxamina’ (0)

104. ‘Fluvoxamine Maleate’ (1678)

105. ‘Fluvoxamine Maleate, (E)-Isomer’ (0)

106. ‘Fluvoxamine, (Z)-Isomer’ (0)

107. ‘Luvox’ (1674)

108. ‘Floxyfral’ (1645)

109. ‘Fevarin’ (1659)

110. ‘Dumirox’ (12337)

111. ‘averin’ (167)

112. ‘Novo-Fluvoxamine’ (0)

113. ‘Novo Fluvoxamine’ (0)

114. ‘Nu-Fluvoxamine’ (0)

115. ‘Nu Fluvoxamine’ (0)

116. ‘PMS-Fluvoxamine’ (0)

117. ‘PMS Fluvoxamine’ (0)

118. ‘Desiflu’ (0)

119. ‘DU-23000’ (12337)

120. ‘DU 23000’ (12337)

121. ‘DU23000’ (12337)

122. #91 OR #92 OR #93 OR #94 OR #95 OR #96 OR #97 OR #98 OR #99 OR #100 OR #101 OR #102 OR #103 OR #104 OR #105 OR #106 OR #107 OR #108 OR #109 OR #110 OR #111 OR #112 OR #113 OR #114 OR #115 OR #116 OR #117 OR #118 OR #119 OR #120 OR #121 (13293)

123. #20 OR #30 OR #53 OR #61 OR #68 OR #90 OR #122 (23156)

124. ' congenital disorder '/exp (1305258)

125. ‘Abnormality, Congenital’ (17)

126. ‘Congenital Abnormality’ (1305822)

127. Deformities (31786)

128. Deformity (60433)

129. ‘Congenital Defects’ (4313)

130. ‘Congenital Defect’ (1305701)

131. ‘Defect, Congenital’ (1305268)

132. ‘Defects, Congenital’ (5147)

133. ‘Abnormalities, Congenital’ (159)

134. ‘Birth Defects’ (20613)

135. ‘Birth Defect’ (813508)

136. ‘Defect, Birth’ (812576)

137. ‘Defects, Birth’ (25)

138. #124 OR #125 OR #126 OR #127 OR #128 OR #129 OR #130 OR #131 OR #132 OR #133 OR #134 OR #135 OR #136 OR #137 (1358036)

139. ' pregnant woman '/exp (58273)

140. ‘Women, Pregnant’ (653)

141. ‘Pregnant Woman’ (65621)

142. ‘Woman, Pregnant’ (89)

143. #139 OR #140 OR #141 OR #142 (66146)

144. ‘birth outcome’ (46682)

145. ‘obstetrical outcome’ (342)

146. #138 OR #143 OR #144 OR #145 (1450752)

147. #123 AND #146 (7579)

**ⅲ) Search strategy for Web of Science**

The following searches were conducted in Web of Science:

1.TS=(Serotonin Uptake Inhibitors) (24018)

2.TS=(5-Hydroxytryptamine Uptake Inhibitors) (3085)

3.TS=(5 Hydroxytryptamine Uptake Inhibitors) (3094)

4.TS=(Inhibitors, 5-HT Uptake) (4919)

5.TS=(Inhibitors, 5 HT Uptake) (5044)

6.TS=(Inhibitors, 5-Hydroxytryptamine Uptake) (3085)

7.TS=(Inhibitors, 5 Hydroxytryptamine Uptake) (3094)

8.TS=(Inhibitors, Serotonin Reuptake) (26685)

9.TS=(Reuptake Inhibitors, Serotonin) (26685)

10.TS=(Serotonin Reuptake Inhibitors) (26685)

11.TS=(Uptake Inhibitors, 5-HT) (4919)

12.TS=(Uptake Inhibitors, 5 HT) (5044)

13.TS=(Uptake Inhibitors, 5-Hydroxytryptamine) (3085)

14.TS=(Uptake Inhibitors, 5 Hydroxytryptamine) (3094)

15.TS=(Uptake Inhibitors, Serotonin) (24018)

16.TS=(5-HT Uptake Inhibitors) (4919)

17.TS=(5 HT Uptake Inhibitors) (5044)

18.TS=(Inhibitors, Serotonin Uptake) (24018)

19.TS=(Selective Serotonin Reuptake Inhibitors) (17192)

20.#1 OR #2 OR #3 OR #4 OR #5 OR #6 OR #7 OR #8 OR #9 OR #10 OR #11 OR #12 OR #13 OR #14 OR #15 OR #16OR #17 OR #18 (39799)

21.TS=(Fluoxetine) (24038)

22.TS=(FluoxetineN-Methyl-gamma-(4-(trifluoromethyl)phenoxy)benzenepropanamine) (2)

23.TS=(Lilly-110140) (98)

24.TS=(Lilly 110140) (99)

25.TS=(Lilly110140) (0)

26.TS=(Sarafem) (8)

27.TS=(Fluoxetine Hydrochloride) (1469)

28.TS=(Prozac) (848)

29.TS=(Lilly-110140) (98)

30.#21 OR #22 OR #23 OR #24 OR #25 OR #26 OR #27 OR #28 OR #29(24293)

31.TS=(Paroxetine) (11201)

32.TS=(BRL-29060) (0)

33.TS=(BRL 29060) (0)

34.TS=(BRL29060) (0)

35.TS=(FG-7051) (3)

36.TS=(FG 7051) (3)

37.TS=(FG7051) (3)

38.TS=(Paroxetine Acetate) (83)

39.TS=(Paroxetine Hydrochloride) (817)

40.TS=(Paroxetine Hydrochloride Hemihydrate) (18)

41.TS=(Hemihydrate, Paroxetine Hydrochloride) (18)

42.TS=(Hydrochloride Hemihydrate, Paroxetine) (18)

43.TS=(Paroxetine Hydrochloride, Hemihydrate) (18)

44.TS=(Hemihydrate Paroxetine Hydrochloride) (18)

45.TS=(Hydrochloride, Hemihydrate Paroxetine) (18)

46.TS=(Seroxat) (48)

47.TS=(Paroxetine Maleate) (42)

48.TS=(Paroxetine, cis-(+)-Isomer) (9)

49.TS=(Paroxetine, cis-(-)-Isomer) (9)

50.TS=(Paroxetine, trans-(+)-Isomer) (9)

51.TS=(Paxil) (175)

52.TS=(Aropax) (3)

53.TS=(Paroxetine Hydrochloride Anhydrous) (6)

54.#31 OR #32 OR #33 OR #34 OR #35 OR #36 OR #37 OR #38 OR #39 OR #40 OR #41 OR #42 OR #43 OR #44 OR #45 OR #46 OR #47 OR #48 OR #49 OR #50 OR #51 OR #52 OR #53 (11260)

55.TS=(Citalopram) (9956)

56.TS=(Cytalopram) (9)

57.TS=(Lu-10-171) (29)

58.TS=(Lu10171) (0)

59.TS=(Escitalopram) (4090)

60.TS=(Lexapro) (77)

61.#55 OR #56 OR #57 OR #58 OR #59 OR #60 (11972)

62.TS=(Escitalopram) (4090)

63.TS=(Cytalopram) (9)

64.TS=(Lu-10-171) (29)

65.TS=(Lu10171) (0)

66.TS=(Escitalopram) (4090)

67.TS=(Lexapro)(77)

68.#62 OR #63 OR #64 OR #65 OR #66 OR #67 (4149)

69.TS=(Sertraline) (8437)

70.TS=(Zoloft) (194)

71.TS=(Altruline) (0)

72.TS=(Lustral) (14)

73.TS=(Apo-Sertraline) (0)

74.TS=(Apo Sertraline) (1)

75.TS=(Aremis) (1476)

76.TS=(Besitran) (1)

77.TS=(Sealdin) (0)

78.TS=(Gladem) (0)

79.TS=(Novo-Sertraline) (0)

80.TS=(Novo Sertraline) (9)

81.TS=(ratio-Sertraline) (0)

82.TS=(ratio Sertraline) (543)

83.TS=(Rhoxal-sertraline) (0)

84.TS=(Rhoxal sertraline) (0)

85.TS=(Sertraline Hydrochloride) (610)

86.TS=(Hydrochloride, Sertraline) (610)

87.TS=(Sertraline Hydrochloride (1S-cis)-Isomer) (1)

88.TS=(Gen-Sertraline) (0)

89.TS=(Gen Sertraline) (0)

90.#69 OR #70 OR #71 OR #72 OR #73 OR #74 OR #75 OR #76 OR #77 OR #78 OR #79 OR #80 OR #81 OR #82 OR #83 OR #84 OR #85 OR #86 OR #87 OR #88 OR #89 (8489)

91.TS=(Fluvoxamine) (5067)

92.TS=(Fluvoxadura) (0)

93.TS=(Fluvoxamin AL) (0)

94.TS=(Fluvoxamin beta) (0)

95.TS=(Fluvoxamin Stada) (0)

96.TS=(Fluvoxamin-neuraxpharm) (0)

97.TS=(Fluvoxamin neuraxpharm) (0)

98.TS=(Fluvoxamin-ratiopharm) (0)

99.TS=(Fluvoxamin ratiopharm) (0)

100. TS=(ratio-Fluvoxamine) (1)

101. TS=(ratio Fluvoxamine) (359)

102. TS=(Fluvoxamina Geminis) (0)

103. TS=(Geminis, Fluvoxamina) (0)

104. TS=(Fluvoxamine Maleate) (134)

105. TS=(Fluvoxamine Maleate, (E)-Isomer) (0)

106. TS=(Fluvoxamine, (Z)-Isomer) (5)

107. TS=(Luvox) (25)

108. TS=(Floxyfral) (2)

109. TS=(Fevarin) (26)

110. TS=(Dumirox) (3)

111. TS=(averin) (24)

112. TS=(Novo-Fluvoxamine) (0)

113. TS=(Novo Fluvoxamine) (0)

114. TS=(Nu-Fluvoxamine) (0)

115. TS=(Nu Fluvoxamine) (0)

116. TS=(PMS-Fluvoxamine) (0)

117. TS=(PMS Fluvoxamine) (18)

118. TS=(Desiflu) (0)

119. TS=(DU-23000) (2)

120. TS=(DU 23000) (28)

121. TS=(DU23000) (0)

122. #91 OR #92 OR #93 OR #94 OR #95 OR #96 OR #97 OR #98 OR #99 OR

#100 OR #101 OR #102 OR #103 OR #104 OR #105 OR #106 OR #107 OR #108 OR #109 OR #110 OR #111 OR #112 OR #113 OR #114 OR #115 OR #116 OR #117 OR #118 OR #119 OR #120 OR #121 (5098)

123. #20 OR #30 OR #53 OR #61 OR #68 OR #90 OR #122 (67154)

124. TS=(congenital disorder) (210779)

125. TS=(Abnormality, Congenital) (171920)

126. TS=(Congenital Abnormality) (171920)

127. TS=(Deformities) (91973)

128. TS=(Deformity) (91973)

129. TS=(Congenital Defects) (144014)

130. TS=(Congenital Defect) (144014)

131. TS=(Defect, Congenital) (144014)

132. TS=(Defects, Congenital) (144014)

133. TS=(Abnormalities, Congenital) (171920)

134. TS=(Birth Defects) (37122)

135. TS=(Birth Defect) (37122)

136. TS=(Defect, Birth) (37122)

137. TS=(Defects, Birth) (37122)

138. #124 OR #125 OR #126 OR #127 OR #128 OR #129 OR #130 OR #131 OR

#132 OR #133 OR #134 OR #135 OR #136 OR #137 (348942)

139. TS=(pregnant woman) (138728)

140. TS=(Women, Pregnant) (138728)

141. TS=(Pregnant Woman) (138728)

142. TS=(Woman, Pregnant) (138728)

143. #139 OR #140 OR #141 OR #142 (138728)

144. TS=(birth outcome) (94623)

145. TS=(obstetrical outcome) (6851)

146. #138 OR #143 OR #144 OR #145(556192)

147. #123 AND #146 (1076)

**ⅳ) Search strategy for Cochrane Library**

The following searches were conducted in Cochrane Library:

1. MeSH descriptor: [Serotonin Uptake Inhibitors] explode all trees (2760)

2. Inhibitors, 5-Hydroxytryptamine Uptake OR Inhibitors, 5 Hydroxytryptamine

Uptake or Inhibitors, Serotonin Reuptake OR Inhibitors, Serotonin Reuptake OR

Serotonin Reuptake Inhibitors (Word variations have been searched) (3095)

3. Uptake Inhibitors, 5-HT OR Uptake Inhibitors, 5 HT OR Uptake Inhibitors,

5-Hydroxytryptamine OR Uptake Inhibitors, 5 Hydroxytryptamine OR Uptake

Inhibitors, Serotonin (Word variations have been searched) (4295)

4. 5-HT Uptake Inhibitors OR 5-HT Uptake Inhibitors OR Inhibitors, Serotonin

Uptake OR Selective Serotonin Reuptake Inhibitors (Word variations have been

searched) (5096)

5. #1 OR #2 OR #3 OR #4 (5396)

6. MeSH descriptor: [Fluoxetine] explode all trees (1278)

7. Fluoxetine Hydrochloride OR "Prozac" (Word variations have been searched (278)

8. Lilly-110140 OR Fluoxetine N-Methyl-gamma- (4- (trifluoromethyl) phenoxy)

benzenepropanamine OR Lilly 110140 OR Lilly110140 OR Sarafem (Word

variations have been searched) (7)

9. #6 OR #7 OR #8 (1441)

10. MeSH descriptor: [Paroxetine] explode all trees (845)

11. BRL-29060 OR BRL 29060 OR BRL29060 OR FG-7051 OR FG 7051 (Word

variations have been searched) (6)

12. Hydrochloride Hemihydrate, Paroxetine OR Paroxetine Hydrochloride,

Hemihydrate OR Hemihydrate Paroxetine Hydrochloride OR Hydrochloride,

Hemihydrate Paroxetine OR Seroxat (Word variations have been searched) (20)

13. Paroxetine Maleate or Aropax OR Paroxetine Hydrochloride Anhydrousr OR

Paxil (Word variations have been searched) (49)

14. FG7051 OR Paroxetine Acetate OR Paroxetine Hydrochloride OR Paroxetine

Hydrochloride Hemihydrate OR Paroxetine Hydrochloride Hemihydrate (Word

variations have been searched) (176)

15. #10 OR #11 OR #12 OR #13 OR 14(962)

16. MeSH descriptor: [Citalopram] explode all trees (1013)

17. Cytalopram OR Lu10171 OR Lu-10-171 OR Escitalopram OR Lexapro (Word

variations have been searched) (1216)

18. #16 OR #17 (1776)

19. MeSH descriptor: [Sertraline] explode all trees (758)

20. Zoloft OR Altruline OR Lustral OR Apo Sertraline OR Apo-Sertraline (Word

variations have been searched) (43)

21. Aremis OR Besitran OR Sealdin OR Gladem OR Novo-Sertraline (Word

variations have been searched) (2)

22. Novo Sertraline OR ratio-Sertraline OR ratio Sertraline OR Rhoxal-sertraline OR

Rhoxal sertraline (Word variations have been searched) (338)

23. Sertraline Hydrochloride OR Hydrochloride, Sertraline OR Sertraline

Hydrochloride (1S-cis) -Isomer OR Gen Sertraline OR Gen-Sertraline (Word

variations have been searched) (170)

24. #19 OR #20 OR #21 OR #22 OR #23 (1070)

25. MeSH descriptor: [Fluvoxamine] explode all trees (376)

26. PMS Fluvoxamine OR Desiflu OR DU-23000 OR DU 23000 OR DU23000

(Word variations have been searched) (11)

27. Novo-Fluvoxamine OR Novo-Fluvoxamine OR Nu-Fluvoxamine OR Nu

Fluvoxamine OR PMS-Fluvoxamine (Word variations have been searched) (3)

28. Luvox or Floxyfral OR Fevarin OR Dumirox OR Faverin (Word variations have

been searched) (21)

29. Fluvoxamin neuraxpharm OR Fluvoxamin-ratiopharm OR Fluvoxamin

ratiopharm OR ratio-Fluvoxamine OR ratio Fluvoxamine (Word variations have

been searched) (183)

30. Fluvoxadura OR Fluvoxamin AL OR Fluvoxamin beta OR Fluvoxamin Stada OR

Fluvoxamin-neuraxpharm (Word variations have been searched) (6)

31. #25 OR #26 OR #27 OR #28 OR #29 OR #30 (570)

32. #31 OR 24 OR #18 OR #15 OR #9 OR #5 (7753)

33. MeSH descriptor: [Congenital Abnormalities] explode all trees (4837)

34. Birth Defects OR Birth Defect OR Defect, Birth OR Defects, Birth (Word

variations have been searched) (1216)

35. Congenital Defects OR Congenital Defect OR Defect, Congenital OR Defects,

Congenital OR Defects, Congenital (Word variations have been searched ) (1779)

36. Abnormality, Congenital OR Congenital Abnormality OR Deformities OR

Deformity OR Deformity (Word variations have been searched) (4540)

37. #33OR #34 OR 35 OR #36 (9964)

38. MeSH descriptor: [Pregnant Women] explode all trees (168)

39. Women, Pregnant OR Pregnant Woman OR Woman, Pregnant (Word variations

have been searched) (11260)

40. #38 OR #39 (11260)

41. birth outcome (5926)

42. obstetrical outcome (5846)

43. #40 OR #37 OR #41 OR #42 (29652)

44. #32 AND #43 (265)
