# Supplementary material for: Selective serotonin reuptake inhibitor use during early pregnancy and congenital malformations: a systematic review and meta-analysis of cohort studies of more than 9 million births
Source: BMC Med. 2018 Nov 12;16:205. doi: 10.1186/s12916-018-1193-5 (PMC6231277; doi:10.1186/s12916-018-1193-5)
Supplement: Supplementary file 3 — Table S1. EUBOCAT Guide 1.3, ICD-10, and ICD-9 codes used to identify and define congenital malformations. Table S2. Characteristics of cohort studies of selective serotonin reuptake inhibitors (SSRIs) use in first-trimester and congenital malformations. Table S3. Risk of bias of included reports from cohort studies as assessed with the Newcastle-Ottawa scale. Table S4. Exposure to selective serotonin reuptake inhibitors (SSRIs) during the first trimester of pregnancy and risk of congenital malformations in infants: results of meta-analyses. Table S5. Exposure to citalopram during the first trimester of pregnancy and risk of congenital malformations in infants: results of meta-analyses. Table S6. Exposure to fluoxetine during the first trimester of pregnancy and risk of congenital malformations in infants: results of meta-analyses. Table S7. Exposure to paroxetine during the first trimester of pregnancy and risk of congenital malformations in infants: results of meta-analyses. Table S8. Exposure to sertraline during the first trimester of pregnancy and risk of congenital malformations in infants: results of meta-analyses. Table S9. Exposure to escitalopram during the first trimester of pregnancy and risk of congenital malformations in infants: results of meta-analyses. Table S10. Exposure to fluvoxamine during the first trimester of pregnancy and risk of congenital malformations in infants: results of meta-analyses. Table S11. Subgroup analysis of selective serotonin reuptake inhibitors (SSRIs) and risk of congenital malformations in infants: results of meta-analyses. Table S12. Subgroup analysis of citalopram and risk of congenital malformations in infants: results of meta-analyses. Table S13. Subgroup analysis of fluoxetine and risk of congenital malformations in infants: results of meta-analyses. Table S14. Subgroup analysis of paroxetine and risk of congenital malformations in infants: results of meta-analyses. Table S15. Subgroup analysis of sertraline and risk [file 12916_2018_1193_MOESM3_ESM.doc]

**Additional file 3**

**Table S1.** EUBOCAT Guide 1.3, ICD-10 and ICD-9 codes used to identify and define congenital malformations.

**Table S2.** Characteristics of cohort studies of SSRIs use in first-trimester and congenital malformations.

**Table S3.** Risk of bias of included reports from cohort studies as assessed with the Newcastle-Ottawa Scale.

**Table S4.** Subgroup analysis of SSRIs and risk of congenital malformations in infants: results of meta-analyses.

**Table S5.** Subgroup analysis of citalopram and risk of congenital malformations in infants: results of meta-analyses.

**Table S6.** Subgroup analysis of fluoxetine and risk of congenital malformations in infants: results of meta-analyses.

**Table S7.** Subgroup analysis of paroxetine and risk of congenital malformations in infants: results of meta-analyses.

**Table S8.** Subgroup analysis of sertraline and risk of congenital malformations in infants: results of meta-analyses.

**Table S9.** Subgroup analysis of escitalopram and risk of congenital malformations in infants: results of meta-analyses.

**Table S10.** Subgroup analysis of fluvoxamine and risk of congenital malformations in infants: results of meta-analyses.

**Table S11.** Exposure to SSRIs during the first trimester of pregnancy and risk of congenital malformations in infants: results of meta-analyses.

**Table S12.** Exposure to citalopram during the first trimester of pregnancy and risk of congenital malformations in infants: results of meta-analyses.

**Table S13.** Exposure to fluoxetine during the first trimester of pregnancy and risk of congenital malformations in infants: results of meta-analyses.

**Table S14.** Exposure to paroxetine during the first trimester of pregnancy and risk of congenital malformations in infants: results of meta-analyses.

**Table S15.** Exposure to sertraline during the first trimester of pregnancy and risk of congenital malformations in infants: results of meta-analyses.

| **Table S1. EUBOCAT Guide 1.3, ICD-10 and ICD-9 codes used to identify and define congenital malformations.** | | | |  |
| --- | --- | --- | --- | --- |
| **Congenital malformations** | **EUBOCAT Guide 1.3** | **ICD-10** codes | **ICD-9** codes | **Definition of individual studies** |
| **Major congenital anomalies** (MCAs, major birth defects, major malformation) | N/A | N/A | N/A |  |
|  |  | Berard et al.,2017 [2]: Q00-Q89 excluding minor congenital malformations (Q10, Q162, Q17-Q182, Q184-Q189, Q250, Q270, Q381, Q515, Q516, Q520-Q527, Q53, Q664-Q666, Q69, Q70, Q81-Q84, Q950-Q952, Q954, Q955, Q959);  Furu et al.,2015 [34]: Q000-Q899  Except: Q170, Q381, Q53, Q650-Q656, Q82.5, Q86. | Berard et al.,2017 [2]: 740-759 excluding minor congenital malformations (743.6, 744.1, 744.2-744.4, 744.8, 744.9, 747.0, 747.5, 750.0, 752.4, 752.5, 754.6, 755.0, 755.1, 757.2-757.6, 757.8, 757.9);  Oberlander et al.,2008 [61]: 740.0 to 759.9 excluding minor congenital malformations (743.6, 744.1, 744.2–744.4, 744.8, 744.9, 747.0, 747.5, 750.0, 752.4, 752.5, 754.6, 755.0, 755.1, 757.2-757.6, 757.8, 757.9, 758.4). | Diav-Citrin et al.,2008 [45] defined as structural abnormalities in the offspring that have serious medical, surgical or cosmetic consequences. |
| **Congenital heart defects** (CHD, congenital heart anomalies, any cardiac defects, specific heart anomalies, cardiac malformations, cardiovascular anomalies, congenital malformations of the heart, cardiovascular malformation, all major cardiovascular anomalies, congenital heart malformation, all major cardiac malformations, cardiovascular congenital defects) | **Congenital Heart Defects: al17** | **Q20-Q26** | **745, 746, 7470-7474** |  |
|  |  | Berard et al.,2017 [2]: Q20-Q22; Nordeng et al.,2012 [38]: Q20-Q28;  Kornum et al.,2010 [42]: Q200-Q206, Q208-Q209, Q210-214, Q218-219, Q220-229, Q230-239, Q240-249, Q251-259, Q260-264, Q268-269;  Furu et al.,2015 [34]: Q200-Q264, Q268, Q269. | Bulbus cordis anomalies and anomalies of cardiac septal closure: 745; Other congenital anomalies of heart: 746.  Berard et al.,2017 [2]: 745-746 Huybrechts et al.,2014 [55]: 7450-7453, 7456-7459, 7460-7463, 7465, 7467, 74680, 74682, 74684-74687, 74689, 7469, 7470-7472, 7474, 74783;  Oberlander et al.,2008 [61]: 7450-7479 excluding minor congenital malformations (7470, 7475). | Furu et al.,2015 [34]: any cardiac defects; situs anomalies and looping defects, conotruncal and major aortic arch anomalies, ventral septal defect or atrial septal defect, atrioventricular septal defects, right ventricular outflow tract obstruction defects, and left ventricular outflow tract obstruction defects; Ban et al.,2014 [72]: septal defects, right ventricular outflow tract defects, left ventricular outflow tract defects, and others; Malm et al.,2011 [39]: atrial septal defects, ventricular septal defects, right ventricular outflow tract defects, transposition off great arteries, conotruncal heart defects, and left ventricular outflow tract defects; Pedersen et al.,2009 [43]: situs anomalies and looping defects, conotruncal and major arch anomalies, septal defects, AV canal, AV septal defects, right ventricular outflow tract obstruction, left ventricular outflow tract obstruction, anomalous pulmonary venous return. |
|  |  |  |  |  |
| **Septal defects** | N/A | N/A | N/A |  |
|  |  | Kornum et al.,2010 [42]: Q210-Q214, Q218–Q219. |  | Ban et al.,2014 [72]: atrial septal defect, ventricular septal defect, and atrioventricular septal defect; Pedersen et al.,2009 [43]: ventricular septal defect and atrial septal defect. |
| **Ventricular septal defects** | **VSD: al21** | **Ventricular septal defect: Q210** | **Ventricular septal defect: 7454** | Diav-Citrin et al.,2008 [45]: defined as structural anomalies of the heart. |
| **Atrial septal defects** | **ASD: al22** | **Atrial septal defect: Q211** | **Atrial septal defect: 7455** |  |
| **Atrioventricular septal defects** | **AVSD: al23** | **Atrioventricular septal defect: Q212** | **7456** |  |
| **RVOTD** | N/A | N/A | N/A |  |
|  |  | Furu et al.,2015 [34]: Q220, Q221, Q223, Q224, Q225, Q226, Q243, Q255, Q256, Q257. | Huybrechts et al.,2014 [55]: 74601, 74602, 74609, 74683, 7473. | Malm et al.,2011 [39]: pulmonary valve stenosis, pulmonary valve atresia, and infundibular pulmonary stenosis, and excluding tetralogy of Fallot; Pedersen et al.,2009: pulmonary valve atresia or stenosis, Ebstein’s anomaly. |
| **LVOTD** | N/A | N/A  Furu et al.,2015 [34]: Q230, Q231, Q232, Q234, Q238, Q242, Q244, Q251, Q252, Q253. | N/A | Malm et al.,2011 [39]: aortic valve atresia and stenosis;  Pedersen et al.,2009 [38]: aortic valve atresia or stenosis, hypoplastic left heart. |
| **Severe CHD** | **Severe CHD: al97** | **Q200, Q203, Q204, Q212, Q213, Q220, Q224-Q226, Q230, Q234, Q251, Q262** | **74500, 74510, 7452, 7453, 7456, 7461, 7462, 74600, 7463, 7467, 7471, 74742** |  |
|  |  | Jordan et al.,2016 [31]: Q200, Q203, Q204, Q212, Q2121, Q213, Q220, Q224-226, Q230, Q234, Q251, Q262. |  |  |
| **Nervous system** | **Nervous system: al2** | **Nervous system: Q00-Q07** | **Nervous system: 740-742** |  |
|  |  | Anencephaly and similar malformations: Q00; Spina bifida: Q05; Other congenital malformations of nervous system: Q07. | Anencephalus and similar anomalies: 740;  Spina bifida: 741; Other congenital anomalies of nervous system: 742. |  |
| **Neural tube defects** | **Neural tube defect: al3** | **Q00, Q01, Q05** | **740, 741, 7420** |  |
| **Eye** | **Eye: al10** | **Eye: Q10-Q15** | **Eye: 743** |  |
| **Ear, face and neck** | **Ear, face and neck: al15** | **Ear, face and neck: Q16-Q18** | **Ear, face, and neck: 744** |  |
| **Respiratory system** | **Respiratory: al34** | **Respiratory system: Q30-Q34** | **Respiratory system: 748** |  |
| **Orofacial cleft** | **Oro-facial clefts: al101** | **Cleft lip and cleft palate: Q35-Q37** | **7490, 7491, 7492** |  |
| **Cleft palate** | **Cleft palate: al103** | **Cleft palate: Q35** | **Cleft palate: 7490** |  |
| **Cleft lip with or without cleft**  **palate** | **Cleft lip with or without cleft palate: al102** | **Q36, Q37** | **7491, 7492** |  |
| **Digestive system** | **Digestive system: al40** | **Q38-Q45, Q790** | **Digestive system: 749, 750, 751** |  |
|  |  | Cleft lip and cleft palate: Q35-Q37;  Other congenital malformations of the digestive system: Q38-Q45.  Berard et al.,2017 [2]: Q35-Q45. | Cleft palate: 749;  Other congenital anomalies of upper alimentary tract: 750;  Other [congenital anomalies](http://dictionary.sensagent.com/Congenital anomalies/en-en/) of [digestive system](http://dictionary.sensagent.com/Digestive system/en-en/): 751.  Berard et al.,2017 [2]: 749-751 |  |
| **Genital system** | **Genital: al58** | **Genital organs: Q50-Q56** | **Genital organs: 752** |  |
| **Hypospadias** | **Hypospadias: al59** | **Hypospadias: Q54** | **Hypospadias: 75261** |  |
| **Urinary system** | **Urinary: al52** | **Urinary system: Q60-Q64** | **Urinary system: 753** |  |
| **Cystic kidney disease** | N/A | **Cystic kidney disease: Q619** | **Cystic kidney disease: 7531** |  |
| **Musculoskeletal system** | N/A | **Congenital malformations and deformations of the musculoskeletal system: Q65-Q79** | **754, 755, 756** |  |
|  |  |  | Certain congenital musculoskeletal deformities: 754; Other congenital anomalies of limbs: 755; Other congenital musculoskeletal anomalies: 756. |  |
| **Limb** | **Limb: al61** | **Q65-Q74** | **7543-7548, 755** |  |
|  |  |  | Other congenital anomalies of limbs: 755 |  |
| **Limb reduction** | **Limb reduction: al62** | **Q71-Q73** | **7552-7554, 7556** |  |
| **Clubfoot** | **Club foot-talipes equinovarus: al66** | **Q66** | **7545** |  |
| **Craniosynostosis** | **Craniosynostosis: al75** | **Craniosynostosis: Q75** | **Craniosynostosis: 7560** |  |
| **Abdominal wall defects** | **Abdominal wall defects: al49** | **Q792, Q793, Q795** | **Congenital anomalies of abdominal wall: 7567** |  |
| **Omphalocele** | **Omphalocele: al51** | **Omphalocele: Q792** | **7567** |  |
| **Gastroschisis** | **Gastroschisis: al50** | **Gastroschisis: Q793** | **75671** |  |
| Abbreviations: EUBOCAT, European Surveillance of Congenital Anomalies; ICD-9, International Classification of Diseases, Ninth Revision; ICD-10, International Classification of Diseases, Tenth Revision; LVOTD, Left ventricular outflow tract defects; N/A, Not available; RVOTD, Right ventricular outflow tract defects. | | | | |

| **Table S2. Characteristics of cohort studies of SSRIs use in first-trimester and congenital malformations.** | | | | | | | |
| --- | --- | --- | --- | --- | --- | --- | --- |
| **Author, year (Location)** | **Study period** | **Data Sources** | **Exposure** | **Outcomes (No. of cases),  No. of cohort** | **Outcome definition** | **Types of birth** | **Adjusted factors** |
| Berard 2017, Canada (North America) *[2] | 1998-2009 | The Quebec Pregnancy Cohort (QPC), medical service database, the Quebec Public Prescription Drug Insurance Database, the Hospitalisation Archive Database, the Quebec Statistics Database | SSRIs, Fluoxetine, Citalopram, Paroxetine, Sertraline, Fluvoxamine | Major congenital malformations (1929), 18487 | ICD-10, ICD-9 codes, and  EUBOCAT | Live-born singleton | Maternal age, welfare status, diabetes, hypertension, asthma and other medication uses including benzodiazepines as well as healthcare usage in the year prior and during the first trimester. |
| Nishigori 2017, Japan (Other regions) [30] | 2011-2016 | The Japan Environment and Children’s Study (JECS) | SSRIs | Urogenital abnormality (1269), Abdominal abnormality (1214), Upper limb abnormality (254), 95994 | Not reported | Not reported | Age, marital status, body mass index (kg/m^2^), smoking and drinking habits, smoking habits of partner, infertility treatments, diabetes or gestational diabetes, hypertension, anti-depressant drug except the SSRI, other psychiatric drugs, hypoglycemic tablet, anti-hypertensive drug and folic acid. |
| Pedersen 2016, United Kingdom (Europe) [32] | 1990-2011 | The Health Improvement Network | SSRIs | Congenital heart anomalies (1457), 209135 | Not reported | Live singleton births | Age, level of deprivation, smoking status, known history of alcohol and/or drug use, receipt of prescription for antipsychotics and/or anxiolytics, and known history of diabetes. |
| Jordan 2016, Wales, Norway, Funen, Denmark (Europe)† [31] | 2000-2010 | Denmark’s Medical Birth registry, Danish national Prescription and Patient registers, Statistics Denmark and the Funen, Denmark (Odense) EUROCAT register, Norway’s Medical Birth Registry, Wales’ health and social care | SSRIs, Fluoxetine, Citalopram, Paroxetine, Sertraline, Escitalopram | CHD (4624), VSD (2270), ASD (1084), Severe CHD (899), Neural Tube Defects (467), Abdominal wall defects (290), Limb reduction (260), Upper limb reduction (198), Clubfoot (763), Eye (317), Ear, face and neck (121), Urinary (1546), Omphalocele (95), Oro-facial clefts (883), Cleft palate (578), Cleft lip with or without palate (305), Hypospadias (1177), Gastroschisis (170), Craniosynostosis (119), 519117 | ICD-10 codes,  and  EUBOCAT Guide 1.3 | Live birth, stillbirth, late foetal death after 20 weeks, or termination of pregnancy for foetal anomaly (TOPFA) recorded in the EUROCAT register | Not reported |
| Berard 2015, Canada (North America)* [64] | 1998-2010 | The Quebec Pregnancy Cohort (QPC), medical service database, the Quebec Public Prescription Drug Insurance Database, the Hospitalisation Archive Database,the Quebec Statistics Database | SSRIs, Sertraline | Cardiac malformations (405), 18493 | ICD-10, ICD-9 codes, and  EUBOCAT | Live-born singleton | Maternal age, welfare status, diabetes, hypertension, asthma, and other medication use. |
| Furu 2015, Denmark, Finland, Iceland, Norway, Sweden (Europe) [34] | 1996-2010 | Nationwide health registers | SSRIs, Fluoxetine, Citalopram, Paroxetine, Sertraline, Escitalopram, Fluvoxamine | Major birth defects (72731), Any cardiac defects (27309), Atrial and ventricular septal defect (17879), Atrioventricular septal defect (1095), Right ventricular outflow tract obstruction (2778), Left ventricular outflow tract obstruction (2641), Cystic kidneys (849), Hypospadias (4605), Limb reduction (1048), Clubfoot (3251), Craniosynostosis (1233), Omphalocele (280), Gastroschisis (424), 2303647 | ICD-10 and  *ICD-9-CM* codes | Live singleton infants | Maternal age, year of birth, birth order, smoking, maternal diabetes, country, and use of other prescribed drugs. |
| Malm 2015, Finland (Europe)* [33] | 1996-2010 | The Drug Reimbursement Register, The Hospital Discharge Register, The Medical Birth Register, The Register of Congenital Malformations | SSRIs | MCAs (7069), 845345 | ICD-9 code, and  EUBOCAT | Singleton live births | Sex, birth period, maternal age at delivery, place of residence, marital status, parity, smoking, socioeconomic status, purchase of anxiolytics, sedative-hypnotics, or antiepileptic drugs, prepregnancy diabetes, and other chronic diseases. |
| Ban 2014, United Kingdom (Europe)† [72] | 1990-2009 | The Health Improvement Network (THIN) | SSRIs, Fluoxetine, Citalopram, Paroxetine, Sertraline, Escitalopram | MCAs (8935), Specific heart anomalies (2512), Septal defect, ASD, VSD, Right ventricular outflow tract obstruction, Left ventricular outflow tract obstruction, Nervous system (480), Eye (319), Digestive system (324), Respiratory system (213), Genital system (1314), Urinary system (838), Musculoskeletal system (451), Limb (1783), Orofacial cleft (449), Abdominal wall (72), 349127 | ICD-10 codes and EUBOCAT | Singleton live births | Maternal age at the end of pregnancy, year of childbirth, Townsend deprivation quintile, maternal smoking history, body mass index before pregnancy, and maternal diabetes, hypertension, asthma, and epilepsy in the year before conception or during pregnancy. |
| Huybrechts 2014, U.S. states, Washington, D.C. (North America)† [55] | 2000-2007 | The Medicaid Analytic eXtract | SSRIs, Fluoxetine, Paroxetine, Sertraline | Cardiac malformations (6819), VSD (3413), Right ventricular outflow tract obstruction (1106), 949504 | ICD-9 codes | Liveborn infants | Sociodemographic information (year of delivery, state of residence, age, race, and parity), multiple gestation, chronic maternal illness (hypertension, diabetes, epilepsy, and renal disease), use of suspected teratogenic medications, use of other psychotropic medications, use of antidiabetic and antihypertensive medications, and the number of distinct prescription drugs used, proxies for depression severity (number of depression diagnoses received as an outpatient and as an inpatient) and other indications for antidepressant use restricted to women with depression. |
| Knudsen 2014, Danmark (Europe) [42] | 1995-2008 | The Danish Medical Birth Registry, The Danish National Hospital Register, The Danish National Prescription Registry, The Danish EUROCAT Register | SSRIs | CHD (546), ASD (97), VSD (333), Severe CHD (129), 72280 | ICD-10, ICD-9 codes, and  EUBOCAT | Live births | Maternal age, year of conception, use of antiepileptics and/or insulin during first trimester. |
| Margulis 2013, United Kingdom (Europe) [36] | 1996-2010 | The Clinical Practice Research Datalink (CPRD), The Mother–Baby link | SSRIs | Cardiac malformations (915), Septal defects, 149464 | Not reported | Live births | Year of delivery, maternal age at delivery, prepregnancy marital status, index of multiple deprivation at the practice level, family history of congenital malformations, prepregnancy body mass, prepregnancy diabetes, cigarette smoking, alcohol intake, diagnosis of depression in baseline year, diagnosis of other mental conditions in baseline year or contact with or referral to a psychiatrist, number of health care encounters in baseline year and number of non-antidepressant drugs prescribed in baseline year. |
| Vasilakis-Scaramozza 2013, United Kingdom (North America) [58] | 1991-2002 | United Kingdom’s General Practice Research Database (GPRD) | SSRIs | Cardiovascular anomalies (35), Central nervous system anomalies (4), Genital anomalies (72), Urinary anomalies (7), 9893 | ICD-9 codes and Center for Disease Control and Prevention guidelines | Singletons live births, stillbirths, and therapeutic abortions | Prepregnancy body mass index, maternal age, cigarette smoking status, history of diabetes mellitus, insulin use, exposure to a teratogen during the first trimester, history of infertility, and premature delivery. |
| Jimenez-Solem 2012, Danmark (Europe)† [37] | 1997-2009 | The Danish Medical Birth Registry, the Danish National Hospital Register, the Register of Medicinal Product Statistics | SSRIs, Fluoxetine, Citalopram, Paroxetine, Sertraline, Escitalopram | Major malformation (29911), Congenital malformation of heart (7832), ASD (2524), VSD (2824), Septal defect (4875), Nervous system, Neural tube defect, Eye, Ear, face, and neck, Digestive system (1558), External genital organs (2523), Internal urinary system (2344), Limbs (11838), Respiratory system, Musculoskeletal system, Oro-facial clefts, Abdominal wall defects, 848786 | *EUBOCAT Guide 1.3* | Live births | Mother's age, parity, income, education, smoking, and year of conception. |
| Nordeng 2012, Norway (Europe) [38] | 1999-2009 | The Norwegian Mother and Child Cohort, the Medical Birth Registry of Norway (MBRN) | SSRIs, Fluoxetine, Paroxetine, Sertraline | Major malformation (1562), Cardiovascular malformation (547), ASD/VSD, 63395 | ICD-10 codes  and  the International Clearinghouse for Birth Defects definition | Live birth, fetal death, or induced abortion | Maternal depression, maternal age at delivery, parity, and use of psychotropic drugs during pregnancy (additionally adjusted pregnancy BMI for major malformation). |
| Klieger-Grossmann 2012, Canada (North America) [59] | Not reported | The Swiss Teratogen Information Service, the Florence Teratogen Information Service | Escitalopram | Major malformation (5), 425 | Not reported | Live births, spontaneous abortions, therapeutic abortions, and stillbirths | Not reported |
| Colvin 2011, Western Australia, (Other regions) [40] | 2002-2005 | Western Australia Data Linkage System (WADLS): Hospital Morbidity Data System, the Midwives’ Notification System, the Registry of Births and Deaths, and the Birth Defects Registry; and the national Pharmaceutical  Benefits Scheme (PBS) | SSRIs, Fluoxetine, Citalopram, Paroxetine, Sertraline | Major birth defect (3949), Cardiovascular anomalies (691), Bulbus cordis anomalies of cardiac septal closure (382), VSD (218), Ostium secundum type atrial septal defect (97), Nervous system (296), Eye (74), Ear, face, and neck (232), Congenital anomaly of genital organs (851), Urinary system (531), Respiratory system (89), Musculoskeletal defect (1076), Urogenital defect (1350), Other congenital anomalies of limbs (121), Gastrointestinal defect (549), Cystic kidney disease (67), 123405 | ICD-9 codes | Singletons or stillbirths | Not reported |
| Malm 2011, Finland (Europe) [34] | 1996-2006 | The Medical Birth Register, the Register of Congenital Malformations, the Drug Reimbursement Register | SSRIs, Fluoxetine, Citalopram, Paroxetine, Sertraline, Escitalopram, Fluvoxamine | MCAs (22608), Major cardiovascular anomalies (8253), ASD (1297), VSD (5550), Right ventricular outflow tract defects (423), Left ventricular outflow tract defects (476), Neural tube defect, Central nervous system, Respiratory tract, Musculoskeletal system, Digestive system, Cleft lip with or without cleft palate, Cleft palate, Urogenital, Omphalocele, Craniosynostosis, 635583 | *EUBOCAT Guide 1.3* | Live births, stillbirths, fetuses from pregnancy terminations attributable to severe fetal anomaly | Age at the end of pregnancy, parity, year of pregnancy ending, smoking during pregnancy, purchase of other reimbursed psychiatric drugs during the first trimester, and maternal prepregnancy diabetes. |
| Kornum 2010, Northern Denmark (Europe) [42] | 1991-2007 | The Danish Medical Birth Registry, the National Registry of Patients | SSRIs, Fluoxetine, Citalopram, Paroxetine, Sertraline, Escitalopram | Cardiac malformations (1429), Septal heart defect, 216042 | ICD-10  and  *ICD-8* codes | Live births | Maternal smoking status, maternal age, birth order, and birth year. |
| Pedersen 2010, United Kingdom (Europe)*[41] | 1993-2008 | The Health Improvement Network | SSRIs | Congenital cardiac abnormalities | Not reported | Live singleton births | Maternal age, diabetes, calendar period, smoking and alcohol consumption. |
| Merlob 2009, Israel (Other regions) [44] | 2000-2007 | Rabin Medical Center and Schneider Children’s Medical Center of Israel affiliated with The European Network Teratology Information Services, the International Clearinghouse for Birth Defects Surveillance and Research | SSRIs, Fluoxetine, Citalopram, Paroxetine, Sertraline | Congenital heart malformation (1091), 67871 | Not reported | Only in-born deliveries | Not reported |
| Pedersen 2009, Denmark (Europe) [43] | 1996-2003 | The medical birth registry, the national register of medicinal product statistics, the fertility database, the national hospital register | SSRIs, Fluoxetine, Citalopram, Paroxetine, Sertraline | Major birth defects (15573), Major cardiac malformations (4004), Septal heart defects (2327), Cleft lip with or without cleft palate (709), Cleft palate (302), Craniosynostosis (372), 496881 | ICD-10 codes and  EUBOCAT | Singleton liveborn children | Age, calendar year, income, marriage status, tobacco smoking. |
| Einarson 2009, Canada (North America) [60] | Not reported | Teratology Information Services | SSRIs, Fluoxetine, Citalopram, Paroxetine, Sertraline, Fluvoxamine | Major malformation (44), 1856 | Not reported | Live-born infants | Not reported |
| Diav-Citrin 2008, Israel, Italy, Germany (Other regions) [45] | 1994-2005 | The Israeli Teratology Information Service | Fluoxetine | Major anomalies (46), Cardiovascular anomalies (15), 2191 | Major anomalies were defined as structural abnormalities that have serious medical, surgical or cosmetic consequences. | Live-born infants, miscarriage, stillbirth, elective termination of pregnancy | Maternal age, smoking. |
|  |  |  | Paroxetine | Major anomalies (52), Cardiovascular anomalies (15), 2191 |  |  |  |
| Einarson 2008, Canada (North America) [66] | Not reported | Teratology Information Services, and previously published cases from database studies | Paroxetine | Cardiac malformations | Not reported | Live births | Not reported |
| Oberlander 2008, Canada (North America) [61] | 1998-2001 | British Columbia registry of births, hospital separation records, the PharmaCare registry, the Medical Services Plan, PharmaNet | SSRIs, Fluoxetine, Citalopram, Paroxetine, Sertraline, Fluvoxamine | MCAs (3444), Cardiovascular congenital defects (529), ASD (81), VSD (225), 119547 | ICD-9 codes | Live births | Not available |
| Kallen 2007, Sweden (Europe) [46] | 1995-2004 | The Swedish Medical Birth Register, the Register of Congenital Malformations, the Hospital Discharge Register | SSRIs, Fluoxetine, Citalopram, Paroxetine, Sertraline, Escitalopram, Fluvoxamine | Any cardiac defect (11445), VSD and/or ASD (7213), Central nervous system malformation (5347), Neural tube defect (709), Eye malformations (1094), Severe ear malformations (2147), Cystic kidneys (424), Orofacial cleft (2386), Abdominal wall defects (319), Limb-reduction defects (673), Craniosynostosis (618), Hypospadias (3188), 880431 | ICD codes | Not reported | Year of birth, maternal age, parity, smoking, and ≥3 previous miscarriages. |
| Davis 2007, United Kingdom (North America) [56] | 1996-2000 | The HMO Research Network’s CERTs program | SSRIs, Paroxetine | Bulbus cordis anomalies and anomalies of cardiac septal closure (682), Other congenital anomalies of heart (506), Eye (649), Ear, face, and neck (211), Spina bifida (424), Other congenital anomalies of nervous system (139), Congenital anomaly of genital organs (1168), Congenital anomaly of urinary system (470), Respiratory system (257), Cleft palate and cleft lip (93), Other congenital anomalies of upper alimentary tract (424), Other congenital anomalies of digestive system (139), Certain congenital musculoskeletal deformities (1259), Other congenital musculoskeletal anomalies (681), Other congenital anomalies of limbs (839), 87407 | ICD-9 codes | Fullterm infants | Health system, maternal age, and birth season. |
| Vial 2006, France (Europe) [47] | 1994-2005 | Teratology Information Services | Paroxetine | Major malformation (22), 1166 | Not reported | Spontaneous abortion, voluntary abortion, medical abortion, and medical abortion | Not reported |
| Chambers 1996, California (North America) [57] | 1989-1995 | The California Teratogen Information Service | Fluoxetine | Major malformations (12), 482 | The presence or absence of any major anomaly, defined as a structural defect occurring in less than 4 percent of the general population that has cosmetic or functional importance. | Live-born infant, spontaneous abortion, stillbirth, ectopic pregnancy, therapeutic abortion | Not reported |
| Abbreviations: ASD, Atrial septal defects; CHD, Congenital heart defects; EUBOCAT, European Surveillance of Congenital Anomalies; ICD-8, International Classification of Diseases, Eighth Revision; ICD-9, International Classification of Diseases, Ninth Revision; ICD-9-CM codes, International Classification of Diseases, Ninth Revision, Clinical Modification; ICD-10, International Classification of Diseases, Tenth Revision; MCAs, Major congenital anomalies; SSRIs, Selective serotonin reuptake inhibitors; VSD, Ventricular septal defects.  *Studies restricted the cohort to women with a psychiatric diagnosis (usually depression and anxiety).  †Studies had two analyses: a) restricted the cohort to women with a psychiatric diagnosis (usually depression and anxiety), and b) women in the general population. | | | | | | | |

| **Table S3. Risk of bias of included reports from cohort studies as assessed with the Newcastle-Ottawa Scale*** | | | | | |
| --- | --- | --- | --- | --- | --- |
| **First author, year** | **Selection (4)** | **Comparability (2)** † | **Outcome (3)** | **Total (9)** | **Comments** |
| Berard 2017 [2] | 4 | 1 | 3 | 8 | Did not control for smoking and/or alcohol using; outcomes ascertained through record linkage |
| Nishigori 2017 [30] | 4 | 2 | 1 | 7 | Data from Outcomes ascertained through self-report questionnaire |
| Pedersen 2016 [32] | 4 | 2 | 3 | 9 | Data from one of the largest primary care databases in UK |
| Jordan 2016 [31] | 4 | 0 | 3 | 7 | Did not control for maternal age, smoking and/or alcohol using; outcomes ascertained through record linkage |
| Berard 2015 [64] | 4 | 1 | 3 | 8 | Did not control for smoking and/or alcohol using; outcomes ascertained through record linkage |
| Furu 2015 [34] | 4 | 2 | 3 | 9 | Outcomes ascertained through record linkage; outcomes ascertained through record linkage |
| Malm 2015 [33] | 4 | 2 | 3 | 9 | Outcomes ascertained through record linkage; outcomes ascertained through record linkage |
| Ban 2014 [72] | 4 | 2 | 3 | 9 | Outcomes ascertained through record linkage; outcomes ascertained through record linkage |
| Huybrecht 2014 [55] | 4 | 0‡ | 3 | 7 | Did not control for maternal age, smoking and/or alcohol using; outcomes ascertained through record linkage |
| Knudsen 2014 [35] | 4 | 1 | 3 | 8 | Did not control for smoking and/or alcohol using; outcomes ascertained through record linkage |
| Margulis 2013 [36] | 4 | 2 | 3 | 9 | Outcomes ascertained through electronic medical records |
| Vasilakis-Scaramozza 2013 [58] | 4 | 2 | 3 | 9 | Outcomes ascertained through clinical records |
| Jimenez-Solem 2012 [37] | 4 | 2 | 3 | 9 | Outcomes ascertained through record linkage |
| Nordeng 2012 [38] | 4 | 1 | 3 | 8 | Did not control for smoking and/or alcohol using |
| Klieger-Grossmann 2012 [59] | 3 | 0 | 1 | 4 | Cohort (n=5 of 452) based on Teratogen Information Service studies; did not control for maternal age, smoking and/or alcohol using; outcomes ascertained through self-report |
| Colvin 2011 [40] | 4 | 0 | 3 | 7 | Did not control for maternal age, smoking and/or alcohol using |
| Malm 2011 [39] | 4 | 2 | 3 | 9 | Outcomes ascertained through record linkage |
| Kornum 2010 [42] | 4 | 2 | 3 | 9 | Outcomes ascertained through record linkage |
| Pedersen 2010 [41] | 4 | 2 | 3 | 9 | Outcomes ascertained through record linkage |
| Merlob 2009 [44] | 4 | 0 | 3 | 7 | Did not control for maternal age, smoking and/or alcohol using |
| Pedersen 2009 [43] | 4 | 2 | 3 | 9 | Outcomes ascertained through record linkage |
| Einarson 2009 [60] | 3 | 0 | 1 | 4 | Cohort based on Teratogen Information Service studies; outcomes ascertained through self-report; did not control for maternal age, smoking and/or alcohol using |
| Oberlander 2008 [61] | 4 | 0 | 3 | 7 | Did not control for maternal age, smoking and/or alcohol using |
| Einarson 2008 [66] | 3 | 0 | 1 | 4 | Cohort based on Teratogen Information Service studies and previously published cases from database studies; did not control for maternal age, smoking and/or alcohol using; outcomes ascertained through self-report |
| Diav-Citrin 2008 [45] | 3 | 2 | 2 | 7 | Cohort based on Teratogen Information Service studies; did not control for maternal age, smoking and/or alcohol using; outcomes ascertained through self-report |
| Kallen 2007 [46] | 4 | 2 | 3 | 9 | Outcomes ascertained through record linkage |
| Davis 2007 [56] | 4 | 1 | 3 | 8 | Did not control for smoking and/or alcohol using |
| Vial 2006 [47] | 3 | 0 | 1 | 4 | Cohort (n=22 of 1166) based on Teratogen Information Service studies; did not control for maternal age, smoking and/or alcohol using; outcomes ascertained through self-report |
| Chambers 1996 [57] | 3 | 0 | 2 | 5 | Cohort (n=12 of 482) based on Teratogen Information Service studies; did not control for maternal age, smoking and/or alcohol using |
| * A study could be awarded a maximum of one star for each item except for the item Control for important factor or additional factor.  † A maximum of 2 stars could be awarded for this item. Studies that controlled for maternal age received one star, whereas studies that controlled for other important confounders such as smoking and/or alcohol using received an additional star. ‡ 1 star could be awarded for this item in the psychiatric diagnosis controlled analyses. | | | | | |

| **Table S4. Exposure to SSRIs during the first trimester of pregnancy and risk of congenital malformations in infants: results of meta-analyses.** | | | | |  |
| --- | --- | --- | --- | --- | --- |
|  | **SSRIs** | | | |  |
|  | **No. of studies** | **Summary RR (95% CI)** | I^2^, % | P-value |  |
| **Major congenital anomalies** | 9 | **1.11 (1.03 to 1.19)** | 38.4 | 0.11 |  |
| **CHD** | 18 | **1.24 (1.11 to 1.37)** | 59.0 | **0.001** |  |
| Septal defects | 6 | **1.38 (1.00 to 1.91)** | 67.4 | **0.009** |  |
| ASD | 7 | **1.83 (1.22 to 2.73)** | 72.0 | **0.002** |  |
| VSD | 8 | 1.10 (0.94 to 1.29) | 36.2 | 0.14 |  |
| RVOTD | 4 | **1.38 (1.09 to 1.75)** | 33.0 | 0.21 |  |
| LVOTD | 3 | 1.08 (0.81 to 1.44) | 0.0 | 0.89 |  |
| Severe CHD | 2 | 2.28 (0.88 to 5.94) | 78.3 | **0.03** |  |
| **Nervous system** | 7 | 1.09 (0.87 to 1.36) | 0.0 | 0.68 |  |
| Neural tube defects | 4 | **1.49 (1.05 to 2.10)** | 0.0 | 0.43 |  |
| **Eye** | 6 | 1.09 (0.85 to 1.40) | 0.0 | 0.83 |  |
| **Ear, face and neck** | 5 | 1.06 (0.64 to 1.76) | 36.4 | 0.18 |  |
| **Respiratory system** | 5 | 1.14 (0.66 to 1.99) | 48.3 | 0.10 |  |
| **Orofacial cleft** | 5 | 1.02 (0.78 to 1.34) | 0.0 | 0.65 |  |
| Cleft lip with or without cleft palate | 3 | 0.94 (0.57 to 1.55) | 0.0 | 0.37 |  |
| Cleft palate | 3 | 1.18 (0.76 to 1.81) | 0.0 | 0.42 |  |
| **Digestive system** | 5 | 1.23 (0.94 to 1.60) | 23.5 | 0.27 |  |
| **Urogenital system** | 3 | 1.30 (0.80 to 2.11) | 74.1 | **0.02** |  |
| **Urinary system** | 6 | 1.17 (0.97 to 1.42) | 0.0 | 0.83 |  |
| Cystic kidney disease | 3 | **2.96 (1.87 to 4.70)** | 0.0 | 0.81 |  |
| **Genital system** | 5 | 0.90 (0.67 to 1.21) | 47.4 | 0.11 |  |
| Hypospadias | 3 | 1.19 (0.93 to 1.51) | 0.0 | 0.88 |  |
| **Musculoskeletal system** | 5 | 0.88 (0.74 to 1.04) | 0.0 | 0.48 |  |
| Craniosynostosis | 5 | 1.05 (0.75 to 1.48) | 0.0 | 0.75 |  |
| **Limb** | 8 | 1.00 (0.82 to 1.21) | 19.6 | 0.27 |  |
| Clubfoot | 2 | **1.30 (1.06 to 1.61)** | 0.0 | 0.65 |  |
| **Abdominal wall defects** | 5 | **1.81 (1.22 to 2.68)** | 0.0 | 0.86 |  |
| Omphalocele | 3 | **1.73 (1.03 to 2.89)** | 0.0 | 0.73 |  |
| Gastroschisis | 2 | **1.89 (1.19 to 3.00)** | 0.0 | 0.56 |  |
| Abbreviations: ASD, Atrial septal defects; CHD, Congenital heart defects; CI, Confidence interval; LVOTD, Left ventricular outflow tract defects; RR, Relative risk; RVOTD, Right ventricular outflow tract defects; SSRIs, Selective serotonin reuptake inhibitors; VSD, Ventricular septal defects. | | | | | |

| **Table S5. Exposure to citalopram during the first trimester of pregnancy and risk of congenital malformations in infants: results of meta-analyses.** | | | | |
| --- | --- | --- | --- | --- |
|  | **Citalopram** | | | |
|  | **No. of studies** | **Summary RR (95% CI)** | I^2^, % | P-value |
| **Major congenital anomalies** | 8 | **1.20 (1.09 to 1.31)** | 13.4 | 0.33 |
| **CHD** | 11 | **1.24 (1.02 to 1.51)** | 52.5 | **0.02** |
| Septal defects | 4 | **1.81 (1.22 to 2.68)** | 0.0 | 0.55 |
| ASD | 4 | 1.31 (0.61 to 2.80) | 68.3 | **0.02** |
| VSD | 4 | 1.18 (0.91 to 1.52) | 0.0 | 0.71 |
| RVOTD | 2 | **1.59 (1.08 to 2.35)** | 0.0 | 0.54 |
| LVOTD | 2 | 1.50 (0.98 to 2.30) | 0.0 | 0.66 |
| Severe CHD | 1 | **2.09 (1.25 to 3.50)** | - | - |
| **Nervous system** | 4 | 1.16 (0.74 to 1.83) | 0.0 | 0.97 |
| Neural tube defects | 2 | 1.64 (0.66 to 4.08) | 56.3 | 0.13 |
| **Eye** | 4 | **2.00 (1.13 to 3.54)** | 0.0 | 0.55 |
| **Ear, face and neck** | 1 | 2.18 (0.81 to 5.88) | - | - |
| **Respiratory system** | 3 | 0.70 (0.29 to 1.68) | 0.0 | 0.69 |
| **Orofacial cleft** | 3 | 1.14 (0.64 to 2.01) | 0.0 | 0.47 |
| Cleft lip with or without cleft palate | 2 | 0.65 (0.27 to 1.57) | 0.0 | 0.93 |
| Cleft palate | 2 | 0.87 (0.40 to 1.96) | 0.0 | 0.95 |
| **Digestive system** | 4 | 1.59 (0.93 to 2.73) | 45.5 | 0.14 |
| **Urogenital system** | 2 | 0.84 (0.55 to 1.29) | 0.0 | 0.64 |
| **Urinary system** | 4 | **1.72 (1.27 to 2.33)** | 0.0 | 0.72 |
| Cystic kidney disease | - | - | - | - |
| **Genital system** | 3 | 0.98 (0.49 to 1.98) | 52.6 | 0.12 |
| Hypospadias | 2 | **1.87 (1.23 to 2.83)** | 0.0 | 0.43 |
| **Musculoskeletal system** | 4 | 1.05 (0.77 to 1.42) | 0.0 | 0.98 |
| Craniosynostosis | 3 | 1.08 (0.58 to 2.03) | 0.0 | 0.88 |
| **Limb** | 5 | 1.20 (0.94 to 1.54) | 5.6 | 0.38 |
| Clubfoot | 2 | 1.35 (0.86 to 2.14) | 24.8 | 0.25 |
| **Abdominal wall defects** | 2 | 1.41 (0.53 to 3.80) | 0.0 | 0.50 |
| Omphalocele | - | - | - | - |
| Gastroschisis | 1 | 1.56 (0.39 to 6.27) | - | - |
| Abbreviations: ASD, Atrial septal defects; CHD, Congenital heart defects; CI, Confidence interval; LVOTD, Left ventricular outflow tract defects; RR, Relative risk; RVOTD, Right ventricular outflow tract defects; VSD, Ventricular septal defects. | | | | |

| **Table S6. Exposure to fluoxetine during the first trimester of pregnancy and risk of congenital malformations in infants: results of meta-analyses.** | | | | |
| --- | --- | --- | --- | --- |
|  | **Fluoxetine** | | | |
|  | **No. of studies** | **Summary RR (95% CI)** | I^2^, % | P-value |
| **Major congenital anomalies** | 11 | **1.17 (1.07 to 1.28)** | 0.0 | 0.50 |
| **CHD** | 14 | **1.30 (1.12 to 1.53)** | 29.3 | 0.14 |
| Septal defects | 4 | **1.65 (1.02 to 2.67)** | 0.0 | 0.99 |
| ASD | 4 | 1.62 (0.90 to 2.92) | 33.7 | 0.21 |
| VSD | 5 | 1.12 (0.77 to 1.63) | 42.1 | 0.14 |
| RVOTD | 3 | **1.63 (1.11 to 2.41)** | 18.0 | 0.30 |
| LVOTD | 2 | 0.85 (0.41 to 1.78) | 0.0 | 0.56 |
| Severe CHD | 1 | 1.72 (0.85 to 3.48) | - | - |
| **Nervous system** | 3 | 1.37 (0.83 to 2.25) | 0.0 | 0.53 |
| Neural tube defects | 3 | **2.28 (1.28 to 4.06)** | 0.0 | 0.76 |
| **Eye** | 4 | 1.77 (0.91 to 3.42) | 0.0 | 0.42 |
| **Ear, face and neck** | 2 | **3.45 (1.28 to 9.29)** | 0.0 | 0.41 |
| **Respiratory system** | 3 | 1.39 (0.69 to 2.78) | 0.0 | 0.67 |
| **Orofacial cleft** | 3 | 1.03 (0.45 to 2.37) | 27.8 | 0.25 |
| Cleft lip with or without cleft palate | 2 | 0.61 (0.20 to 1.90) | 0.0 | 0.77 |
| Cleft palate | 1 | 1.03 (0.33 to 3.23) | - | - |
| **Digestive system** | 4 | 1.49 (0.83 to 2.65) | 24.9 | 0.26 |
| **Urogenital system** | 2 | 1.38 (0.87 to 2.18) | 0.0 | 0.79 |
| **Urinary system** | 3 | 1.42 (0.95 to 2.13) | 0.0 | 0.65 |
| Cystic kidney disease | - | - | - | - |
| **Genital system** | 3 | 0.73 (0.34 to 1.57) | 38.4 | 0.20 |
| Hypospadias | 2 | 0.90 (0.31 to 2.64) | 68.4 | 0.08 |
| **Musculoskeletal system** | 4 | 0.82 (0.54 to 1.22) | 0.0 | 0.73 |
| Craniosynostosis | 1 | 1.12 (0.15 to 8.26) | - | - |
| **Limb** | 5 | 1.04 (0.69 to 1.57) | 39.0 | 0.16 |
| Clubfoot | 2 | 0.98 (0.57 to 1.68) | 0.0 | 0.51 |
| **Abdominal wall defects** | 2 | 1.56 (0.58 to 4.21) | 0.0 | 0.72 |
| Omphalocele | 2 | 2.42 (0.60 to 9.82) | 28.6 | 0.24 |
| Gastroschisis | 1 | 1.21 (0.17 to 8.62) | - | - |
| Abbreviations: ASD, Atrial septal defects; CHD, Congenital heart defects; CI, Confidence interval; LVOTD, Left ventricular outflow tract defects; RR, Relative risk; RVOTD, Right ventricular outflow tract defects; VSD, Ventricular septal defects. | | | | |

| **Table S7. Exposure to paroxetine during the first trimester of pregnancy and risk of congenital malformations in infants: results of meta-analyses.** | | | | |
| --- | --- | --- | --- | --- |
|  | **Paroxetine** | | | |
|  | **No. of studies** | **Summary RR (95% CI)** | I^2^, % | P-value |
| **Major congenital anomalies** | 11 | **1.18 (1.05 to 1.32)** | 0.0 | 0.64 |
| **CHD** | 16 | **1.35 (1.19 to 1.53)** | 0.0 | 0.71 |
| Septal defects | 4 | 1.58 (0.90 to 2.78) | 0.0 | 0.68 |
| ASD | 4 | 2.06 (0.92 to 4.60) | 41.1 | 0.17 |
| VSD | 5 | 1.37 (0.90 to 2.08) | 48.2 | 0.10 |
| RVOTD | 3 | **2.15 (1.04 to 4.44)** | 67.0 | **0.049** |
| LVOTD | - | - | - | - |
| Severe CHD | 1 | 1.59 (0.51 to 4.95) | - | - |
| **Nervous system** | 2 | 0.61 (0.16 to 2.38) | 0.0 | 0.35 |
| Neural tube defects | 1 | 2.11 (0.53 to 8.43) | - | - |
| **Eye** | 3 | **2.26 (1.26 to 4.04)** | 0.0 | 0.53 |
| **Ear, face and neck** | 2 | 3.29 (0.53 to 20.30) | 41.2 | 0.19 |
| **Respiratory system** | 4 | 1.81 (0.75 to 4.39) | 0.0 | 0.51 |
| **Orofacial cleft** | 2 | 1.32 (0.23 to 7.52) | 35.9 | 0.21 |
| Cleft lip with or without cleft palate | 2 | 0.87 (0.22 to 3.49) | 0.0 | 0.96 |
| Cleft palate | 2 | **2.82 (1.26 to 6.32)** | 0.0 | 0.83 |
| **Digestive system** | 4 | 1.11 (0.54 to 2.29) | 7.4 | 0.36 |
| **Urogenital system** | 2 | 1.30 (0.80 to 2.09) | 0.0 | 0.55 |
| **Urinary system** | 3 | 1.22 (0.67 to 2.20) | 0.0 | 0.73 |
| Cystic kidney disease | 1 | **5.35 (1.31 to 21.89)** | - | - |
| **Genital system** | 4 | 1.29 (0.56 to 2.98) | 68.7 | **0.02** |
| Hypospadias | 1 | 1.52 (0.57 to 4.06) | - | - |
| **Musculoskeletal system** | 5 | 0.83 (0.54 to 1.26) | 0.0 | 0.65 |
| Craniosynostosis | 2 | 2.99 (0.74 to 12.04) | 0.0 | 0.65 |
| **Limb** | 4 | 0.87 (0.53 to 1.42) | 0.0 | 0.98 |
| Clubfoot | 1 | 0.64 (0.09 to 4.55) | - | - |
| **Abdominal wall defects** | 1 | 1.72 (0.24 to 12.29) | - | - |
| Omphalocele | 1 | 1.83 (0.25 to 13.32) | - | - |
| Gastroschisis | - | - | - | - |
| Abbreviations: ASD, Atrial septal defects; CHD, Congenital heart defects; CI, Confidence interval; LVOTD, Left ventricular outflow tract defects; RR, Relative risk; RVOTD, Right ventricular outflow tract defects; VSD, Ventricular septal defects. | | | | |

| **Table S8. Exposure to sertraline during the first trimester of pregnancy and risk of congenital malformations in infants: results of meta-analyses.** | | | | |
| --- | --- | --- | --- | --- |
|  | **Sertraline** | | | |
|  | **No. of studies** | **Summary RR (95% CI)** | I^2^, % | P-value |
| **Major congenital anomalies** | 9 | 1.10 (0.99 to 1.22) | 0.0 | 0.69 |
| **CHD** | 13 | **1.42 (1.12 to 1.80)** | 63.9 | **0.001** |
| Septal defects | 4 | **2.69 (1.76 to 4.10)** | 16.8 | 0.31 |
| ASD | 4 | **2.07 (1.26 to 3.39)** | 0.0 | 0.54 |
| VSD | 5 | 1.21 (0.65 to 2.22) | 72.6 | **0.01** |
| RVOTD | 2 | 1.18 (0.82 to 1.68) | 0.0 | 0.41 |
| LVOTD | 1 | 0.82 (0.37 to 1.83) | - | - |
| Severe CHD | 1 | 2.12 (0.88 to 5.11) | - | - |
| **Nervous system** | 4 | 1.27 (0.68 to 2.38) | 0.0 | 0.93 |
| Neural tube defects | 1 | 1.77 (0.43 to 7.25) | - | - |
| **Eye** | 2 | 1.13 (0.28 to 4.52) | 0.0 | 0.92 |
| **Ear, face and neck** | 3 | 2.79 (0.54 to 14.35) | 61.7 | 0.07 |
| **Respiratory system** | 4 | **2.65 (1.32 to 5.32)** | 0.0 | 0.45 |
| **Orofacial cleft** | 3 | 1.41 (0.63 to 3.15) | 0.0 | 0.77 |
| Cleft lip with or without cleft palate | 2 | 1.20 (0.39 to 3.73) | 0.0 | 0.83 |
| Cleft palate | 1 | 1.42 (0.35 to 5.76) | - | - |
| **Digestive system** | 4 | 1.27 (0.72 to 2.24) | 0.0 | 0.67 |
| **Urogenital system** | 2 | 1.26 (0.82 to 1.92) | 0.0 | 0.93 |
| **Urinary system** | 1 | 3.36 (0.82 to 13.77) | - | - |
| Cystic kidney disease | 4 | 1.07 (0.62 to 1.85) | 0.0 | 0.61 |
| **Genital system** | 3 | 0.87 (0.38 to 1.99) | 20.4 | 0.29 |
| Hypospadias | 1 | 1.59 (0.66 to 3.83) | - | - |
| **Musculoskeletal system** | 4 | 0.92 (0.58 to 1.44) | 0.0 | 0.59 |
| Craniosynostosis | 3 | 1.58 (0.78 to 3.17) | 0.0 | 0.64 |
| **Limb** | 4 | **1.42 (1.03 to 1.95)** | 0.0 | 0.54 |
| Clubfoot | 2 | **1.72 (1.11 to 2.65)** | 0.0 | 0.77 |
| **Abdominal wall defects** | 2 | 2.53 (0.63 to 10.24) | 0.0 | 0.36 |
| Omphalocele | - | - | - | - |
| Gastroschisis | 2 | 1.12 (0.50 to 2.52) | 0.0 | 0.44 |
| Abbreviations: ASD, Atrial septal defects; CHD, Congenital heart defects; CI, Confidence interval; LVOTD, Left ventricular outflow tract defects; RR, Relative risk; RVOTD, Right ventricular outflow tract defects; VSD, Ventricular septal defects. | | | | |

| **Table S9. Exposure to escitalopram during the first trimester of pregnancy and risk of congenital malformations in infants: results of meta-analyses.** | | | | |
| --- | --- | --- | --- | --- |
|  | **Escitalopram** | | | |
|  | **No. of studies** | **Summary RR (95% CI)** | I^2^, % | P-value |
| **Major congenital anomalies** | 5 | 0.87 (0.73 to 1.04) | 0.0 | 0.93 |
| **CHD** | 6 | 0.87 (0.69 to 1.10) | 0.0 | 0.46 |
| Septal defects | 2 | 2.15 (0.59 to 7.86) | 41.2 | 0.19 |
| ASD | 2 | 1.43 (0.79 to 2.59) | 0.0 | 0.72 |
| VSD | 2 | 1.04 (0.56 to 1.93) | 45.2 | 0.18 |
| RVOTD | - | - | - | - |
| LVOTD | 1 | 2.21 (0.31 to 15.86) | - | - |
| Severe CHD | 1 | 0.55 (0.18 to 1.70) | - | - |
| **Nervous system** | 2 | 2.35 (0.59 to 9.41) | 0.0 | 0.95 |
| Neural tube defects | 1 | 1.48 (0.55 to 3.97) | - | - |
| **Eye** | 2 | 1.27 (0.21 to 7.58) | 36.2 | 0.21 |
| **Ear, face and neck** | 1 | 2.70 (0.67 to 10.90) | - | - |
| **Respiratory system** | 1 | 2.66 (0.37 to 19.07) | - | - |
| **Orofacial cleft** | 1 | 1.61 (0.83 to 3.11) | - | - |
| Cleft lip with or without cleft palate | 1 | 1.67 (0.75 to 3.72) | - | - |
| Cleft palate | 1 | 1.05 (0.26 to 4.23) | - | - |
| **Digestive system** | - | - | - | - |
| **Urogenital system** | 1 | 0.37 (0.05 to 2.70) | - | - |
| **Urinary system** | 1 | 0.73 (0.35 to 1.53) | - | - |
| Cystic kidney disease | - | - | - | - |
| **Genital system** | 2 | 0.93 (0.23 to 3.76) | 0.0 | 0.83 |
| Hypospadias | 1 | 0.99 (0.47 to 2.08) | - | - |
| **Musculoskeletal system** | 1 | 1.36 (0.19 to 9.74) | - | - |
| Craniosynostosis | 2 | 1.33 (0.63 to 2.82) | 0.0 | 0.59 |
| **Limb** | 3 | 0.90 (0.25 to 3.23) | 58.2 | 0.09 |
| Clubfoot | 1 | **2.18 (1.16 to 4.08)** | - | - |
| **Abdominal wall defects** | 1 | **3.52 (1.56 to 7.93)** | - | - |
| Omphalocele | 1 | 1.68 (0.23 to 12.17) | - | - |
| Gastroschisis | 1 | **3.95 (1.46 to 10.68)** | - | - |
| Abbreviations: ASD, Atrial septal defects; CHD, Congenital heart defects; CI, Confidence interval; LVOTD, Left ventricular outflow tract defects; RR, Relative risk; RVOTD, Right ventricular outflow tract defects; VSD, Ventricular septal defects. | | | | |

| **Table S10. Exposure to fluvoxamine during the first trimester of pregnancy and risk of congenital malformations in infants: results of meta-analyses.** | | | | |
| --- | --- | --- | --- | --- |
|  | **Fluvoxamine** | | | |
|  | **No. of studies** | **Summary RR (95% CI)** | I^2^, % | P-value |
| **Major congenital anomalies** | 4 | 0.77 (0.49 to 1.21) | 0.0 | 0.79 |
| **Congenital heart defects** | 1 | 0.56 (0.14 to 2.25) | - | - |
| VSD | 1 | 0.42 (0.06 to 3.00) | - | - |
| **Nervous system** | 1 | 2.34 (0.58 to 9.49) | - | - |
| Neural tube defects | 1 | 2.86 (0.39 to 20.73) | - | - |
| **Digestive system** | 1 | 1.35 (0.19 to 9.64) | - | - |
| **Musculoskeletal system** | 1 | 0.79 (0.20 to 3.19) | - | - |
| Abbreviations: CI, Confidence interval; RR, Relative risk; VSD, Ventricular septal defects. | | | | |

| **Table S11. Subgroup analysis of SSRIs and risk of congenital malformations in infants: results of meta-analyses.** | | | | | | |
| --- | --- | --- | --- | --- | --- | --- |
|  | | **SSRIs** | | | | |
|  | | **No. of studies** | **Summary RR (95% CI)** | I^2^, % | ***P**** | P** |
| **Major congenital anomalies** | | 9 | **1.11 (1.03 to 1.19)** | 38.4 | 0.11 |  |
| Study quality | |  |  |  |  | 0.48 |
|  | High risk | 1 | 1.39 (0.78 to 2.49) | - | - |  |
|  | Low risk | 8 | **1.11 (1.03 to 1.19)** | 43.7 | 0.09 |  |
| Geographic location | |  |  |  |  | 0.38 |
|  | Europe | 6 | **1.13 (1.05 to 1.22)** | 41.2 | 0.13 |  |
|  | Northern America | 2 | 1.03 (0.71 to 1.50) | 43.3 | 0.18 |  |
|  | Others | 1 | 1.05 (0.87 to 1.27) | - | - |  |
| Adjustment for confounders | |  |  |  |  |  |
|  | Age |  |  |  |  | 0.27 |
|  | Yes | 6 | **1.13 (1.05 to 1.22)** | 41.2 | 0.13 |  |
|  | No | 3 | 1.01 (0.87 to 1.17) | 7.0 | 0.34 |  |
|  | Socioeconomic status |  |  |  |  | 0.36 |
|  | Yes | 3 | 1.17 (0.96 to 1.43) | 73.1 | **0.02** |  |
|  | No | 6 | **1.10 (1.05 to 1.16)** | 0.0 | 0.49 |  |
|  | Smoking or alcohol drinking |  |  |  |  | 0.27 |
|  | Yes | 5 | **1.14 (1.05 to 1.23)** | 52.8 | 0.08 |  |
|  | No | 4 | 1.01 (0.88 to 1.16) | 0.0 | 0.53 |  |
|  | Pregnancy BMI |  |  |  |  | 0.36 |
|  | Yes | 2 | 1.01 (0.88 to 1.16) | 0.0 | 0.85 |  |
|  | No | 7 | **1.13 (1.04 to 1.22)** | 44.3 | 0.10 |  |
|  | Pregnancy complications |  |  |  |  | 0.55 |
|  | Yes | 3 | **1.10 (1.04 to 1.17)** | 7.8 | 0.34 |  |
|  | No | 6 | 1.14 (0.98 to 1.32) | 49.5 | 0.08 |  |
|  | Parity |  |  |  |  | 0.15 |
|  | Yes | 4 | **1.16 (1.06 to 1.27)** | 46.8 | 0.13 |  |
|  | No | 5 | 1.03 (0.94 to 1.13) | 0.0 | 0.48 |  |
| **Congenital heart defects** | | 18 | **1.24 (1.11 to 1.37)** | 59.0 | **0.001** |  |
| Study quality | |  |  |  |  | - |
|  | High risk | - | - | - | - |  |
|  | Low risk | 18 | **1.24 (1.11 to 1.37)** | 59.0 | **0.01** |  |
| Geographic location | |  |  |  |  | 0.25 |
|  | Europe | 13 | **1.21 (1.06 to 1.38)** | 64.4 | **0.001** |  |
|  | Northern America | 3 | **1.24 (1.13 to 1.36)** | 0.0 | 0.41 |  |
|  | Others | 2 | **1.71 (1.23 to 2.37)** | 0.0 | 0.45 |  |
| Adjustment for confounders | |  |  |  |  |  |
| Age | |  |  |  |  | 0.84 |
|  | Yes | 12 | **1.23 (1.07 to 1.42)** | 65.4 | **0.001** |  |
|  | No | 6 | **1.24 (1.05 to 1.47)** | 47.1 | 0.09 |  |
| Socioeconomic status | |  |  |  |  | 0.47 |
|  | Yes | 5 | 1.30 (0.95 to 1.78) | 75.7 | **0.002** |  |
|  | No | 13 | **1.18 (1.08 to 1.29)** | 38.8 | 0.08 |  |
| Smoking or alcohol drinking | |  |  |  |  | 0.87 |
|  | Yes | 9 | **1.23 (1.05 to 1.45)** | 72.6 | **<0.001** |  |
|  | No | 9 | **1.23 (1.07 to 1.43)** | 33.7 | 0.15 |  |
| Pregnancy BMI | |  |  |  |  | 0.48 |
|  | Yes | 2 | 1.12 (0.90 to 1.38) | 0.0 | 0.70 |  |
|  | No | 16 | **1.26 (1.12 to 1.41)** | 63.3 | **<0.001** |  |
| Pregnancy complications | |  |  |  |  | 0.15 |
|  | Yes | 5 | **1.13 (1.05 to 1.22)** | 0.0 | 0.94 |  |
|  | No | 13 | **1.33 (1.14 to 1.56)** | 66.7 | **<0.001** |  |
| Parity | |  |  |  |  | 0.57 |
|  | Yes | 6 | **1.31 (1.05 to 1.63)** | 82.0 | **<0.001** |  |
|  | No | 12 | **1.19 (1.08 to 1.32)** | 19.8 | 0.25 |  |
| Abbreviations: BMI, body mass index; CI, confidence interval; RR, relative risk; SSRIs, Selective serotonin reuptake inhibitors. *P for heterogeneity within each subgroup. **P for heterogeneity between subgroups with meta-regression analysis. | | | | | | |

| **Table S12. Subgroup analysis of citalopram and risk of congenital malformations in infants: results of meta-analyses.** | | | | | | |
| --- | --- | --- | --- | --- | --- | --- |
|  | | **Citalopram** | | | | |
|  | | **No. of studies** | **Summary RR (95% CI)** | I^2^, % | ***P**** | P** |
| **Major congenital anomalies** | | 8 | **1.20 (1.09 to 1.31)** | 13.4 | 0.33 |  |
| Study quality | |  |  |  |  | 0.49 |
|  | High risk | 1 | 1.61 (0.74 to 3.51) | - | - |  |
|  | Low risk | 7 | **1.19 (1.08 to 1.31)** | 20.1 | 0.28 |  |
| Geographic location | |  |  |  |  | 0.69 |
|  | Europe | 5 | **1.18 (1.04 to 1.35)** | 45.9 | 0.12 |  |
|  | Northern America | 2 | 1.46 (0.80 to 2.68) | 0.0 | 0.70 |  |
|  | Others | 1 | 1.25 (0.91 to 1.72) | - | - |  |
| Adjustment for confounders | |  |  |  |  |  |
|  | Age |  |  |  |  | 0.60 |
|  | Yes | 5 | **1.18 (1.04 to 1.35)** | 45.9 | 0.12 |  |
|  | No | 3 | 1.29 (0.97 to 1.72) | 0.0 | 0.84 |  |
|  | Socioeconomic status |  |  |  |  | 0.48 |
|  | Yes | 3 | 1.24 (0.95 to1.63) | 54.3 | 0.11 |  |
|  | No | 5 | **1.17 (1.07 to 1.27)** | 0.0 | 0.65 |  |
|  | Smoking or alcohol drinking |  |  |  |  | 0.60 |
|  | Yes | 5 | **1.18 (1.04 to 1.35)** | 45.9 | 0.12 |  |
|  | No | 3 | 1.29 (0.97 to 1.72) | 0.0 | 0.84 |  |
|  | Pregnancy BMI |  |  |  |  | 0.48 |
|  | Yes | 1 | 1.06 (0.80 to 1.40) | - | - |  |
|  | No | 7 | **1.21 (1.09 to 1.35)** | 18.3 | 0.29 |  |
|  | Pregnancy complications |  |  |  |  | 0.10 |
|  | Yes | 3 | **1.15 (1.05 to 1.25)** | 0.0 | 0.39 |  |
|  | No | 5 | **1.39 (1.18 to 1.64)** | 0.0 | 0.72 |  |
|  | Parity |  |  |  |  | 0.77 |
|  | Yes | 3 | **1.22 (1.03 to 1.45)** | 69.5 | **0.04** |  |
|  | No | 5 | 1.16 (0.96 to1.39) | 0.0 | 0.84 |  |
| **Congenital heart defects** | | 11 | **1.24 (1.02 to 1.51)** | 52.5 | **0.02** |  |
| Study quality | |  |  |  |  | - |
|  | High risk | - | - | - | - |  |
|  | Low risk | 11 | **1.24 (1.02 to 1.51)** | 52.5 | **0.02** |  |
| Geographic location | |  |  |  |  | 0.29 |
|  | Europe | 8 | 1.16 (0.98 to 1.38) | 41.1 | 0.10 |  |
|  | Northern America | 1 | **6.23 (2.04 to 19.03)** | - | - |  |
|  | Others | 2 | 1.48 (0.77 to 2.85) | 0.0 | 0.98 |  |
| Adjustment for confounders | |  |  |  |  |  |
| Age | |  |  |  |  | 0.41 |
|  | Yes | 7 | 1.18 (0.97 to 1.45) | 48.9 | 0.07 |  |
|  | No | 4 | 1.76 (0.87 to 3.54) | 66.4 | **0.03** |  |
| Socioeconomic status | |  |  |  |  | 0.12 |
|  | Yes | 3 | **1.56 (1.09 to 2.23)** | 31.9 | 0.23 |  |
|  | No | 8 | 1.14 (0.92 to 1.40) | 45.9 | 0.07 |  |
| Smoking or alcohol drinking | |  |  |  |  | 0.41 |
|  | Yes | 7 | 1.18 (0.97 to 1.45) | 48.9 | 0.07 |  |
|  | No | 4 | 1.76 (0.87 to 3.54) | 66.4 | **0.03** |  |
| Pregnancy BMI | |  |  |  |  | 0.78 |
|  | Yes | 1 | 1.13 (0.70 to 1.82) | - | - |  |
|  | No | 10 | **1.26 (1.02 to 1.57)** | 57.2 | **0.01** |  |
| Pregnancy complications | |  |  |  |  | 0.42 |
|  | Yes | 3 | 1.13 (0.99 to 1.29) | 0.0 | 0.89 |  |
|  | No | 8 | 1.43 (1.00 to 2.04) | 64.4 | **0.01** |  |
| Parity | |  |  |  |  | 0.42 |
|  | Yes | 5 | 1.16 (0.91 to 1.48) | 62.7 | **0.03** |  |
|  | No | 6 | 1.47 (1.00 to 2.16) | 48.7 | 0.08 |  |
| Abbreviations: BMI, body mass index; CI, confidence interval; RR, relative risk. *P for heterogeneity within each subgroup. **P for heterogeneity between subgroups with meta-regression analysis. | | | | | | |

| **Table S13. Subgroup analysis of fluoxetine and risk of congenital malformations in infants: results of meta-analyses.** | | | | | | |
| --- | --- | --- | --- | --- | --- | --- |
|  | | **Fluoxetine** | | | | |
|  | | **No. of studies** | **Summary RR (95% CI)** | I^2^, % | ***P**** | P** |
| **Major congenital anomalies** | | 11 | **1.17 (1.07 to 1.28)** | 0.0 | 0.57 |  |
| Study quality | |  |  |  |  | 0.13 |
|  | High risk | 3 | **1.77 (1.07 to 2.92)** | 0.0 | 0.89 |  |
|  | Low risk | 8 | **1.16 (1.06 to 1.27)** | 0.0 | 0.49 |  |
| Geographic location | |  |  |  |  | 0.28 |
|  | Europe | 6 | **1.14 (1.02 to 1.28)** | 18.1 | 0.30 |  |
|  | Northern America | 3 | 1.15 (0.79 to 1.66) | 0.0 | 0.64 |  |
|  | Others | 2 | 1.51 (1.00 to 2.26) | 0.0 | 0.36 |  |
| Adjustment for confounders | |  |  |  |  |  |
|  | Age |  |  |  |  | 0.35 |
|  | Yes | 5 | **1.13 (0.99 to 1.29)** | 32.4 | 0.21 |  |
|  | No | 6 | 1.31 (1.00 to 1.71) | 0.0 | 0.74 |  |
|  | Socioeconomic status |  |  |  |  | 0.07 |
|  | Yes | 3 | 1.00 (0.83 to 1.19) | 0.0 | 0.42 |  |
|  | No | 8 | **1.24 (1.12 to 1.37)** | 0.0 | 0.86 |  |
|  | Smoking or alcohol drinking |  |  |  |  | 0.35 |
|  | Yes | 5 | **1.13 (0.99 to 1.29)** | 32.4 | 0.21 |  |
|  | No | 6 | 1.31 (1.00 to 1.71) | 0.0 | 0.74 |  |
|  | Pregnancy BMI |  |  |  |  | **0.04** |
|  | Yes | 1 | 0.91 (0.73 to 1.14) | - | - |  |
|  | No | 10 | **1.23 (1.11 to 1.35)** | 0.0 | 0.93 |  |
|  | Pregnancy complications |  |  |  |  | 0.50 |
|  | Yes | 3 | 1.12 (0.93 to 1.34) | 64.9 | 0.06 |  |
|  | No | 8 | **1.23 (1.01 to 1.49)** | 0.0 | 0.84 |  |
|  | Parity |  |  |  |  | 0.18 |
|  | Yes | 3 | **1.22 (1.10 to 1.36)** | 0.0 | 0.83 |  |
|  | No | 8 | 1.05 (0.89 to 1.25) | 0.0 | 0.45 |  |
| **Congenital heart defects** | | 14 | **1.30 (1.12 to 1.53)** | 29.3 | 0.14 |  |
| Study quality | |  |  |  |  | 0.08 |
|  | High risk | 1 | **4.47 (1.31 to 15.26)** | - | - |  |
|  | Low risk | 13 | **1.28 (1.11 to 1.46)** | 29.3 | 0.14 |  |
| Geographic location | |  |  |  |  | 0.16 |
|  | Europe | 9 | **1.25 (1.02 to 1.53)** | 38.2 | 0.11 |  |
|  | Northern America | 2 | **1.26 (1.04 to 1.53)** | 0.0 | 0.54 |  |
|  | Others | 3 | **2.50 (1.28 to 4.88)** | 0.0 | 0.54 |  |
| Adjustment for confounders | |  |  |  |  |  |
| Age | |  |  |  |  | 0.62 |
|  | Yes | 8 | **1.37 (1.08 to 1.74)** | 46.6 | 0.07 |  |
|  | No | 6 | **1.22 (1.02 to 1.44)** | 0.0 | 0.48 |  |
| Socioeconomic status | |  |  |  |  | 0.72 |
|  | Yes | 3 | 1.19 (0.58 to 2.44) | 74.4 | **0.02** |  |
|  | No | 11 | **1.30 (1.15 to 1.48)** | 4.1 | 0.40 |  |
| Smoking or alcohol drinking | |  |  |  |  | 0.62 |
|  | Yes | 8 | **1.37 (1.08 to 1.74)** | 46.6 | 0.07 |  |
|  | No | 6 | **1.22 (1.02 to 1.44)** | 0.0 | 0.48 |  |
| Pregnancy BMI | |  |  |  |  | 0.09 |
|  | Yes | 1 | 0.84 (0.55 to 1.29) | - | - |  |
|  | No | 13 | **1.35 (1.17 to 1.56)** | 16.1 | 0.28 |  |
| Pregnancy complications | |  |  |  |  | 0.46 |
|  | Yes | 3 | 1.22 (0.95 to 1.58) | 52.1 | 0.12 |  |
|  | No | 11 | **1.40 (1.10 to 1.77)** | 29.3 | 0.17 |  |
| Parity | |  |  |  |  | 0.20 |
|  | Yes | 5 | **1.41 (1.21 to 1.63)** | 0.0 | 0.42 |  |
|  | No | 9 | 1.20 (0.92 to 1.56) | 32.7 | 0.16 |  |
| Abbreviations: BMI, body mass index; CI, confidence interval; RR, relative risk. *P for heterogeneity within each subgroup. **P for heterogeneity between subgroups with meta-regression analysis. | | | | | | |

| **Table S14. Subgroup analysis of paroxetine and risk of congenital malformations in infants: results of meta-analyses.** | | | | | | |
| --- | --- | --- | --- | --- | --- | --- |
|  | | **Paroxetine** | | | | |
|  | | **No. of studies** | **Summary RR (95% CI)** | I^2^, % | ***P**** | P** |
| **Major congenital anomalies** | | 11 | **1.18 (1.05 to 1.32)** | 0.0 | 0.64 |  |
| Study quality | |  |  |  |  | 0.08 |
|  | High risk | 3 | **1.76 (1.16 to 2.66)** | 0.0 | 0.70 |  |
|  | Low risk | 8 | **1.14 (1.01 to 1.28)** | 0.0 | 0.87 |  |
| Geographic location | |  |  |  |  | 0.97 |
|  | Europe | 7 | **1.19 (1.04 to 1.36)** | 0.0 | 0.97 |  |
|  | Northern America | 2 | 1.00 (0.72 to 1.39) | 0.0 | 0.32 |  |
|  | Others | 2 | 1.40 (0.68 to 2.88) | 76.5 | **0.04** |  |
| Adjustment for confounders | |  |  |  |  |  |
|  | Age |  |  |  |  | 0.82 |
|  | Yes | 6 | **1.19 (1.04 to 1.36)** | 0.0 | 0.95 |  |
|  | No | 5 | 1.22 (0.89 to 1.67) | 39.6 | 0.16 |  |
|  | Socioeconomic status |  |  |  |  | 0.94 |
|  | Yes | 3 | 1.19 (0.94 to 1.50) | 0.0 | 0.70 |  |
|  | No | 8 | **1.18 (1.03 to 1.35)** | 1.7 | 0.42 |  |
|  | Smoking or alcohol drinking |  |  |  |  | 0.93 |
|  | Yes | 5 | **1.18 (1.03 to 1.35)** | 0.0 | 0.94 |  |
|  | No | 6 | 1.23 (0.92 to 1.64) | 29.0 | 0.22 |  |
|  | Pregnancy BMI |  |  |  |  | 0.68 |
|  | Yes | 2 | 1.12 (0.81 to 1.54) | 0.0 | 0.46 |  |
|  | No | 9 | **1.19 (1.05 to 1.34)** | 0.0 | 0.52 |  |
|  | Pregnancy complications |  |  |  |  | 0.74 |
|  | Yes | 3 | 1.16 (1.00 to 1.34) | 0.0 | 0.87 |  |
|  | No | 8 | 1.22 (1.00 to 1.48) | 5.9 | 0.38 |  |
|  | Parity |  |  |  |  | 0.76 |
|  | Yes | 4 | **1.20 (1.03 to 1.39)** | 0.0 | 0.92 |  |
|  | No | 7 | 1.17 (0.96 to 1.44) | 17.1 | 0.30 |  |
| **Congenital heart defects** | | 16 | **1.35 (1.19 to 1.53)** | 0.0 | 0.71 |  |
| Study quality | |  |  |  |  | 0.72 |
|  | High risk | 2 | 1.61 (0.68 to 3.80) | 16.7 | 0.27 |  |
|  | Low risk | 14 | **1.34 (1.18 to 1.53)** | 0.0 | 0.67 |  |
| Geographic location | |  |  |  |  | 0.67 |
|  | Europe | 9 | **1.43 (1.20 to 1.70)** | 0.0 | 0.74 |  |
|  | Northern America | 4 | 1.19 (0.98 to 1.44) | 0.0 | 0.92 |  |
|  | Others | 3 | **2.18 (1.28 to 3.70)** | 0.0 | 0.73 |  |
| Adjustment for confounders | |  |  |  |  |  |
| Age | |  |  |  |  | 0.90 |
|  | Yes | 8 | **1.38 (1.15 to 1.67)** | 0.0 | 0.74 |  |
|  | No | 8 | **1.34 (1.20 to 1.61)** | 2.2 | 0.41 |  |
| Socioeconomic status | |  |  |  |  | 0.39 |
|  | Yes | 3 | **1.62 (1.10 to 2.37)** | 0.0 | 0.64 |  |
|  | No | 13 | **1.32 (1.15 to 1.51)** | 0.0 | 0.64 |  |
| Smoking or alcohol drinking | |  |  |  |  | 0.85 |
|  | Yes | 8 | **1.40 (1.16 to 1.69)** | 0.0 | 0.60 |  |
|  | No | 8 | **1.31 (1.10 to 1.55)** | 0.0 | 0.56 |  |
| Pregnancy BMI | |  |  |  |  | 0.30 |
|  | Yes | 1 | **1.78 (1.10 to 2.89)** | - | - |  |
|  | No | 15 | **1.32 (1.16 to 1.51)** | 0.0 | 0.74 |  |
| Pregnancy complications | |  |  |  |  | 0.76 |
|  | Yes | 3 | **1.34 (1.07 to 1.69)** | 0.5 | 0.37 |  |
|  | No | 13 | **1.35 (1.16 to 1.57)** | 0.0 | 0.65 |  |
| Parity | |  |  |  |  | 0.60 |
|  | Yes | 5 | **1.33 (1.08 to 1.64)** | 0.0 | 0.60 |  |
|  | No | 11 | **1.36 (1.16 to 1.59)** | 0.0 | 0.55 |  |
| Abbreviations: BMI, body mass index; CI, confidence interval; RR, relative risk. *P for heterogeneity within each subgroup. **P for heterogeneity between subgroups with meta-regression analysis. | | | | | | |

| **Table S15. Subgroup analysis of sertraline and risk of congenital malformations in infants: results of meta-analyses.** | | | | | | |
| --- | --- | --- | --- | --- | --- | --- |
|  | | **Sertraline** | | | | |
|  | | **No. of studies** | **Summary RR (95% CI)** | I^2^, % | ***P**** | P** |
| **Major congenital anomalies** | | 9 | 1.10 (0.99 to 1.22) | 0.0 | 0.69 |  |
| Study quality | |  |  |  |  | 0.58 |
|  | High risk | 1 | 0.61 (0.08 to 4.53) | - | - |  |
|  | Low risk | 8 | 1.10 (1.00 to 1.22) | 0.0 | 0.62 |  |
| Geographic location | |  |  |  |  | 0.48 |
|  | Europe | 6 | 1.12 (1.00 to 1.25) | 0.0 | 0.44 |  |
|  | Northern America | 2 | 0.98 (0.64 to 1.51) | 0.0 | 0.64 |  |
|  | Others | 1 | 1.01 (0.72 to 1.41) | - | - |  |
| Adjustment for confounders | |  |  |  |  |  |
|  | Age |  |  |  |  | 0.39 |
|  | Yes | 5 | 1.14 (1.00 to 1.30) | 12.3 | 0.34 |  |
|  | No | 4 | 0.99 (0.76 to 1.28) | 0.0 | 0.95 |  |
|  | Socioeconomic status |  |  |  |  | 0.07 |
|  | Yes | 3 | **1.38 (1.10 to 1.73)** | 0.0 | 0.87 |  |
|  | No | 6 | 1.04 (0.93 to 1.16) | 0.0 | 0.99 |  |
|  | Smoking or alcohol drinking |  |  |  |  | 0.39 |
|  | Yes | 5 | 1.14 (1.00 to 1.30) | 12.3 | 0.34 |  |
|  | No | 4 | 0.99 (0.76 to 1.28) | 0.0 | 0.95 |  |
|  | Pregnancy BMI |  |  |  |  | 0.49 |
|  | Yes | 1 | 1.27 (0.85 to 1.89) | - | - |  |
|  | No | 8 | 1.09 (0.98 to 1.21) | 0.0 | 0.65 |  |
|  | Pregnancy complications |  |  |  |  | 0.43 |
|  | Yes | 3 | 1.07 (0.95 to 1.21) | 0.0 | 0.64 |  |
|  | No | 6 | 1.18 (0.98 to 1.42) | 0.0 | 0.54 |  |
|  | Parity |  |  |  |  | 0.93 |
|  | Yes | 3 | 1.12 (0.94 to 1.33) | 34.1 | 0.22 |  |
|  | No | 6 | 1.11 (0.91 to 1.36) | 0.0 | 0.76 |  |
| **Congenital heart defects** | | 13 | **1.42 (1.12 to 1.80)** | 63.9 | **0.001** |  |
| Study quality | |  |  |  |  | **-** |
|  | High risk | **-** | **-** | **-** | **-** |  |
|  | Low risk | 13 | **1.42 (1.12 to 1.80)** | 63.9 | **0.001** |  |
| Geographic location | |  |  |  |  | 0.50 |
|  | Europe | 9 | 1.41 (1.00 to 1.99) | 71.8 | **<0.001** |  |
|  | Northern America | 2 | 1.26 (1.06 to 1.50) | 0.0 | 0.72 |  |
|  | Others | 2 | 2.84 (0.72 to 11.30) | 57.1 | **0.13** |  |
| Adjustment for confounders | |  |  |  |  |  |
| Age | |  |  |  |  | 0.89 |
|  | Yes | 7 | 1.44 (0.95 to 2.17) | 78.7 | **<0.001** |  |
|  | No | 6 | **1.32 (1.13 to 1.55)** | 0.0 | 0.43 |  |
| Socioeconomic status | |  |  |  |  | 0.08 |
|  | Yes | 3 | **2.28 (1.61 to 3.24)** | 2.7 | 0.36 |  |
|  | No | 10 | 1.24 (0.98 to 1.55) | 52.9 | **0.024** |  |
| Smoking or alcohol drinking | |  |  |  |  | 0.89 |
|  | Yes | 7 | 1.44 (0.95 to 2.17) | 78.7 | **<0.001** |  |
|  | No | 6 | **1.32 (1.13 to 1.55)** | 0.0 | 0.43 |  |
| Pregnancy BMI | |  |  |  |  | 0.93 |
|  | Yes | 1 | 1.52 (0.78 to 2.96) | - | - |  |
|  | No | 12 | **1.42 (1.10 to 1.83)** | 66.7 | **0.001** |  |
| Pregnancy complications | |  |  |  |  | 0.18 |
|  | Yes | 3 | 1.07 (0.75 to 1.53) | 42.5 | 0.18 |  |
|  | No | 10 | **1.63 (1.19 to 2.25)** | 64.6 | **0.003** |  |
| Parity | |  |  |  |  | 0.58 |
|  | Yes | 5 | 1.33 (0.80 to 2.22) | 84.5 | **<0.001** |  |
|  | No | 8 | **1.35 (1.17 to 1.57)** | 0.0 | 0.47 |  |
| Abbreviations: BMI, body mass index; CI, confidence interval; RR, relative risk. *P for heterogeneity within each subgroup. **P for heterogeneity between subgroups with meta-regression analysis. | | | | | | |

References:

1. Ornoy A, Koren G. Selective serotonin reuptake inhibitors in human pregnancy: On the way to resolving the controversy. Semin Fetal Neonatal Med. 2014;19(3):188-94.

2. Berard A, Zhao J, Sheehy O. Antidepressant use during pregnancy and the risk of major congenital malformations in a cohort of depressed pregnant women: an updated analysis of the Quebec Pregnancy Cohort. BMJ Open. 2017;7(1):e013372.

3. Jimenez-Solem E, Andersen JT, Petersen M, Broedbaek K, Andersen NL, Torp-Pedersen C, et al. Prevalence of antidepressant use during pregnancy in Denmark, a nation-wide cohort study. PLoS One. 2013;8(4):e63034.

4. Taouk LH, Matteson KA, Stark LM, Schulkin J. Prenatal depression screening and antidepressant prescription: obstetrician-gynecologists' practices, opinions, and interpretation of evidence. Arch Womens Ment Health. 2018;21(1):85-91.

5. Liu Y, Zhou X, Zhu D, Chen J, Qin B, Zhang Y, et al. Is pindolol augmentation effective in depressed patients resistant to selective serotonin reuptake inhibitors? A systematic review and meta-analysis. Hum Psychopharmacol. 2015;30(3):132-42.

6. Hendrick V, Stowe ZN, Altshuler LL, Hwang S, Lee E, Haynes D. Placental passage of antidepressant medications. Am J Psychiatry. 2003;160(5):993-6.

7. Laine K, Heikkinen T, Ekblad U, Kero P. Effects of exposure to selective serotonin reuptake inhibitors during pregnancy on serotonergic symptoms in newborns and cord blood monoamine and prolactin concentrations. Arch Gen Psychiatry. 2003;60(7):720-6.

8. Sadler TW. Selective serotonin reuptake inhibitors (SSRIs) and heart defects: potential mechanisms for the observed associations. Reprod Toxicol. 2011;32(4):484-9.

9. U.S Food and Drug Administration (FDA). Public Health Advisory: Paroxetine. 2005. https://wayback.archiveit.org/7993/20170112033310/http://www.fda.gov/Drugs/DrugSafety/PostmarketDrugSafetyInformationforPatientsandProviders/ucm051731.htm. Accessed 27 Aug 2018.

10. Nembhard WN, Tang X, Hu Z, MacLeod S, Stowe Z, Webber D. Maternal and infant genetic variants, maternal periconceptional use of selective serotonin reuptake inhibitors, and risk of congenital heart defects in offspring: population based study. BMJ. 2017;356:j832.

11. Zhang TN, Gao SY, Shen ZQ, Li D, Liu CX, Lv HC, et al. Use of selective serotonin-reuptake inhibitors in the first trimester and risk of cardiovascular-related malformations: a meta-analysis of cohort studies. Sci Rep. 2017;7:43085.

12. Selmer R, Haglund B, Furu K, Andersen M, Nørgaard M, Zoëga H, et al. Individual-based versus aggregate meta-analysis in multi-database studies of pregnancy outcomes: the Nordic example of selective serotonin reuptake inhibitors and venlafaxine in pregnancy. Pharmacoepidemiol Drug Saf. 2016;25(10):1160-9.

13. Kowalik E, Ward K, Ye Y. SSRI use in pregnancy and congenital heart defects: A meta-analysis of population-based cohort studies. Pharmacotherapy. 2016;36(12):e302.

14. Wang S, Yang L, Wang L, Gao L, Xu B, Xiong Y. Selective Serotonin Reuptake Inhibitors (SSRIs) and the Risk of Congenital Heart Defects: A Meta-Analysis of Prospective Cohort Studies. J Am Heart Assoc. 2015;4(5):e001681.

15. Myles N, Newall H, Ward H, Large M. Systematic meta-analysis of individual selective serotonin reuptake inhibitor medications and congenital malformations. Aust N Z J Psychiatry. 2013;47(11):1002-12.

16. Bérard A, Iessa N, Chaabane S, Muanda FT, Boukhris T, Zhao JP. The risk of major cardiac malformations associated with paroxetine use during the first trimester of pregnancy: A systematic review and meta-analysis. Br J Clin Pharmacol. 2016;81(4):589-604.

17. Painuly N, Painuly R, Heun R, Sharan P. Risk of cardiovascular malformations after exposure to paroxetine in pregnancy: Meta-analysis. Psychiatrist. 2013;37(6):198-203.

18. Grigoriadis S, VonderPorten EH, Mamisashvili L, Roerecke M, Rehm J, Dennis CL, et al. Antidepressant exposure during pregnancy and congenital malformations: Is there an association? A systematic review and meta-analysis of the best evidence. J Clin Psychiatry. 2013;74(4):e293-308.

19. Wurst KE, Poole C, Ephross SA, Olshan AF. First trimester paroxetine use and the prevalence of congenital, specifically cardiac, defects: A meta-analysis of epidemiological studies. Birth Defects Res A Clin Mol Teratol. 2010;88(3):159-70.

20. Shen ZQ, Gao SY, Li SX, Zhang TN, Liu CX, Lv HC, et al. Sertraline use in the first trimester and risk of congenital anomalies: a systemic review and meta-analysis of cohort studies. Br J Clin Pharmacol. 2017;83(4):909-22.

21. Gao SY, Wu QJ, Zhang TN, Shen ZQ, Liu CX, Xu X, et al. Fluoxetine and congenital malformations: a systematic review and meta-analysis of cohort studies. Br J Clin Pharmacol. 2017;83(10):2134-47.

22. Yan Y, Cheng Y, Crowe B, Chhabra-Khanna R, Camporeale A, Marangell L. First trimester fluoxetine use and major malformations: A meta-analysis of epidemiological studies. Pharmacoepidemiol Drug Saf. 2013;22:168-9.

23. Riggin L, Frankel Z, Moretti M, Pupco A, Koren G. The fetal safety of fluoxetine: a systematic review and meta-analysis. Journal of obstetrics and gynaecology Canada. J Obstet Gynaecol Can. 2013;35(4):362-9.

24. Bar-Oz B, Einarson T, Einarson A, Boskovic R, O'Brien L, Malm H, et al. Paroxetine and congenital malformations: meta-Analysis and consideration of potential confounding factors. Clin Ther. 2007;29(5):918-26.

25. Kang HH, Ahn KH, Hong SC, Kwon BY, Lee EH, Lee JS, et al. Association of citalopram with congenital anomalies: A meta-analysis. Obstet Gynecol Sci. 2017;60(2):145-53.

26. Nikfar S, Rahimi R, Hendoiee N, Abdollahi M. Increasing the risk of spontaneous abortion and major malformations in newborns following use of serotonin reuptake inhibitors during pregnancy: A systematic review and updated meta-analysis. Daru. 2012;20(75).

27. O'Brien L, Einarson TR, Sarkar M, Einarson A, Koren G. Does Paroxetine Cause Cardiac Malformations? J Obstet Gynaecol Can. 2008;30(8):696-701.

28. Addis A, Koren G. Safety of fluoxetine during the first trimester of pregnancy: a meta-analytical review of epidemiological studies. Psychol Med. 2000;30(1):89-94.

29. Reefhuis J, Devine O, Friedman JM, Louik C, Honein MA. Specific SSRIs and birth defects: bayesian analysis to interpret new data in the context of previous reports. BMJ. 2015;351(h3190).

30. Nishigori H, Obara T, Nishigori T, Mizuno S, Metoki H, Hoshiai T, et al. Selective serotonin reuptake inhibitors and risk of major congenital anomalies for pregnancies in Japan: A nationwide birth cohort study of the Japan Environment and Children's Study. Congenit Anom. 2017;57(3):72-8.

31. Jordan S, Morris JK, Davies GI, Tucker D, Thayer DS, Luteijn JM, et al. Selective Serotonin Reuptake Inhibitor (SSRI) antidepressants in pregnancy and congenital anomalies: Analysis of linked databases in Wales, Norway and Funen, Denmark. PLoS One. 2016;11(12).

32. Petersen I, Evans SJ, Gilbert R, Marston L, Nazareth I. Selective serotonin reuptake inhibitors and congenital heart anomalies: Comparative cohort studies of women treated before and during pregnancy and their children. J Clin Psychiatry. 2016;77(1):e36-42.

33. Malm H, Sourander A, Gissler M, Gyllenberg D, Hinkka-Yli-Salomäki S, McKeague IW, et al. Pregnancy complications following prenatal exposure to SSRIs or maternal psychiatric disorders: Results from population-based national register data. Am J Psychiatry. 2015;172(12):1224-32.

34. Furu K, Kieler H, Haglund B, Engeland A, Selmer R, Stephansson O, et al. Selective serotonin reuptake inhibitors and venlafaxine in early pregnancy and risk of birth defects: population based cohort study and sibling design. BMJ. 2015;350:h1798.

35. Knudsen TM, Hansen AV, Garne E, Andersen AMN. Increased risk of severe congenital heart defects in offspring exposed to selective serotonin-reuptake inhibitors in early pregnancy - an epidemiological study using validated EUROCAT data. BMC Pregnancy Childbirth. 2014;14(1).

36. Margulis AV, Abou-Ali A, Strazzeri MM, Ding Y, Kuyateh F, Frimpong EY, et al. Use of selective serotonin reuptake inhibitors in pregnancy and cardiac malformations: A propensity-score matched cohort in CPRD. Pharmacoepidemiol Drug Saf. 2013;22(9):942-51.

37. Jimenez-Solem E, Andersen JT, Petersen M, Broedbaek K, Jensen JK, Afzal S, et al. Exposure to selective serotonin reuptake inhibitors and the risk of congenital malformations: A nationwide cohort study. BMJ open. 2012;2(3).

38. Nordeng H, Van Gelder MMHJ, Spigset O, Koren G, Einarson A, Eberhard-Gran M. Pregnancy outcome after exposure to antidepressants and the role of maternal depression: Results from the Norwegian mother and child cohort study. J Clin Psychopharmacol. 2012;32(2):186-94.

39. Malm H, Artama M, Gissler M, Ritvanen A. Selective serotonin reuptake inhibitors and risk for major congenital anomalies. Obstet Gynecol. 2011;118(1):111-20.

40. Colvin L, Slack-Smith L, Stanley FJ, Bower C. Dispensing patterns and pregnancy outcomes for women dispensed selective serotonin reuptake inhibitors in pregnancy. Birth Defects Res A Clin Mol Teratol. 2011;91(4):268.

41. Petersen I, Gilbert R, Evans S, Marston L, Nazareth I. SSRI and risk of congenital cardiac abnormalities. Pharmacoepidemiol Drug Saf. 2010;19:S211.

42. Kornum JB, Nielsen RB, Pedersen L, Mortensen PB, Norgaard M. Use of selective serotonin-reuptake inhibitors during early pregnancy and risk of congenital malformations: updated analysis. Clin Epidemiol. 2010;2:29-36.

43. Pedersen LH, Henriksen TB, Vestergaard M, Olsen J, Bech BH. Selective serotonin reuptake inhibitors in pregnancy and congenital malformations: population based cohort study. BMJ. 2009;339:b3569.

44. Merlob P, Birk E, Sirota L, Linder N, Berant M, Stahl B, et al. Are selective serotonin reuptake inhibitors cardiac teratogens? Echocardiographic screening of newborns with persistent heart murmur. Birth Defects Res A Clin Mol Teratol. 2009;85(10):837-41.

45. Diav-Citrin O, Shechtman S, Weinbaum D, Wajnberg R, Avgil M, Di Gianantonio E, et al. Paroxetine and fluoxetine in pregnancy: A prospective, multicentre, controlled, observational study. Br J Clin Pharmacol. 2008;66(5):695-705.

46. Kallen BA, Otterblad OP. Maternal use of selective serotonin re-uptake inhibitors in early pregnancy and infant congenital malformations. Birth Defects Res A Clin Mol Teratol.2007;79(4):301-8.

47. Vial T, Cournot MP, Bernard N, Carlier P, Jonville-Bero AP, Jean-Pastor MJ, et al. Paroxetine and congenital malformations: a prospective comparative study. Drug Safety. 2006;29(10):970.

48. Grote NK, Bridge JA, Gavin AR, Melville JL, Iyengar S, Katon WJ. A meta-analysis of depression during pregnancy and the risk of preterm birth, low birth weight, and intrauterine growth restriction. Arch Gen Psychiatry. 2010;67(10):1012-24.

49. Szegda K, Markenson G, Bertone-Johnson ER, Chasan-Taber L. Depression during pregnancy: a risk factor for adverse neonatal outcomes? A critical review of the literature. J Matern Fetal Neonatal Med. 2014;27(9):960-7.

50. Ogunyemi D, Jovanovski A, Liu J, Friedman P, Sugiyama N, Creps J, et al. The Contribution of Untreated and Treated Anxiety and Depression to Prenatal, Intrapartum, and Neonatal Outcomes. AJP Rep. 2018;8(3):e146-57.

51. Pedersen LH. The risks associated with prenatal antidepressant exposure: time for a precision medicine approach. Expert Opin Drug Saf. 2017;16(8):915-21.

52. Susser LC, Sansone SA, Hermann AD. Selective serotonin reuptake inhibitors for depression in pregnancy. Am J Obstet Gynecol. 2016;215(6):722-30.

53. Koren G, Nordeng H. Antidepressant use during pregnancy: The benefit-risk ratio. Am J Obstet Gynecol. 2012;207(3):157-63.

54. Moher D, Liberati A, Tetzlaff J, Altman DG. Preferred reporting items for systematic reviews and meta-analyses: the PRISMA statement. BMJ. 2009;339:b2535.

55. Huybrechts KF, Palmsten K, Avorn J, Cohen LS, Holmes LB, Franklin JM, et al. Antidepressant use in pregnancy and the risk of cardiac defects. N Engl J Med. 2014;370(25):2397-407.

56. Davis RL, Rubanowice D, McPhillips H, Raebel MA, Andrade SE, Smith D, et al. Risks of congenital malformations and perinatal events among infants exposed to antidepressant medications during pregnancy. Pharmacoepidemiol Drug Saf. 2007;16(10):1086-94.

57. Chambers CD, Johnson KA, Dick LM, Felix RJ, Jones KL. Birth outcomes in pregnant women taking fluoxetine. N Engl J Med. 1996;335(14):1010-5.

58. Vasilakis-Scaramozza C, Aschengrau A, Cabral H, Jick SS. Antidepressant use during early pregnancy and the risk of congenital anomalies. Pharmacotherapy. 2013;33(7):693-700.

59. Klieger-Grossmann C, Weitzner B, Panchaud A, Pistelli A, Einarson T, Koren G, et al. Pregnancy outcomes following use of escitalopram: A prospective comparative cohort study. J Clin Pharmacol. 2012;52(5):766-70.

60. Einarson A, Choi J, Einarson TR, Koren G. Incidence of major malformations in infants following antidepressant exposure in pregnancy: Results of a large prospective cohort study. Can J Psychiatry. 2009;54(4):242-6.

61. Oberlander TF, Warburton W, Misri S, Riggs W, Aghajanian J, Hertzman C. Major congenital malformations following prenatal exposure to serotonin reuptake inhibitors and benzodiazepines using population-based health data. Birth Defects Res B Dev Reprod Toxicol. 2008;83(1):68-76.

62. Wells GA, Shea BJ, O'Connell D, Peterson J, Welch V, Losos M, et al. The Newcastle-Ottawa Scale (NOS) for Assessing the Quality of Non-Randomized Studies in Meta-Analysis. Appl Eng Agric. 2014;18(6):727-34.

63. Odutayo A, Wong CX, Hsiao AJ, Hopewell S, Altman DG, Emdin CA. Atrial fibrillation and risks of cardiovascular disease, renal disease, and death: systematic review and meta-analysis. BMJ. 2016;354:i4482.

64. Bérard A, Zhao JP, Sheehy O. Sertraline use during pregnancy and the risk of major malformations. Am J Obstet Gynecol 2015;212(6):791-5.

65. Hamling J, Lee P, Weitkunat R, Ambuhl M. Facilitating meta-analyses by deriving relative effect and precision estimates for alternative comparisons from a set of estimates presented by exposure level or disease category. Stat Med. 2008;27(7):954-70.

66. Einarson A, Pistelli A, DeSantis M, Malm H, Paulus WD, Panchaud A, et al. Evaluation of the risk of congenital cardiovascular defects associated with use of paroxetine during pregnancy. Am J Psychiatry. 2008;165(6):749-52.

67. Rothman KJ, Development DD. Modern Epidemiology. 3rd Edition. Wolters Kluwer: Lippincott Williams & Wilkins; 2014.

68. Dersimonian R, Laird N. Meta-analysis in clinical trials. Controlled Clinical Trials. 1986;7(3):177.

69. Higgins JP, Thompson SG, Deeks JJ, Altman DG. Measuring inconsistency in meta-analyses. BMJ. 2003;327(7414):557-60.

70. Begg CB, Mazumdar M. Operating characteristics of a rank correlation test for publication bias. BIOMETRICS. 1994;50(4):1088-101.

71. Egger M, Davey SG, Schneider M, Minder C. Bias in meta-analysis detected by a simple, graphical test. BMJ. 1997;315(7109):629-34.

72. Ban L, Gibson JE, West J, Fiaschi L, Sokal R, Smeeth L, et al. Maternal depression, antidepressant prescriptions, and congenital anomaly risk in offspring: a population-based cohort study. BJOG. 2014;121(12):1471-81.

73. Sari Y, Zhou FC. Serotonin and its transporter on proliferation of fetal heart cells. Int J Dev Neurosci. 2003;21(8):417-24.

74. Sit DK, Perel JM, Helsel JC, Wisner KL. Changes in antidepressant metabolism and dosing across pregnancy and early postpartum. J Clin Psychiatry. 2008;69(4):652-8.

75. Yavarone MS, Shuey DL, Tamir H, Sadler TW, Lauder JM. Serotonin and cardiac morphogenesis in the mouse embryo. Teratology. 1993;47(6):573-84.

76. Choi DS, Kellermann O, Richard S, Colas JF, Bolanos-Jimenez F, Tournois C, et al. Mouse 5-HT2B receptor-mediated serotonin trophic functions. Ann N Y Acad Sci. 1998;861:67-73.

77. Gentile S. Early pregnancy exposure to selective serotonin reuptake inhibitors, risks of major structural malformations, and hypothesized teratogenic mechanisms. Expert Opin Drug Metab Toxicol. 2015;11(10):1585-97.

78. Carmi R, Gohar J, Meizner I, Katz M. Spontaneous abortion--high risk factor for neural tube defects in subsequent pregnancy. Am J Med Genet. 1994;51(2):93-7.

79. Bukowski R, Carpenter M, Conway D, Coustan D, Dudley DJ, Goldenberg RL, et al. Causes of Death Among Stillbirths. JAMA. 2011;306(22):2459-68.

80. Ehrenstein V, Sorensen HT, Bakketeig LS, Pedersen L. Medical databases in studies of drug teratogenicity: methodological issues. Clin Epidemiol. 2010;2:37-43.

81. Tuccori M, Montagnani S, Testi A, Ruggiero E, Mantarro S, Scollo C, et al. Use of selective serotonin reuptake inhibitors during pregnancy and risk of major and cardiovascular malformations: An update. Postgrad Med. 2010;122(4):49-65.

82. Alwan S, Friedman JM. Safety of selective serotonin reuptake inhibitors in pregnancy. CNS Drugs. 2009;23(6):493-509.

83. Alwan S, Friedman JM, Chambers C. Safety of Selective Serotonin Reuptake Inhibitors in Pregnancy: A Review of Current Evidence. CNS Drugs. 2016;30(6):499-515.

84. Ip Q, Smith KW, Malone DC: Bayesian analysis of malformation outcome in selective serotonin reuptake inhibitor (SSRI) use during pregnancy: An indirect comparison of citalopram, fluoxetine, paroxetine, and sertraline. Value in Health 2013; 16:A544.
