# Supplementary figures and images for: Selective serotonin reuptake inhibitor use during early pregnancy and congenital malformations: a systematic review and meta-analysis of cohort studies of more than 9 million births
Source: BMC Med. 2018 Nov 12;16:205. doi: 10.1186/s12916-018-1193-5 (PMC6231277; doi:10.1186/s12916-018-1193-5)

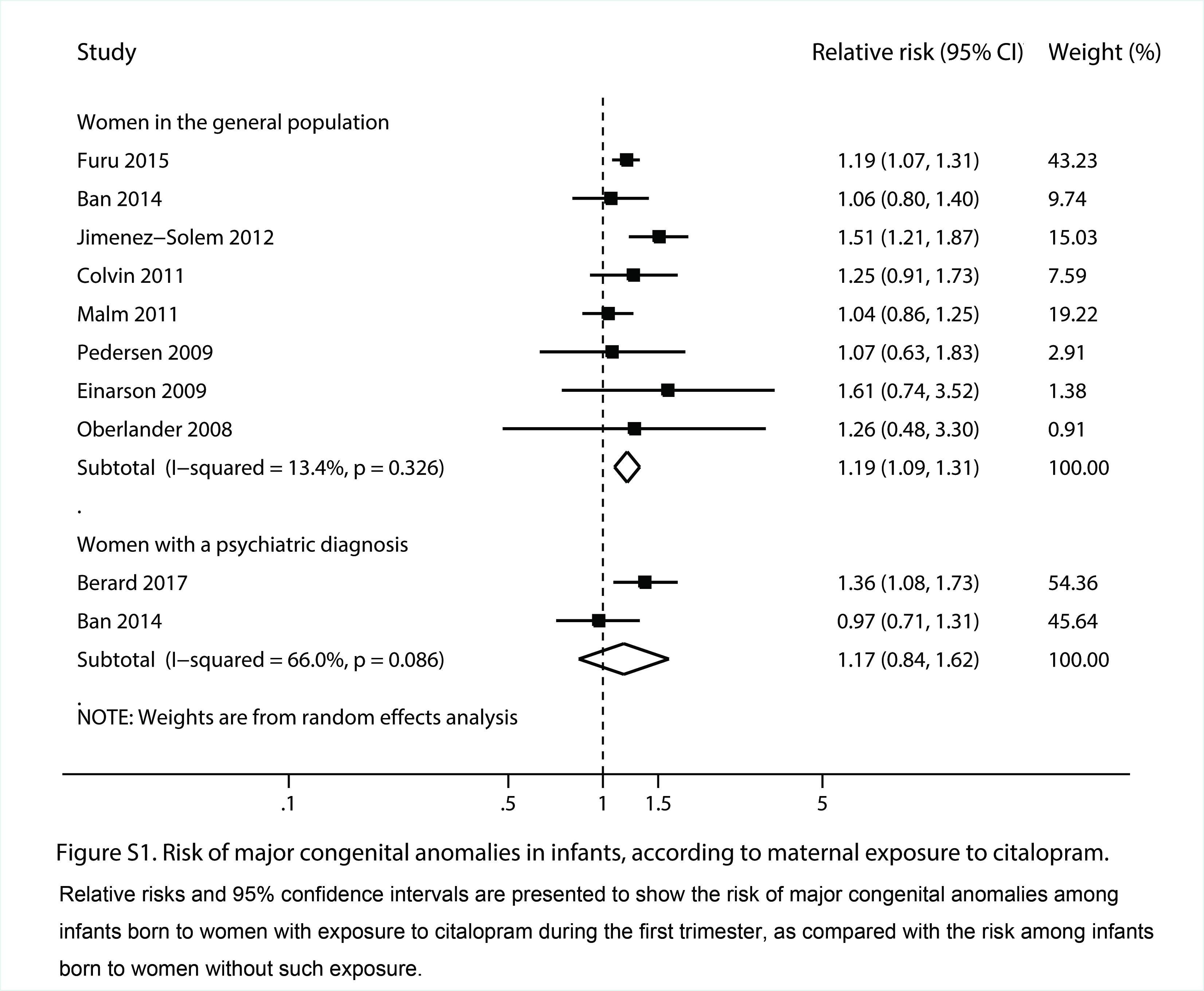

Supplement: Supplementary file 4 — Figure S1. Risk of major congenital anomalies in infants, according to maternal exposure to citalopram. (TIF 1083 kb) [file 12916_2018_1193_MOESM4_ESM.tif]

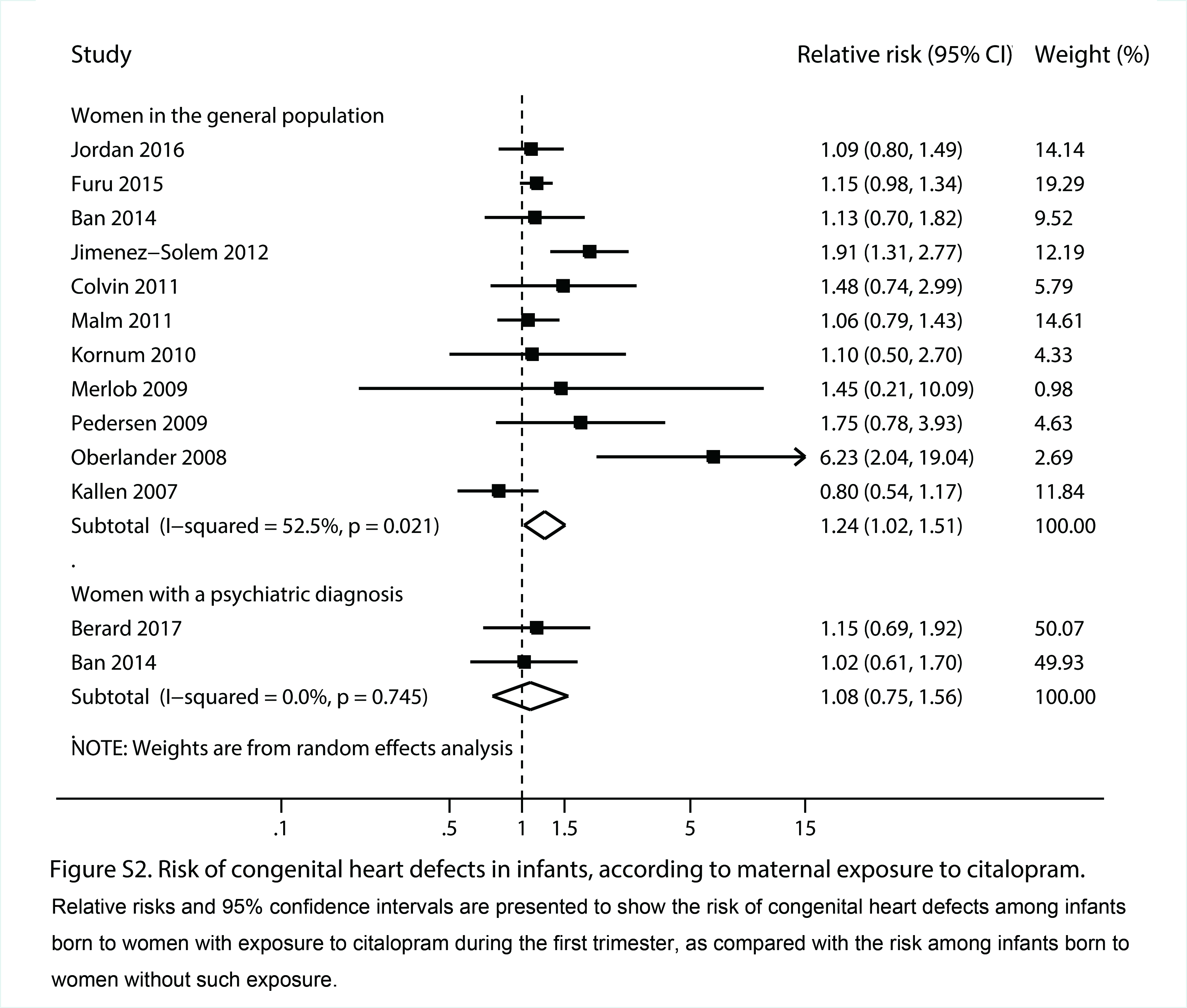

Supplement: Supplementary file 5 — Figure S2. Risk of congenital heart defects in infants, according to maternal exposure to citalopram. (TIF 1123 kb) [file 12916_2018_1193_MOESM5_ESM.tif]

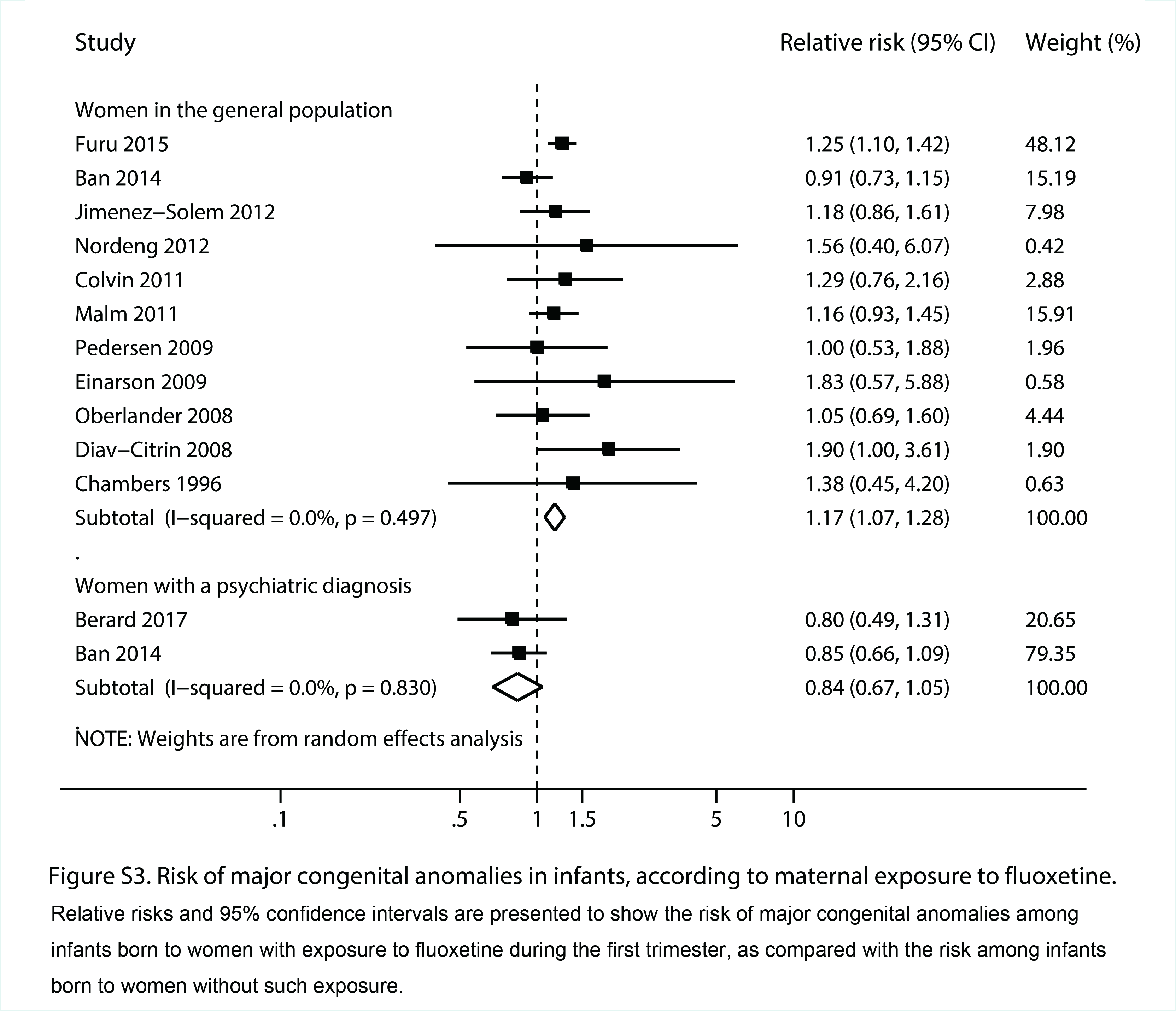

Supplement: Supplementary file 6 — Figure S3. Risk of major congenital anomalies in infants, according to maternal exposure to fluoxetine. (TIF 1141 kb) [file 12916_2018_1193_MOESM6_ESM.tif]

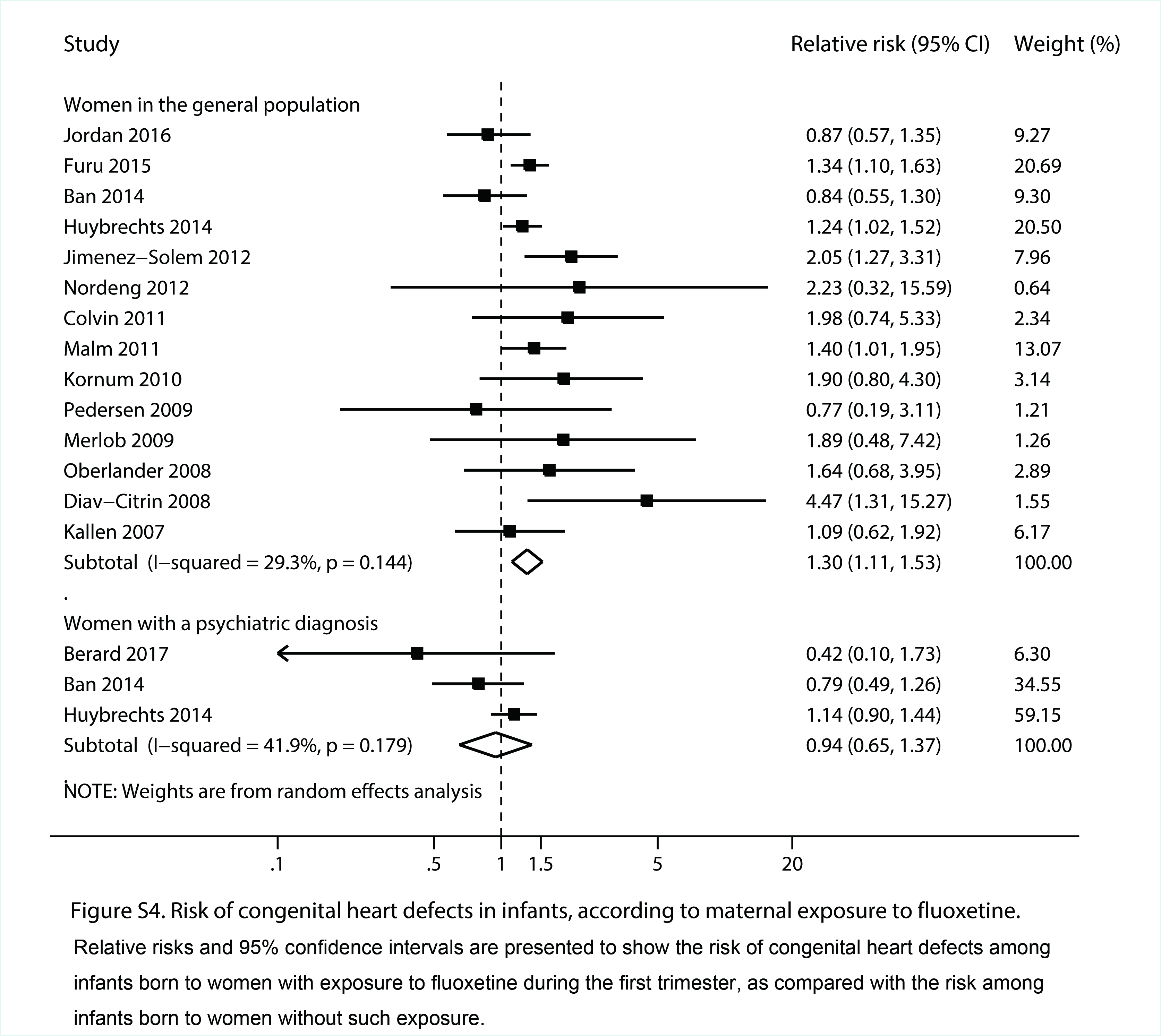

Supplement: Supplementary file 7 — Figure S4. Risk of congenital heart defects in infants, according to maternal exposure to fluoxetine. (TIF 1177 kb) [file 12916_2018_1193_MOESM7_ESM.tif]

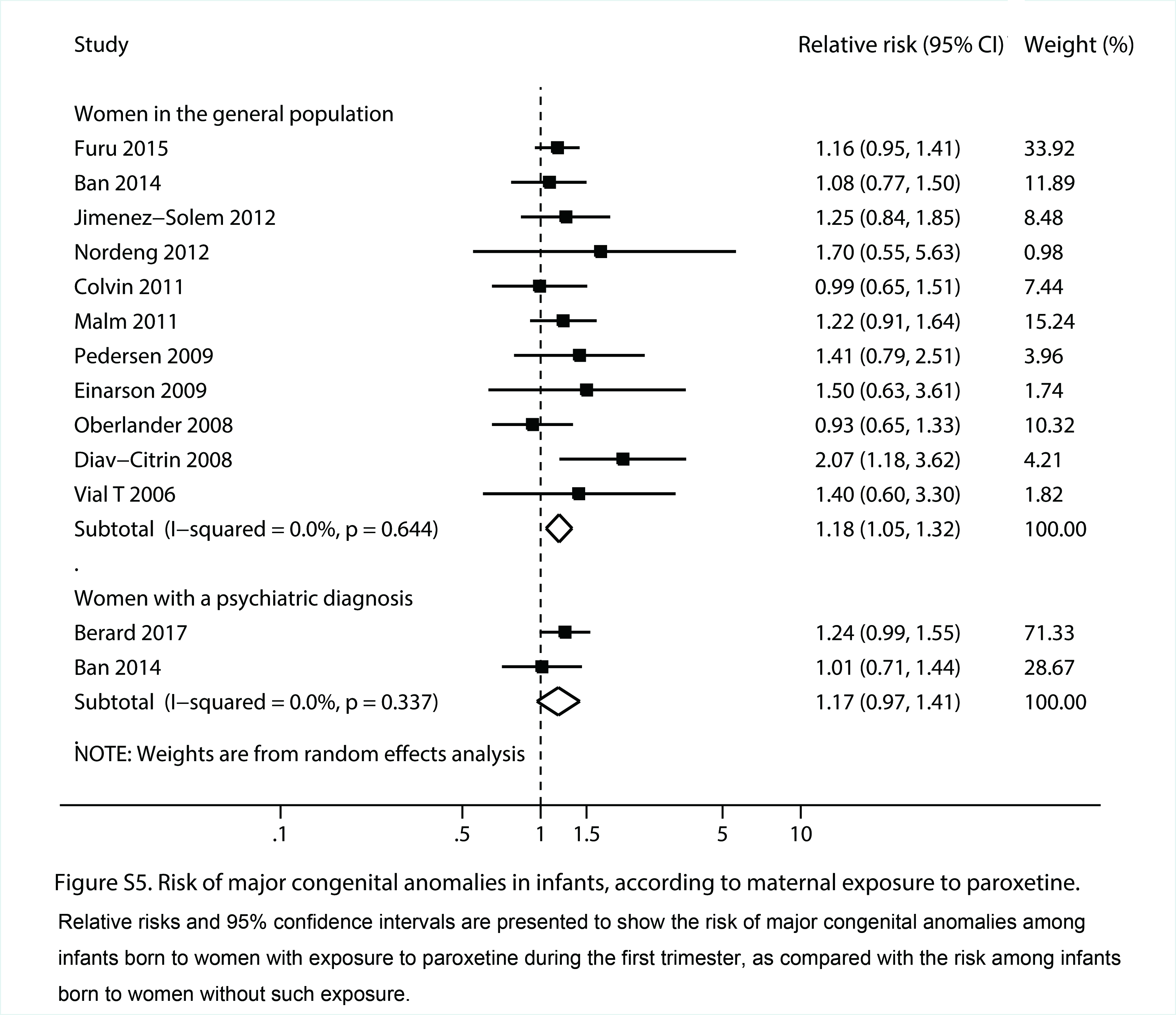

Supplement: Supplementary file 8 — Figure S5. Risk of major congenital anomalies in infants, according to maternal exposure to paroxetine. (TIF 1135 kb) [file 12916_2018_1193_MOESM8_ESM.tif]

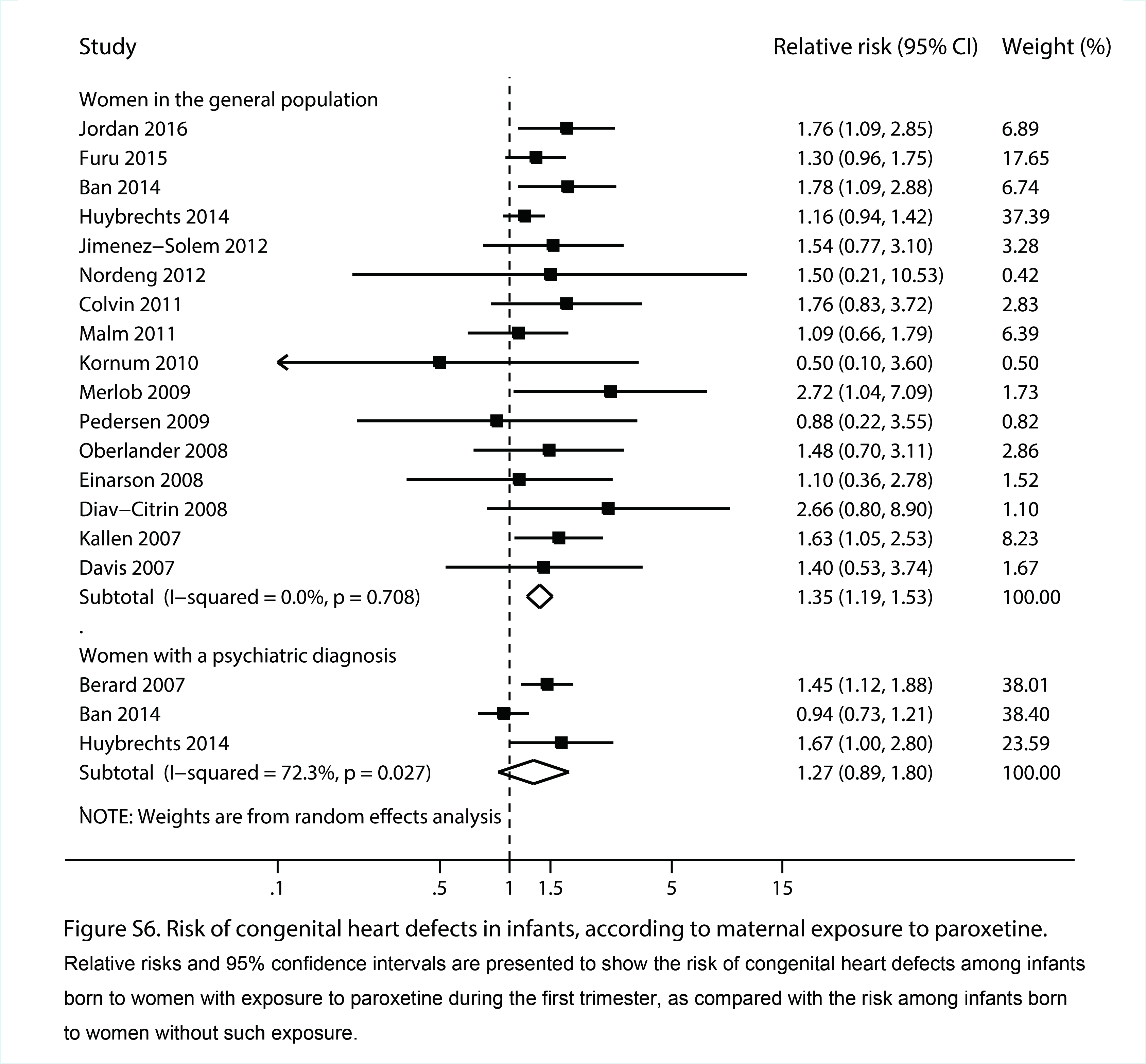

Supplement: Supplementary file 9 — Figure S6. Risk of congenital heart defects in infants, according to maternal exposure to paroxetine. (TIF 1244 kb) [file 12916_2018_1193_MOESM9_ESM.tif]

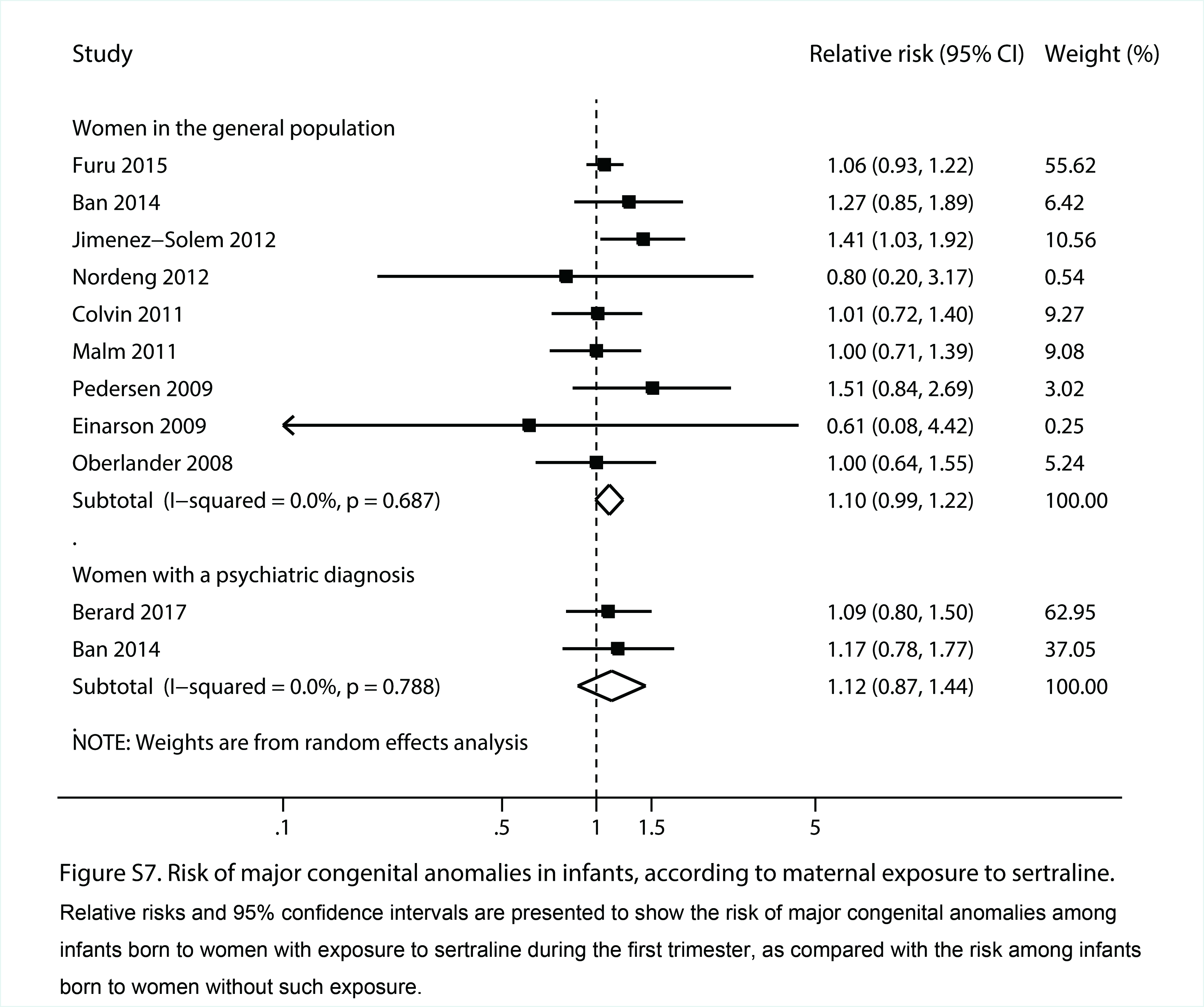

Supplement: Supplementary file 10 — Figure S7. Risk of major congenital anomalies in infants, according to maternal exposure to sertraline. (TIF 1112 kb) [file 12916_2018_1193_MOESM10_ESM.tif]

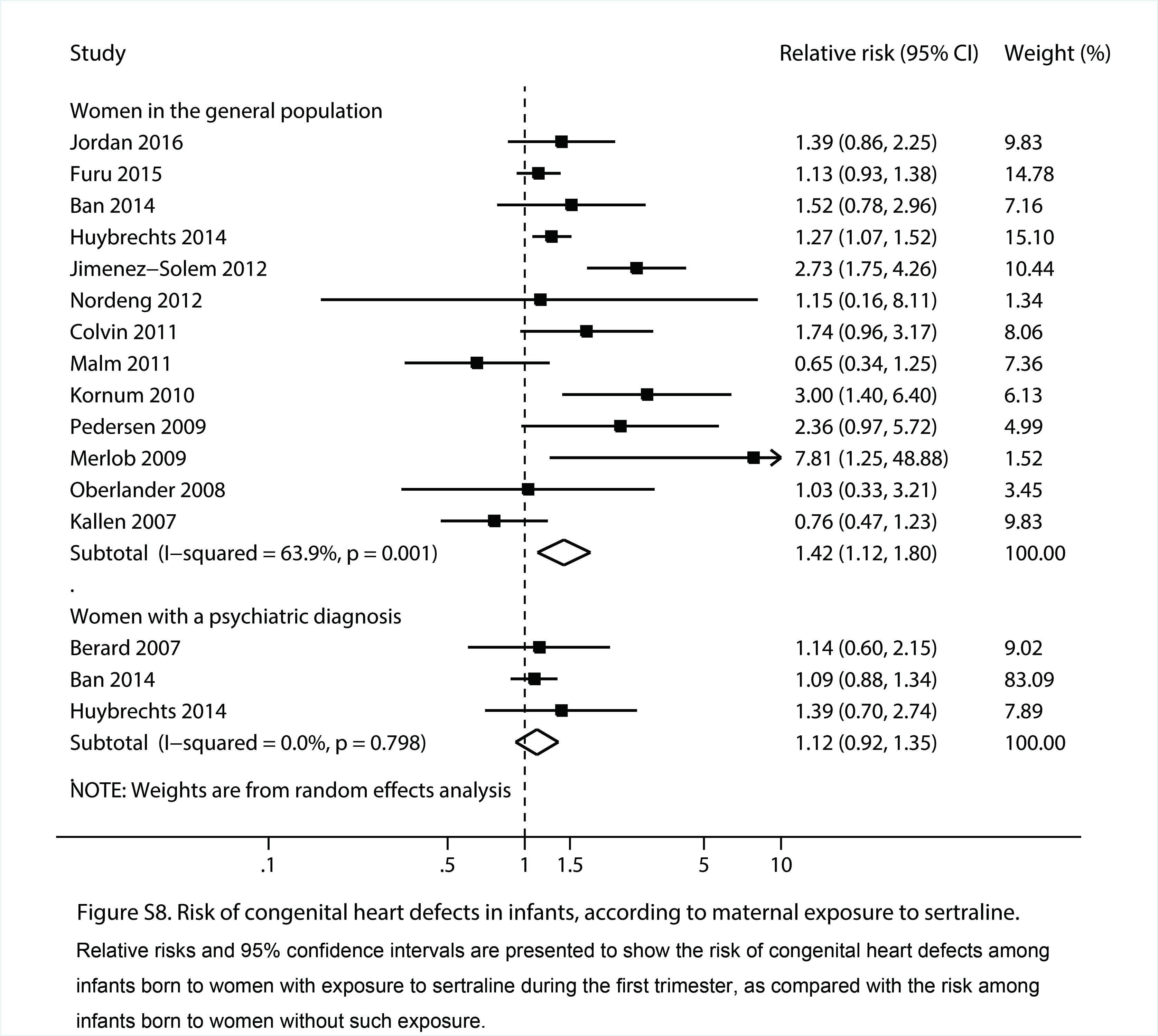

Supplement: Supplementary file 11 — Figure S8. Risk of congenital heart defects in infants, according to maternal exposure to sertraline. (TIF 1185 kb) [file 12916_2018_1193_MOESM11_ESM.tif]
